# Shared functional impairment in the prefrontal cortex affects symptom severity across psychiatric disorders

**DOI:** 10.1017/S0033291720004742

**Published:** 2022-10

**Authors:** Shinsuke Koike, Eisuke Sakakibara, Yoshihiro Satomura, Hanako Sakurada, Mika Yamagishi, Jun Matsuoka, Naohiro Okada, Kiyoto Kasai

**Affiliations:** 1 University of Tokyo Institute for Diversity & Adaptation of Human Mind (UTIDAHM), Meguro-ku, Tokyo 153-8902, Japan; 2 Center for Evolutionary Cognitive Sciences, Graduate School of Art and Sciences, The University of Tokyo, Meguro-ku, Tokyo 153-8902, Japan; 3 University of Tokyo Center for Integrative Science of Human Behavior (CiSHuB), 3-8-1 Komaba, Meguro-ku, Tokyo 153-8902, Japan; 4 The International Research Center for Neurointelligence (WPI-IRCN), Institutes for Advanced Study (UTIAS), University of Tokyo, 7-3-1 Hongo, Bunkyo-ku, Tokyo 113-8654, Japan; 5 Department of Neuropsychiatry, Graduate School of Medicine, University of Tokyo, Bunkyo-ku, Tokyo 113-8655, Japan

**Keywords:** Biological markers, bipolar disorder, functional neuroimaging, major depressive disorder, near-infrared spectroscopy, schizophrenia

## Abstract

**Background:**

The prefrontal deficits in psychiatric disorders have been investigated using functional neuroimaging tools; however, no studies have tested the related characteristics across psychiatric disorders considering various demographic and clinical confounders.

**Methods:**

We analyzed 1558 functional brain measurements using a functional near-infrared spectroscopy during a verbal fluency task from 1200 participants with three disease spectra [196 schizophrenia, 189 bipolar disorder (BPD), and 394 major depressive disorder (MDD)] and 369 healthy controls along with demographic characteristics (age, gender, premorbid IQ, and handedness), task performance during the measurements, clinical assessments, and medication equivalent doses (chlorpromazine, diazepam, biperiden, and imipramine) in a consistent manner. The association between brain functions and demographic and clinical variables was tested using a general linear mixed model (GLMM). Then, the direction of relationship between brain activity and symptom severity, controlling for any other associations, was estimated using a model comparison of structural equation models (SEMs).

**Results:**

The GLMM showed a shared functional deficit of brain activity and a schizophrenia-specific delayed activity timing in the prefrontal cortex (false discovery rate-corrected *p* < 0.05). Comparison of SEMs showed that brain activity was associated with the global assessment of functioning scores in the left inferior frontal gyrus opercularis (IFGOp) in BPD group and the bilateral superior temporal gyrus and middle temporal gyrus, and the left superior frontal gyrus, inferior frontal gyrus triangularis, and IFGOp in MDD group.

**Conclusion:**

This cross-disease large-sample neuroimaging study with high-quality clinical data reveals a robust relationship between prefrontal function and behavioral outcomes across three major psychiatric disorders.

## Introduction

Mental illness is a common disease that around 20% of the general population encounter through their lives (Kessler, Chiu, Demler, Merikangas, & Walters, 2005). Cognitive deficits related to the prefrontal cortex (PFC) are among the most common symptoms and are thought to be related to symptom severity and clinical outcome (Sawada et al., 2017; Velthorst et al., 2019). Previous neuroimaging studies have reported that cognitive deficits in schizophrenia were associated with volumetric and functional alterations in the PFC (Koike, Nishimura, Takizawa, Yahata, & Kasai, 2013; Weinberg et al., 2016). Additionally, these alterations were seen in other psychiatric disorders including bipolar disorder (BPD) and major depressive disorder (MDD), suggesting a shared dysfunction across diseases (Barch & Sheffield, 2014; Kinou et al., 2013; Suto, Fukuda, Ito, Uehara, & Mikuni, 2004). It should be noted that these findings have been mostly derived from case–control studies; therefore, a direct comparison between the aforementioned diseases may shed some light on the common and disease-specific pathologies of psychiatric disorders (Hibar et al., 2015; O'Donovan & Owen, 2016; Ohi et al., 2019).

When conducting a cross-disease mega study in brain imaging, brain-related confounding factors should be considered including age, gender, premorbid intelligence quotient (IQ), clinical severity, and drug treatment. Brain volume and function have been previously associated with sex (Chou et al., 2015a; Gauthier, Duyme, Zanca, & Capron, 2009; Kameyama, Fukuda, Uehara, & Mikuni, 2004; Koike et al., 2017), age (Chou et al., 2015a; Gogtay et al., 2004; Koike et al., 2017), symptom severity (Iwashiro et al., 2012; Koike et al., 2013a), and medication doses (Andreasen, Liu, Ziebell, Vora, & Ho, 2013; Tomioka et al., 2015). However, few studies have considered that these variables are also correlated. For example, men with schizophrenia have more severe symptoms compared to women with schizophrenia, but men generally have a larger brain volume compared to women. Therefore, cautious interpretation is needed when greater brain volume is associated with better functional outcomes. Previous mega studies from multi-centers have had difficulty assessing a high-quality clinical dataset using a uniformed procedure. To maximize the strength of a large sample size, using a well-structured model to properly investigate the relationship between obtained neuroimaging signals and clinical assessments may provide new insights.

Functional near-infrared spectroscopy (fNIRS) is a portable functional neuroimaging instrument that easily and non-invasively measures hemoglobin changes over the surface of the cortex (Koike et al., 2013a). Near-infrared light (650–1000 nm) emitted from a source probe on the human scalp is partially absorbed by hemoglobin in small vessels (<1 mm) and the remaining light is scattered; then, a detector probe can perceive the scattered near-infrared light placed 3 cm away from the source probe in adults (Koike et al., 2013a). The relative advantages of fNIRS technology compared to other neuroimaging instruments are its small size that allows it to be used in places such as schools and care units, low noise levels, and the ability to serve as a candidate resource to differentiate diagnoses (Koike et al., 2017; Suto et al., 2004; Takizawa et al., 2014) and evaluate clinical symptoms (Koike et al., 2013a, 2016). Also, compared to functional MRI, fNIRS can measure hemodynamic responses with high temporal resolution in a non-restricted and natural position.

Previous fNIRS studies in psychiatric disorders have been used to measure brain activity in the frontal and temporal cortices during a block-designed phonological verbal fluency task (VFT) (Koike et al., 2011, 2013b, 2016, 2017; Satomura et al., 2019; Takizawa et al., 2008, 2014). The VFT needs continuous word generation and exercises various cognitive domains involved in verbal storage, verbal working memory, inhibition, and executive control to avoid repetition and inappropriate word use. Therefore, the fNIRS measurement during the VFT can spatio-temporally measure hemoglobin changes in the PFC and anterior and superior parts of the temporal cortex. Investigating the activity pattern using a block-designed task, patients with chronic schizophrenia exhibited smaller activity with inappropriate and delayed responses in the PFC in Japan (Kinou et al., 2013; Koike et al., 2013a, 2016; Shimodera et al., 2012; Suto et al., 2004; Takizawa et al., 2008), China (Li, Wang, Quan, Wu, & Lv, 2015; Quan et al., 2015), and Taiwan (Chou et al., 2015b, 2016; Chou, Lin, Li, Huang, & Sun, 2017). We also found that patients with various clinical stages of schizophrenia, such as first-episode psychosis and ultra-high-risk for psychosis (UHR), had similar characteristics of brain activity to those with chronic schizophrenia, and the characteristics were still present in 12-month follow-up data (Koike et al., 2017). In contrast, patients with MDD showed smaller activation but no delayed responses (Kinou et al., 2013; Suto et al., 2004). Moreover, a multi-center study with more than 1000 participants (153 with MDD, 136 with schizophrenia, 134 with BPD, and 590 healthy controls) replicated the characteristics of brain activity in a cross-disease comparison (Takizawa et al., 2014). The sites, however, obtained via different clinical assessment, were only tested using cross-sectional measurements for patients with chronic stages and the detailed relationships with confounding factors were not tested.

Several fNIRS studies also reported an association between symptom severity, symptom severity, and brain activity, especially in the PFC. Patients with schizophrenia have a relationship between fNIRS brain activity in the rostral part of the PFC and symptom severity based on cross-sectional (Koike et al., 2011, 2013b; Shimodera et al., 2012; Takizawa et al., 2008) and longitudinal investigations (Koike et al., 2016). Patients with MDD also showed a similar relationship in the right PFC in cross-sectional (Noda et al., 2012) and longitudinal studies (Satomura et al., 2019). These studies suggest a relationship between fNIRS prefrontal activity and symptom severity across psychiatric disorders. However, since we have been unable to determine a common and disease-specific relationship using case–control studies, a cross-disease, large-sample study is required.

**Fig. 1.**
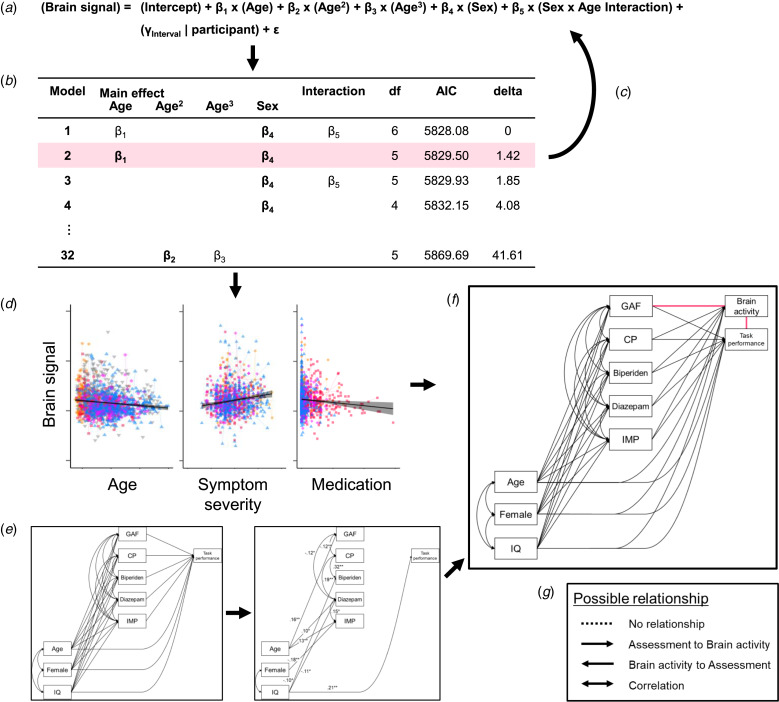
The analysis procedure. (*a*) We applied a model comparison from general linear mixed models (GLMMs) to explore the fixed effect of demographic variables (*β*) and random effect of participants (*γ*) on brain signals. For example, five independent variables (main effects of sex, age, age^2^, and age^3^, and sex × age interaction) were included in an initial model, 32 ( = 2^5^) possible models were compared. (*b*) The model where all the coefficients were significant (*p* < 0.05) and that had the smallest Akaike information criterion (AIC) was defined as the best-fitted model. In this example, the model with the smallest AIC included main effects of age (*β*_1_) and sex (*β*_4_) and sex × age interaction (*β*_5_), but *β*_1_ or *β*_5_ were not significant. Therefore, the second smallest AIC model was applied. (*c*) After testing for all brain signals, we next tested the effect of other variables. (*d*) These model comparisons showed that age, symptom severity, and medication doses were associated with brain activity, but these variables were also correlated with each other. (*e*) Therefore, we applied the structured equation model (SEM) to find the relationships between demographic and clinical variables and task performance. This model included no fNIRS variables and was determined one model for each group. (*f*) Then, we added fNIRS variables to the models. Since the directions of the relationships between brain activity and symptom severity as well as between brain activity and task performance were unable to be determined (pink lines), we performed a model comparison in the SEMs. (*g*) One relationship contained four possible paths: (1) no relationship, (2) path from task or symptom assessment to brain activity, (3) path from brain activity to the assessment, and (4) correlation between them. Therefore, we compared 16 (4 × 4) models for each fNIRS variable in each group. CP, chlorpromazine; IMP, imipramine.

After more than 12 years of fNIRS measurements acquired using a single instrument and procedure, we now have an opportunity to explore generalizable shared and disease-specific alterations in prefrontal function and the relationship with symptom severity among psychiatric disorders. To the best of our knowledge, no neuroimaging studies have tested the relationship between brain function and various demographic and clinical assessments for three major psychiatric diseases with longitudinal measurements. In the present study, we tested whether brain function would be associated with these disease spectra (schizophrenia, BPD, and MDD) considering the effect of demographic and clinical variables on brain function. Further, we wanted to observe whether brain functions measured using fNIRS would be associated with global clinical severity, after controlling for a variety of covariates, and whether the relationship would be common or disease-specific. To utilize a large and high-quality sample dataset from one long-lasting fNIRS project, we applied epidemiological approaches to properly analyze repeated measures and multiple variables using a general linear mixed model (GLMM) and a structural equation model (SEM) (Fig. 1).

## Methods

### Participants

A total of 1772 fronto-temporal brain activity measurements from 1390 participants were measured during a phonological VFT using an fNIRS instrument from April 2004 to August 2018. After the exclusion of poor measurements (see the fNIRS signal treatment section), 1558 measurements were analyzed from 1200 participants [369 controls, 52 UHR, 196 with schizophrenia (51 first-episode psychosis or schizophrenia, and 145 with chronic schizophrenia), 189 with BPD (86 type I and 103 type II), and 394 with MDD; online Supplementary Table S1]. The participants in the patient groups were mainly recruited in the outpatient and inpatient units of the University of Tokyo Hospital. All patients were diagnosed by trained psychiatrists and/or using structured interviews and all healthy controls were screened using structured interviews (see Supplementary materials). A part of the recruitment process was conducted via the integrative neuroimaging studies in schizophrenia targeting for early intervention and prevention (IN-STEP) (Koike et al., 2013b) project, the 4-day psychiatric assessment program for depressive symptoms (Satomura et al., 2019), and a population-based survey [the Japanese Study of Stratification, Health, Income, and Neighborhood (J-SHINE)] (Kawasaki et al., 2015). The first two projects assessed brain activity and clinical assessment longitudinally, and we therefore analyzed the longitudinal dataset using a mixed model (see Statistical analysis section). Inclusion and exclusion criteria, evaluation, clinical assessments, and fNIRS measurements were conducted at the University of Tokyo Hospital. This study was approved by the ethics committee of the Department of Medicine, The University of Tokyo [No. 630-(14), 2226-(12), and 3202-(11)]. Written informed consent was obtained from each study participant.

Exclusion criteria for all groups were as follows: (1) previous and/or present severe brain injury and/or neurological illness, (2) previous history of electroconvulsive therapy, (3) a premorbid IQ of 70 or less, (4) previous and/or present alcohol addiction, (5) previous and/or present continuous substance use, and (6) clear comorbidity with autism spectrum disorders.

### Demographic and clinical assessments

For all participants, we assessed handedness (Oldfield, 1971) and estimated their (premorbid) IQ using the 25-item version of the Japanese Adult Reading Test (Hirata-Mogi et al., 2016; Matsuoka & Kim, 2006; Matsuoka, Uno, Kasai, Koyama, & Kim, 2006). For all patient groups, we obtained symptom severity using the global assessment of functioning (GAF) scale, which is able to determine the severity of symptoms and functions across psychiatric disorders (American Psychiatric Association, 1994). We also assessed medication doses for antipsychotic (chlorpromazine), anticholinergic (biperiden), anxiolytic (diazepam), and antidepressant (imipramine) equivalent doses (Inada & Inagaki, 2015). For the schizophrenia and UHR groups, we assessed schizophrenia-related symptom severity using the Positive and Negative Syndrome Scale (PANSS) (Kay, Opler, & Fiszbein, 1991). For the BPD group, we assessed depressive symptom severity using the 17-item Hamilton Depression Rating Scale (HAM-D) scale (Hamilton, 1960) and manic symptoms using the Young Mania Rating Scale (YMRS) scale (Hamilton, 1960). For the MDD group, we assessed depressive symptoms using the HAM-D scale. The number of missing values for the demographic and clinical assessments is shown in online Supplementary Table S2.

### Brain function measurement

The same instrument (ETG-4000; Hitachi Ltd., Tokyo, Japan) and measurement procedures were used throughout the study period (Supplementary materials) (Koike et al., 2011, 2013a, 2016, 2017; Satomura et al., 2019; Takizawa et al., 2008, 2014). We used a 160-s block-designed phonological VFT that is well adapted as an activation task during fNIRS measurements (see Cognitive task section in Supplementary materials), and the number of words generated during the task period was assessed as task performance. After the measurement, we assessed subjective sleepiness during the task using the Stanford Sleepiness Scale (Hoddes, Zarcone, Smythe, Phillips, & Dement, 1973).

We used automatic rejection software revised from our previous study for visible artifacts derived from body and head movements (Supplementary materials) (Sakakibara et al., 2016). After treating fNIRS signals, we obtained two variables: brain activity and activity timing (Koike et al., 2017; Takizawa et al., 2014). Brain activity was defined as relative hemoglobin changes during the task period compared to pre- and post-task periods (nM⋅mm) (online Supplementary Fig. S1). Activity timing (C) is defined by the following formula:
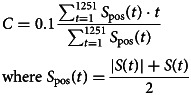
where *t* is time during the analyzed period (time resolution of 0.1 s) (see fNIRS signal treatment section in Supplementary materials). When we see the positive values of fNIRS signal as a frequency distribution graph, activity timing (*C*) is the ‘mean’ value of the frequency distribution graph throughout the analyzed period (*s*) (online Supplementary Fig. S1).

The location of fNIRS measurements for each channel was estimated using a probabilistic location by a virtual registration from MRI measurements with an fNIRS probe attachment (see Calculating fNIRS variables in the prefrontal and temporal cortices section in Supplementary materials) (Tsuzuki et al., 2007; Tsuzuki & Dan, 2014). The virtual registration for the 52-channel probe covered 12 brain regions in the front-temporal hemisphere using Automated Anatomical Labeling (AAL) (Tzourio-Mazoyer et al., 2002). To obtain reliable fNIRS signals in the brain regions within 25–35 cm of the T3-FPz-T4 segment according to the international 10–20 system used in electroencephalogram, we used eight regions per hemisphere for further analyses [superior frontal gyrus (SFG), superior frontal medial cortex (SFGM), middle frontal gyrus (MFG), inferior frontal gyrus triangularis (IFGTr), inferior frontal gyrus opercularis (IFGOp), inferior frontal gyrus orbital (IFGOr), superior temporal gyrus (STG), and middle temporal gyrus (MTG)].

### Statistical analysis

Overall statistical flow is shown in Fig. 1. We first compared the models to see the effects of demographic and clinical variables on fNIRS brain activity and activity timing using a GLMM (Fig. 1*a*–*d*). Then, we explored causal estimation related to brain activity using an SEM (Fig. 1*e*–*g*).

#### Model comparison

To test the effects of demographic variables on fNIRS brain activity and activity timing, we used a GLMM with the participant as a random effect of intercept and slope (Fig. 1*a* and Supplementary materials). We first compared the models to see the effects of demographic variables on fNIRS brain activity and activity timing in the control group. The model where all the coefficients were significant (two-tailed *p* < 0.05) and that had the smallest Akaike information criterion (AIC) was defined as the best-fitted model in each brain region (Fig. 1*b*). For multiple testing of the 16 brain regions, we applied a false discovery rate (FDR, *q* < 0.05) approach to increase the sensitivity and specificity of the analyses using neuroimaging variables that correlated with each other (Singh & Dan, 2006). All analyses were conducted using R version 3.5.1 (The R Foundation for Statistical Computing, Vienna, Austria), ‘lmer’, and ‘MuMIn’ packages (Bates, Machler, Bolker, & Walker, 2015; Burnham & Anderson, 2002; R Core Team, 2018). Then, we added other demographic variables and assessments of fNIRS measurements (handedness, IQ, task performance, and sleepiness) as independent variables to the best-fitted model and compared all possible models (Fig. 1*c*). We tested the main effect of diagnosis and diagnosis interaction by sex and age, as well as repeated fNIRS measurements in the same manner for all available data. We also added the effect of clinical assessment and medication equivalent dose on fNIRS variables for the patient groups controlling for demographic variables using the same model comparison method (see Cross disease comparisons and the effect of clinical variable section in Supplementary materials).

#### Causal estimation of brain activity

We applied the SEM to find the relationship with brain activity (Fig. 1*e*–*g*). Considering the correlations between demographic variables, symptom severity, and medication dose (online Supplementary Tables S3–6), we set a model in the schizophrenia, BPD, and MDD groups. We first determined the relationship irrespective of brain activity since the direction of the relationships was deterministic (Fig. 1*e*). After identifying the best model, we added brain activity for each fNIRS variable (Fig. 1*f*). As causality could be estimated for the relationships between fNIRS signals and symptom severity, and between fNIRS signals and task performance, we performed a model comparison in an SEM model to determine the relationship statistically (pink lines in Fig. 1*f*). One relationship had four possible paths: (1) no relationship, (2) path from task or symptom assessment to brain activity, (3) path from brain activity to the assessment, and (4) a correlation between the brain activity and the assessment (Fig. 1*g*). Therefore, we compared 16 models (4 × 4 models) for each fNIRS variable in each group. We applied the smallest AIC model to the best fit. SEM analyses were conducted using a ‘lavaan’ package within R software (Rosseel, 2012). The estimation of the model was conducted using a robust maximum likelihood estimation, and missing values were handled using a full information maximum likelihood method. Indices of a good-fit model were *p* values of 0.05 or greater from a χ^2^ test, a confirmatory fit index value of 0.90 or greater, or a root mean square error of approximation value of 0.10 or smaller.

## Results

### Effect of demographic variables on fNIRS data in the control group

**Fig. 2.**
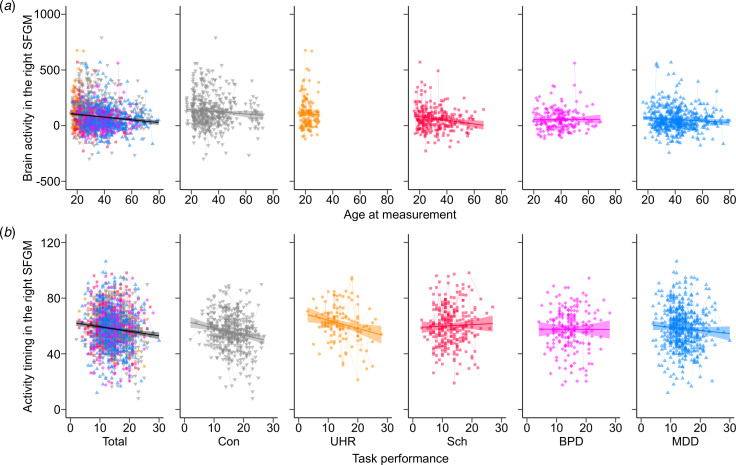
The correlation with functional near-infrared spectroscopy brain signals for each group. Correlations were shown in the total samples and the control (Con), ultra-high risk (UHR), schizophrenia (Sch), bipolar disorder (BPD), and major depressive disorder (MDD) groups (*a*) between the age at measurement (year) and brain activity (nM⋅mm) in the right superior frontal medial gyrus (SFGM), and (*b*) between task performance (number of words) and activity timing (s) in the right SFGM. A thick line and shaded area indicate the fix effect of the relationship in each group and the standard error of the slope. Thin lines show trajectories of repeated measurements for each participant.

A GLMM showed that male participants had greater brain activity during the task period compared to female participants in 12 brain regions over the PFC and right temporal cortex (FDR-corrected *p* < 0.05, online Supplementary Tables S7 and S8). Linear or quadratic age effects were found in brain activity of the bilateral STG, right IFGTr, and left MTG (Fig. 2*a*, and online Supplementary Tables S7 and S8). For activity timing across the task, a significant main effect of age was seen in the left STG. No cubic age effect or age and sex interaction was found.

The comparison adding the other demographic variables showed a significant positive effect of IQ on brain activity in the right MFG and IFGOr (online Supplementary Tables S7 and S9), and a negative effect of task performance on activity timing in the bilateral SFG, SFGM, and MFG, as well as the left IFGTr, IFGOp, and IFGOr (Fig. 2*b*, online Supplementary Table S10). There was a non-significant effect of handedness and sleepiness on brain activity and timing in all regions.

### Cross-disease comparisons

The difference in demographic, clinical, and fNIRS variables between patients with first-episode psychosis and chronic schizophrenia (online Supplementary Tables S11 and 12), and patients with type I and II BPD (online Supplementary Tables S13 and 14) are shown in Supplementary materials.

**Fig. 3.**
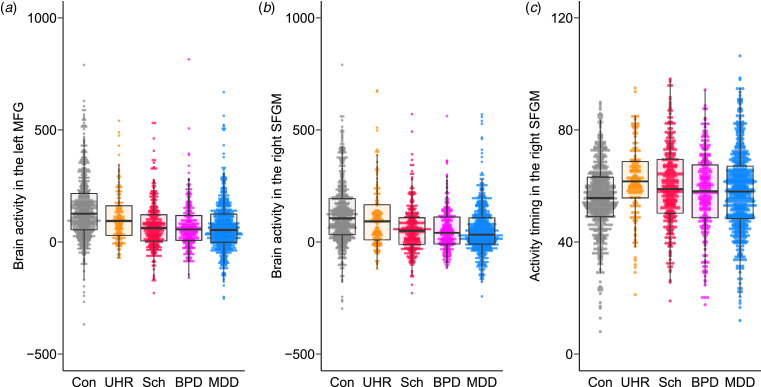
The differences in brain activity and activity timing between psychiatric disorders. Brain activity in (*a*) the left middle frontal gyrus (MFG) and (*b*) the right superior frontal medial gyrus (SFGM) and (*c*) activity timing in the right SFGM are plotted for the control (Con), ultra-high risk (UHR), schizophrenia (Sch), bipolar disorder (BPD), and major depressive disorder (MDD) groups. A box plot was overlaid.

A model comparison analysis showed that the patient groups had smaller brain activity in all regions compared to the controls (FDR-corrected *p* < 0.05; Fig. 3*a* and *b*, online Supplementary Fig. S2, and online Supplementary Tables S15 and S16). UHR individuals and patients with schizophrenia had more delayed activity timing in the bilateral SFGM and left MFG compared to controls (Fig. 3*c*). Activity timing in the right MFG and left SFG was more delayed in the UHR individuals compared to controls. No diagnosis interactions between sex and age were significant.

Within patient groups, UHR individuals had larger activity in the 10 regions of the bilateral PFC compared to the other three disease groups (online Supplementary Table S17). Activity timing in the right SFG was more delayed in the UHR group (FDR-corrected *p* = 0.0028). Activity timing in the bilateral SFG, SFGM, and MFG, as well as in the left IFGTr and IFGOr and right MTG was more delayed in the schizophrenia spectrum group (UHR and schizophrenia groups) (FDR-corrected *p* < 0.05).

### Effect of repeated fNIRS measurements on fNIRS signals

There was no main effect of either the intervals between measurements or intervals from the baseline measurement, nor any interaction by group for longitudinal measurements of brain activity or activity timing in any group (online Supplementary Fig. S3).

### Association between symptom severity and fNIRS signals

**Fig. 4.**
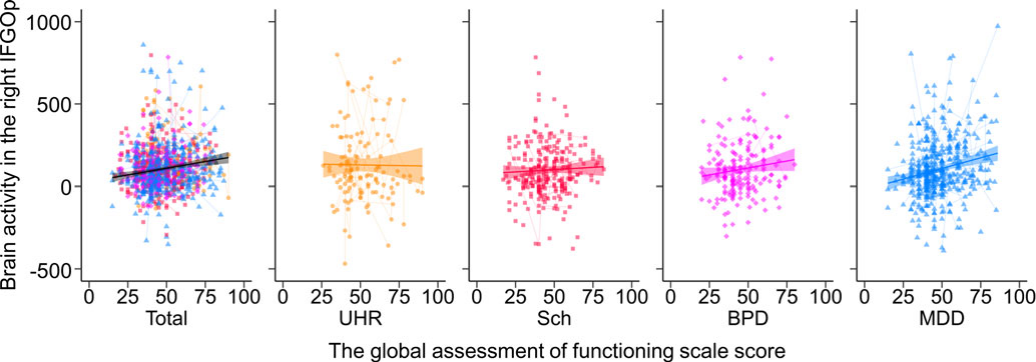
The association between brain activity and the global assessment of functioning score in the patient groups. The relationships between brain activity in the right inferior frontal gyrus opercularis (IFGOp) and the global assessment of functioning (GAF) score are plotted in the total, ultra-high risk (UHR), schizophrenia (Sch), bipolar disorder (BPD), and major depressive disorder (MDD) groups. Thin lines show trajectories of repeated measurements for each participant.

The GAF score in the patient group was positively associated with brain activity in the right IFGOp (Fig. 4) and left MTG, as well as activity timing in the right MTG (FDR-corrected *p* < 0.05, online Supplementary Tables S18 and S19). The UHR group showed a positive association between the PANSS-positive score and brain activity in the right STG, and the general psychopathology score in 14 regions (online Supplementary Fig. S4 and Table S20), while no other correlation with any clinical severity score was found in the other patient groups (Supplementary materials).

### Association between medication and fNIRS signals

A summary of the effect of medication doses on fNIRS signals in the patient group is shown in online Supplementary Table S18 and details are shown in Supplementary materials. Briefly, biperiden equivalent dose was negatively associated with brain activity in six regions (online Supplementary Table S21), especially in the schizophrenia group where the negative association was seen in all regions (online Supplementary Fig. S4 and Table S22). Diazepam equivalent dose was negatively associated with brain activity in 14 regions (online Supplementary Fig. S5 and Table S23). Imipramine equivalent dose was negatively associated with brain activity in the MDD group (online Supplementary Fig. S4 and Table S25) but not in the patient group (online Supplementary Table S24).

### Structural equation model for symptom severity, medication, and brain activity

**Fig. 5.**
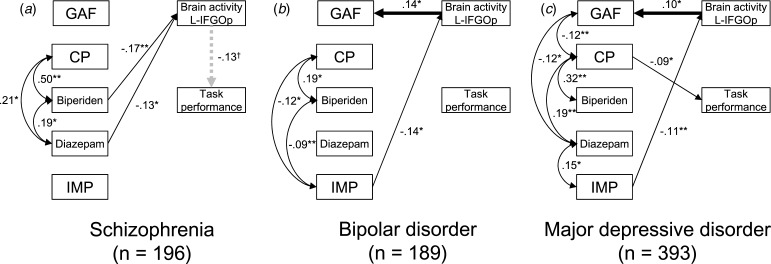
Structural equation models for symptom severity, medication, and brain activity in the schizophrenia, bipolar disorder, and major depressive disorder groups. The best fit models including brain activity in the left inferior frontal gyrus opercularis (L-IFGOp) are illustrated in (*a*) the schizophrenia, (*b*) bipolar disorder (BPD), and (*c*) major depressive disorder (MDD) groups (*p < 0.05, **p < 0.01). To illustrate the models simply, the paths from age, gender, and IQ were included but not shown. The model with all paths is shown in online Supplementary Figs. S6–8, respectively. The symbol † indicates the path from brain activity in the L-IFGOp to task performance was a trend (*p* = 0.088); although a model comparison showed this was the best model compared to the model including no association between them. GAF, global assessment of functioning; CP, chlorpromazine; IMP, imipramine.

Base models for three groups are shown in online Supplementary Figs. S6–S8. Based on these models, 16 possible models, including brain activity in each region for each group, were compared (online Supplementary Tables S26–S28). All models including the relationship between brain activity and GAF score had a path from brain activity to the GAF score. Of these, relationships from brain activity to the GAF score were seen in the left IFGOp for the BPD group (*β* = 0.136, s.e. = 0.061, *z* = 2.22, *p* = 0.027), and the bilateral STG (right: *β* = 0.112, s.e. = 0.054, *z* = 2.09, *p* = 0.037; left: *β* = 0.162, s.e. = 0.051, *z* = 3.20, *p* = 0.001) and MTG (right: *β* = 0.111, s.e. = 0.050, *z* = 2.21, *p* = 0.027; left: *β* = 0.168, s.e. = 0.045, *z* = 3.75, *p* < 0.001), and the left SFG (*β* = 0.100, s.e. = 0.049, *z* = 2.06, *p* = 0.039), IFGTr (*β* = 0.109, s.e. = 0.048, *z* = 2.27, *p* = 0.023), and IFGOp (*β* = 0.099, s.e. = 0.048, *z* = 2.05, *p* = 0.040) for the MDD group (Fig. 5). For the relationship between brain activity and task performance, the schizophrenia group had a path from brain activity to task performance in 10 regions while the MDD group had a path from task performance to brain activity in seven regions out of eight. The results of model comparisons for the other symptom severity scales are shown in online Supplementary Figs. S9–S14 and Tables S29–S34.

## Discussion

The present study investigated the relationship between the characteristics of hemoglobin changes during a phonological VFT using fNIRS and a wide range of demographic and clinical variables in a large sample size including multiple disease spectra. Our results showed that brain activity was affected by gender and age in a curvilinear model and decreased over the prefrontal and temporal cortical regions in the patient groups. However, activity timing was not affected by demographic variables, medication dose, or symptom severity, except for task performance. Within patients, the schizophrenia spectrum group (UHR and schizophrenia groups) had more delayed activity timing in the bilateral PFC. Considering the relationships between demographic variables (age, gender, and premorbid IQ) and medication dose in the SEMs, significant relationships from brain activity to GAF score were seen in the left IFGOp for the BPD group, and the bilateral STG and MTG and the left SFG, IFGTr, and IFGOp for the MDD group. To the best of our knowledge, this is the first study to find a disease-specific relationship between brain signals and global clinical severity in a large-scale sample considering the potential effect of demographic variables and medication doses.

Among group comparisons, the patient groups had a shared decrease of brain activity compared to the control group over the measurement area. Previous fNIRS studies repeatedly reported task-dependent activity over the prefrontal and temporal cortical area in healthy participants that decreased in patients with psychiatric disorders (Chou et al., 2015a, 2015b; Koike et al., 2011, 2016; Ohi et al., 2017; Takizawa et al., 2014). Comparable to studies comparing two or more disease groups (Ohi et al., 2017; Suto et al., 2004; Takizawa et al., 2014), the present findings showed similar decreases in brain activity in psychiatric diseases, suggesting shared functional deficits in the PFC. We showed little disease-specific differences in brain function; in fact, the variability within a group was greater than the differences between disease groups. This pattern has also been observed in a cross-disease comparison using resting-state functional MRI (Nakamura et al., 2020). Neuroimaging case–control studies have mostly relied on existing clinical categories that are considered to include the heterogeneity within a disease spectrum. In the future, neuroimaging-based classification may shed some light on new disease categories that may be more reliable in identifying brain pathologies.

The GLMMs showed that brain activity in the PFC is associated with diazepam equivalent dose in the patient group as well as biperiden in the schizophrenia group and imipramine in the MDD group. These results are in line with previous studies showing a negative correlation between brain activity and antidepressant dose in MDD (Noda et al., 2012; Takamiya et al., 2017; Tomioka et al., 2015), but not antipsychotic dose in schizophrenia (Koike et al., 2011, 2013a, 2016). Since the medication dose was greater in the schizophrenia, BPD, and MDD groups compared to the control and UHR, the reduced brain activity might be explained by medication. However, the coefficients for medication in the GLMMs and group differences in medication doses only partly explain the group differences in the GLMM. We previously showed that UHR individuals on medication had no difference in brain activity compared to those without (Koike et al., 2011). In addition, the prescription pattern was determined by diagnosis in a naturalistic study and the medication dose in their stable conditions reflects clinical severity. Thus, the effect of medication on brain activity may not simply figure out.

Brain activity was positively associated with global functioning using a comparison of SEMs while considering an association between demographic and clinical variables. These results are in line with previous investigations showing the negative association between brain activity in the right PFC and HAM-D depressive symptom (Fu et al., 2018) and the positive association with YMRS mania symptom in BPD (Nishimura et al., 2015). Further, previous studies have identified the negative association between brain activity and the HAM-D depression score in MDD (Noda et al., 2012; Satomura et al., 2019). Since our findings using the HAM-D and YMRS replicated the negative relationship between brain activity in the right IFGOr and depressive symptom in the BPD group, the fNIRS measurement during the VFT may be more useful in identifying the severity of depression in BPD. Although previous studies have observed an association between GAF score and brain activity in the bilateral SFG and SFGM (Koike et al., 2011, 2013a; Shimodera et al., 2012; Takizawa et al., 2008), the patients with schizophrenia in our SEMs had no relationship in any region based on the GAF or PANSS scores. This may be because the path from brain activity to the task performance, which was not seen in previous correlational analyses, might ameliorate the relationship between brain activity and symptom severity. Furthermore, we previously reported that patients with first-episode psychosis exhibited an association between brain activity and GAF scores at the 12-month follow-up measurement but not at baseline (Koike et al., 2016), suggesting that the patients with first-episode psychosis in the schizophrenia group in this study may have ameliorated the association.

Several limitations should be stated. First, although we tested the effect of medication in mixed model and SEM comparisons and found a correlation between brain activity and clinical symptoms while considering the effect of medication, a causal relationship should be confirmed using longitudinal measurements in a double-blinded placebo-controlled trial. In addition, we were unable to test drug-specific effects on brain activity in this naturalistic study. A previous study showed that antidepressant administration for drug-naïve patients with MDD had little effect on fNIRS brain activity; however, that study was unable to control the types of medication or use a placebo (Tomioka et al., 2015). Even in a naturalistic setting, it is possible to estimate the effect of specific drug administration (e.g. conventional antipsychotics, benzodiazepine, and lithium) on brain activity, which in future studies may help clarify the effect of a specific drug on cortical function. Second, since most of the previous neuroimaging studies measured brain activity for people in relatively stable conditions, it is unclear whether the findings were from the characteristics or course of the illness. We previously reported via a longitudinal measurement that fNIRS signals were relatively stable and did not change according to clinical severity in schizophrenia (Koike et al., 2016) and MDD (Satomura et al., 2019). However, the signals could be affected by aging; future studies should include longer follow-up periods.

Third, as discussed above, we did not observe a difference between the disease spectra, and the variability within a group and the overlap between groups was more pronounced compared with the differences between the disease groups. This limitation is still considered in psychiatric neuroimaging and genetic studies, and the development of more sophisticated modalities for the measurements of the human brain and neural activity, as well as multimodal investigations to combine and control the characteristics, is needed.

An analysis of this large and high-quality sample dataset demonstrated that alterations in prefrontal brain activity were a shared pathophysiology between schizophrenia, BPD, and MDD, while delayed activity timing was seen only in the schizophrenia spectrum. Even after controlling for various demographic and clinical variables, a relationship between brain activity in the PFC and symptom severity in the BPD and MDD groups was revealed. The results suggest what characteristics affect brain activity in the PFC and which set of demographic, clinical, and biological variables might predict symptom severity in patients with psychiatric disorders. Cross-disease mega neuroimaging studies with optimal and high-quality clinical data may offer new insights into finding common and disease-specific brain pathologies and lead to a brain function-based recategorization of disease spectra and treatment response.

## Supplementary material

For supplementary material accompanying this paper visit https://doi.org/10.1017/S0033291720004742. click here to view supplementary material
