# Supplementary material for: Shared functional impairment in the prefrontal cortex affects symptom severity across psychiatric disorders
Source: Psychol Med. 2020 Dec 18;52(13):2661–70. doi: 10.1017/S0033291720004742 (PMC9647535; doi:10.1017/S0033291720004742)
Supplement: Supplementary file 1 [file S0033291720004742sup.zip › S0033291720004742sup002.docx]

**Shared functional impairment in the prefrontal cortex affects symptom severity across psychiatric disorders**

Shinsuke Koike, M.D., Ph.D.; Eisuke Sakakibara, M.D., Ph.D.; Yoshihiro Satomura, M.D., Ph.D.; Hanako Sakurada, MSc.; Mika Yamagishi, MSc.; Jun Matsuoka, M.D., Ph.D.; Naohiro Okada, M.D., Ph.D.; Kiyoto Kasai, M.D., Ph.D.

**Supplementary Materials**

[Methods of diagnosis 5](#_Toc44938995)

[Criteria for the UHR group 5](#_Toc44938996)

[Brain function measurement using an fNIRS instrument 8](#_Toc44938997)

[*fNIRS instrument* 8](#_Toc44938998)

[*Cognitive task* 8](#_Toc44938999)

[*fNIRS signal treatment* 9](#_Toc44939000)

[*Artifact rejection software contained in functional near-infrared spectroscopy (fNIRS) signals* 10](#_Toc44939001)

[*Calculating fNIRS variables in the prefrontal and temporal cortices* 12](#_Toc44939002)

[Statistical analysis 15](#_Toc44939003)

[*Model comparison* 15](#_Toc44939004)

[*Cross disease comparisons and the effect of clinical variables* 16](#_Toc44939005)

[*Exploring the effects of demographics, symptom severity, and medication on brain activity with the structural equation model* 17](#_Toc44939006)

[Difference in demographic, clinical, and fNIRS variables between sub-groups 20](#_Toc44939007)

[Association between other clinical variables and fNIRS signals 20](#_Toc44939008)

[Association between medication and fNIRS signals 21](#_Toc44939009)

[Structural equation model for symptom severity, medication, and brain activity including the other severity scales 22](#_Toc44939010)

[References for supplementary materials 23](#_Toc44939011)

[Supplementary Figures 26](#_Toc44939012)

[Supplementary Figure S1. Functional near-infrared spectroscopy (fNIRS) variables in this study. 26](#_Toc44939013)

[Supplementary Figure S2. The difference in brain activity over the fNIRS brain regions. 27](#_Toc44939014)

[Supplementary Figure S3. The effect of repeated measurements on functional near-infrared spectroscopy brain signals. 28](#_Toc44939015)

[Supplementary Figure S4. The association between clinical variables and brain activity. 29](#_Toc44939016)

[Supplementary Figure S5. The association between brain activity in the left inferior frontal gyrus triangularis and medication equivalent dose in the patient groups. 30](#_Toc44939017)

[Supplementary Figure S6. The base structural equation model including the GAF scale in the schizophrenia group. 31](#_Toc44939018)

[Supplementary Figure S7. The base structural equation model including the GAF scale in the bipolar disorder group. 32](#_Toc44939019)

[Supplementary Figure S8. Base structural equation model including the GAF scale in the major depressive disorder group. 33](#_Toc44939020)

[Supplementary Figure S9. The base structural equation model including the PANSS positive subscale in the schizophrenia group. 34](#_Toc44939021)

[Supplementary Figure S10. The base structural equation model including the PANSS negative subscale in the schizophrenia group. 35](#_Toc44939022)

[Supplementary Figure S11. The base structural equation model including the PANSS general psychopathology subscale in the schizophrenia group. 36](#_Toc44939023)

[Supplementary Figure S12. The base structural equation model including the YMRS scale in the bipolar disorder group. 37](#_Toc44939024)

[Supplementary Figure S13. The base structural equation model including the HAM-D scale in the bipolar disorder group. 38](#_Toc44939025)

[Supplementary Figure S14. Base structural equation model including the HAM-D scale in the major depressive disorder group. 39](#_Toc44939026)

[Supplementary Tables 40](#_Toc44939027)

[Supplementary Table S1. Demographic characteristics in this study. 40](#_Toc44939028)

[Supplementary Table S2. The number of missing values 42](#_Toc44939029)

[Supplementary Table S3. The correlation matrix for the combined patient groups 43](#_Toc44939030)

[Supplementary Table S4. The correlation matrix for the schizophrenia group 44](#_Toc44939031)

[Supplementary Table S5. The correlation matrix for the bipolar disorder group 45](#_Toc44939032)

[Supplementary Table S6. The correlation matrix for the major depressive disorder group 46](#_Toc44939033)

[Supplementary Table S7. Summary of the effect of demographics on fNIRS signals in the control group 47](#_Toc44939034)

[Supplementary Table S8. The effect of age and sex on fNIRS signals in the control group 48](#_Toc44939035)

[Supplementary Table S9. The effect of estimated IQ on fNIRS signals in the control group 50](#_Toc44939036)

[Supplementary Table S10. The effect of task performance on fNIRS signals in the control group 52](#_Toc44939037)

[Supplementary Table S11. Differences in demographics between patients with first-episode psychosis and chronic schizophrenia 54](#_Toc44939038)

[Supplementary Table S12. Differences in fNIRS variables between patients with first-episode psychosis and chronic schizophrenia 55](#_Toc44939039)

[Supplementary Table S13. Differences in demographics between patients with type I and II bipolar disorders 57](#_Toc44939040)

[Supplementary Table S14. Differences in fNIRS variables between patients with type I and II bipolar disorder 58](#_Toc44939041)

[Supplementary Table S15. The effect of diagnosis and age interaction on fNIRS signals 60](#_Toc44939042)

[Supplementary Table S16. The effect of diagnosis on fNIRS signals 62](#_Toc44939043)

[Supplementary Table S17. The effect of diagnosis on fNIRS signals in the patient group 64](#_Toc44939044)

[Supplementary Table S18. Summary of the effect of symptom severity and medication doses on fNIRS signals in the patient group. 66](#_Toc44939045)

[Supplementary Table S19. The effect of the GAF score on fNIRS signals in the patient group 67](#_Toc44939046)

[Supplementary Table S20. The effect of the PANSS general psychopathology on fNIRS signals in the UHR group 69](#_Toc44939047)

[Supplementary Table S21. The effect of biperiden equivalent dose on fNIRS signals in the patient group 71](#_Toc44939048)

[Supplementary Table S22. The effect of biperiden equivalent dose on fNIRS signals in the schizophrenia group 73](#_Toc44939049)

[Supplementary Table S23. The effect of diazepam equivalent dose on fNIRS signals in the patient group 75](#_Toc44939050)

[Supplementary Table S24. The effect of imipramine equivalent dose on fNIRS signals in the patient group 77](#_Toc44939051)

[Supplementary Table S25. The effect of imipramine equivalent dose on fNIRS signals in the MDD group 79](#_Toc44939052)

[Supplementary Table S26. A list of the best fit models including the GAF scale in the SEM model comparison of the schizophrenia group 81](#_Toc44939053)

[Supplementary Table S27. A list of the best fit models including the GAF scale in the SEM model comparison of the BPD group 82](#_Toc44939054)

[Supplementary Table S28. A list of the best fit models including the GAF scale in the SEM model comparison of the MDD group 83](#_Toc44939055)

[Supplementary Table S29. A list of the best fit models including the PANSS positive subscale in the SEM model comparison of the schizophrenia group 84](#_Toc44939056)

[Supplementary Table S30. A list of the best fit models including the PANSS negative subscale in the SEM model comparison of the schizophrenia group 86](#_Toc44939057)

[Supplementary Table S31. A list of the best fit models including the PANSS general psychopathology subscale in the SEM model comparison of the schizophrenia group 88](#_Toc44939058)

[Supplementary Table S32. A list of the best fit models including the YMRS scale in the SEM model comparison of the BPD group 90](#_Toc44939059)

[Supplementary Table S33. A list of the best fit models including the HAM-D scale in the SEM model comparison of the BPD group 91](#_Toc44939060)

[Supplementary Table S34. A list of the best fit models including the HAM-D scale in the SEM model comparison of the MDD group 92](#_Toc44939061)

# **Methods of diagnosis**

The diagnoses of the participants were obtained by 2 or more trained research psychiatrists through detailed clinical interviews before their fNIRS measurements, 2 or more trained clinical psychiatrists during admission, and/or using the Structured Clinical Interview for Diagnostic and Statistical Manual of Mental Disorders Version 4 (DSM-IV) Axis I Disorders by trained psychologists. Any inconsistencies in the diagnoses across the methods were not included in this analysis. The definition of UHR was conducted using the Japanese version of the Structured Interview for Prodromal Symptoms (Supplementary materials) (Kobayashi, Nozaki, & Mizuno, 2007; Miller et al., 1999). We screened healthy controls using the modified Mini-International Neuropsychiatric Interview (Otsubo et al., 2005; Sheehan et al., 1998) or the World Health Organization Composite International Diagnostic Interview (Kawakami et al., 2005; Kessler & Ustun, 2004). We also excluded potential controls with a family history of any axis I disorder(s) in a first-degree relative.

# **Criteria for the UHR group**

The inclusion criteria for the ultra-high risk for psychosis (UHR) group were defined using the Structured Interview for Prodromal Syndromes (SIPS) criteria by assessing the Scale of Prodromal Symptoms (SOPS) (Kobayashi et al., 2007; Miller et al., 1999). The SIPS criteria contain 3 conditions: 1) Attenuated Positive Symptom Syndrome (APSS), 2) Brief Intermittent Psychotic Symptom Syndrome (BIPS), and 3) Genetic Risk and Deterioration Syndrome (GRDS). The criteria of each condition are as follows:

1) APSS

Satisfying all the following items (a–c):

a) The presence of at least one of the following 5 SOPS positive items in the prodromal range (rating of 3–5): P1 (Unusual Thought Content/Delusional Ideas), P2 (Suspiciousness/Persecutory Ideas), P3 (Grandiosity), P4 (Perceptual Abnormalities/Hallucinations), and/or P5 (Disorganized Communication).

b) Symptoms began within the past year or increased by 1 or more points in the SOPS assessment within the past year.

c) Symptoms occurred at least once per week for the last month.

2) BIPS

Satisfying all the following items (a–c):

a) The presence of at least 1 of 5 SOPS positive items in the psychotic range (rating of 6).

b) Symptoms began in the past 3 months.

c) Symptoms occurred at least several minutes per day and at least once per month.

3) GRDS

Satisfying all the following items (a–b):

a) A first degree relative had a history of a psychotic disorder, or criteria for schizotypal personality disorder were met in the patient.

b) The global assessment of functioning (GAF) score dropped at least 30% over the last month compared to 1 year ago.

# **Brain function measurement using an fNIRS instrument**

## *fNIRS instrument*

A 52-channel fNIRS instrument (ETG-4000; Hitachi Ltd., Tokyo, Japan) was used to measure the relative changes in oxygenated hemoglobin concentration that reﬂect cortical activity. The fNIRS probe attachment was set with 33 probes in a thermo-plastic 3 × 11 shell to cover the bilateral prefrontal and anterior temporal cortices, with the lowest probe line set along the Fp1–Fp2 line deﬁned by the international 10–20 system commonly used in electroencephalography. Participants only needed to sit in a chair in a relaxed state with their eyes open. To minimize motion artifacts, we instructed them to refrain from physical movements such as head motions and strong biting during measurements.

## *Cognitive task*

We used a 160-s block-designed phonological verbal fluency task that is well adapted as an activation task during fNIRS measurements (Koike, Nishimura, Takizawa, Yahata, & Kasai, 2013; Koike et al., 2017; Koike et al., 2016; Koike et al., 2011; Satomura et al., 2019; Takizawa et al., 2014; Takizawa et al., 2008). The task consists of 30-s pre-task, 60-s task, and 70-s post-task periods. In the 60-s task period, the participant was instructed to say as many words aloud as possible that start with a phonological syllable provided by a computer. The task period was divided into 3 sub-periods, and the instructed syllables changed every 20 s to avoid silent moments. In the 30-s pre-task and 70-s post-task periods, the participant was instructed to say Japanese vowels aloud repeatedly, to control for task-related motion artifacts and facilitate their removal. This procedure can spatio-temporally measure hemoglobin changes, mainly in the prefrontal cortex and the anterior and superior parts of the temporal cortex, because continuous word generation exercises various cognitive domains involved in verbal storage, verbal working memory, inhibition, and executive control to avoid repetition and inappropriate word use. The number of words generated during the task period was assessed as task performance. We also assessed subjective sleepiness during the task using the Stanford Sleepiness Scale (Hoddes, Zarcone, Smythe, Phillips, & Dement, 1973) after the measurement.

## *fNIRS signal treatment*

Signals for fNIRS oxygenated hemoglobin were acquired for 0.1 s. Then, the signals were focused on task-specific signal changes using a linear fitting between the last 10 s of the pre-task period and the 5 s between the 50- and 55-s time points of the post-task period.

For visible artifacts derived from body and head movements, we used automatic rejection software revised from our previous study described in detail below (Sakakibara et al., 2016). We classified the artifacts contained in fNIRS signals into the following 3 types: flattened signals, Gaussian noise, and motion artifacts (described in supplementary materials). We discarded the entire signal from a channel when it contained artifacts exceeding the predetermined threshold. Therefore, the available channels were different for each participant. If 27 or more channels were rejected, we excluded the measurement itself from the analysis. Finally, fNIRS oxygenated hemoglobin signals were smoothed for 5 s to clear high frequency noises such as heartbeat and small motion artifacts.

## *Artifact rejection software contained in functional near-infrared spectroscopy (fNIRS) signals*

We classified the following 3 types of artifacts and discarded the entire signal from a channel when it contained 1 or more types of artifacts exceeding the threshold.

1) Removal of flattened signals

Flattened signals were observed when a connection problem occurred or when signals went off the scale. A linear transformation was applied to raw signals; the flat signal was registered as an arithmetic sequence of signal values (e.g., 0, 0.1, 0.2, 0.3, 0.4…). Using the “diff” function twice in MATLAB version 2015b (The MathWorks, Inc., MA, U.S.A.), we judged a signal as flattened when the second order difference sequence of the signal value was almost 0 for more than 3 consecutive time points. Then, we excluded the data that contained flattened signals as more than 1% the measurement.

2) Removal of noisy signals

Gaussian noise reflects defective probe contact with the skin. We used a Butterworth high-pass filter (0.08 Hz or more) and performed zero-phase digital filtering of deoxy-Hb and total-Hb (i.e., deoxy-Hb + oxy-Hb) signals by processing the data in both the forward and backward directions in MATLAB. Then, we calculated the power of high-pass deoxy-Hb signals to estimate the amount of noise. In addition, we also calculated the ratio of the power of high-pass deoxy-Hb signals to the power of total-Hb signals as an indicator of the inverse of signal-to-noise ratio. We decided to exclude signals either when the power of the high-pass deoxy-Hb signal was larger than 0.5 in arbitrary units, or when the ratio of the power of the high-pass deoxy-Hb signals to the power of total-Hb signals exceeded 2.

3) Removal of data with motion artifacts

When the probes placed on the skin surface slip during measurement, fNIRS signals sometimes register sudden and abrupt baseline shifts. This type of artifact causes a substantial change in the Integral and Centroid values of the signals, especially when the signal baseline shifts and does not recover.

We first detected abrupt changes in fNIRS signals using the “findpeaks” function in MATLAB. A change was considered abnormal when the magnitude of the change was greater than 5 standard deviations in the distribution of signal value changes using intervals of 0.5 sec. Then, we derived 2 first regression lines using signal data before and after the point of abrupt change and calculated the magnitude of unrecovered baseline shifts from the intercept differences. Finally, we excluded data when the maximum magnitude of an unrecovered baseline shift was greater than a predetermined threshold (0.45 in arbitrary units).

## *Calculating fNIRS variables in the prefrontal and temporal cortices*

From the fNIRS signals, we obtained 2 fNIRS variables: brain activity and activity timing (Koike et al., 2017; Takizawa et al., 2014). Brain activity is defined as relative hemoglobin changes during the task period compared to pre- and post-task periods (nM·mm), also called the Integral value in the fNIRS application system (Supplementary Figure S1). Activity timing (C) is defined by the following formula:


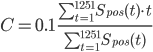


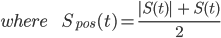


Where *t* is time during the analyzed period (time resolution of 0.1 s), after 10 s before starting the task period. When we see the positive values of fNIRS signal as a frequency distribution graph, activity timing (C) is the "mean" value of the frequency distribution graph between 10 s before starting the task period and 55 s of the post-task period (1250 time intervals), called the Centroid value (s).

The location of fNIRS measurements for each channel was estimated using a probabilistic location by a virtual registration from MRI measurements with an fNIRS probe attachment (Tsuzuki & Dan, 2013; Tsuzuki et al., 2007). This method provides the probability (p) of a brain region for each channel (ch) within 1 cm of the T3–T4 segment. Using this registration, we estimated brain signals in each brain region of interest (ROI) using the formula:

Signal_T3T4,ROI_ = (Σ p_ch_ × Signal_ch_) / p_sum_.

The virtual registration for the 52-channel probe covered 12 brain regions in the front-temporal hemisphere using Automated Anatomical Labeling (AAL) (Tzourio-Mazoyer et al., 2002). To obtain reliable fNIRS signals during the verbal fluency task in the brain regions within 25–35 cm of the T3–T4 segment, we used 8 regions per hemisphere for further analyses (SFG, SFGM, MFG, IFGTr, IFGOp, IFGOr, STG, and MTG). Missing values for the T3–T4 segment were substituted by the mean length for male and female participants in this study, 31 cm for male participants and 29 cm for female, respectively.

# **Statistical analysis**

## *Model comparison*

We tested the effects of demographic variables on fNIRS brain activity and activity timing in the control group using a GLMM with the participant as a random effect of intercept and slope. In this model, we tested the effect of demographic variables and repeated measurements simultaneously. First, we explored main effect of sex and age, age interaction by sex, and a non-parametric age effect using quadratic and cubic models. Then, we conducted a model comparison between all possible regression models using the variables for each brain signal. In the initial model shown in Fig. 1a, if the saturated model contained 5 variables (sex, age, age^2^, age^3^, and sex × age interaction), 32 (2^5^) possible models were compared in this analysis. Since demographic variables potentially include sampling bias across the patient groups, the effect of demographic variables was tested only in the control group. The model where all the coefficients were significant (*2-tailed p* < .05) and that had the smallest Akaike information criterion (AIC) was defined as the best fitted model in each brain region (Fig. 1b). In this example, the model with the smallest AIC included main effects of age (β_1_) and sex (β_4_) and sex × age interaction (β_5_), but β_1_ or β_5_ were not significant. Therefore, the second smallest AIC model was applied. For multiple testing of the 16 brain regions, we applied an FDR (*q* < .05) to the analyses (Singh & Dan, 2006). All analyses were conducted using R version 3.5.1 (The R Foundation for Statistical Computing, Vienna, Austria), “lmer” and “MuMIn” packages (Bates, Machler, Bolker, & Walker, 2015; Burnham & Anderson, 2002; R Core Team, 2018). Then, we added other demographic variables and assessments of fNIRS measurements one by one (handedness, IQ, task performance, and sleepiness) as independent variables to the best-fitted model and compared all possible models (Fig. 1c).

## *Cross disease comparisons and the effect of clinical variables*

Before cross-disease investigations, we tested the difference in demographic and clinical variables, and fNIRS signals between patients with first-episode psychosis and chronic schizophrenia, and between BPD type I and II, for further analysis. If no significant differences were present, we combined the patients into schizophrenia and BPD groups, respectively.

First, we used the best-fitted model in the control groups and added the main effect of diagnosis and diagnosis interaction by sex, then age for the model comparison (Fig. 1c). Second, we tested whether a disease-specific progression could be seen from the longitudinal fNIRS measurements. Third, we tested the effect of a clinical assessment (GAF) and medication equivalent dose on fNIRS variables for the patient group. Variables for specific symptom severity (PANSS, HAM-D, and YMRS) were also tested for the schizophrenia and UHR, BPD and MDD, and BPD groups, respectively.

## *Exploring the effects of demographics, symptom severity, and medication on brain activity with the structural equation model*

Since demographic variables, symptom severity, and medication doses were correlated (Supplementary Tables S3-6), we applied the SEM to find variables associated with brain activity (Fig. 1 d-g).

First, we used fNIRS-independent variables in the schizophrenia, BPD, and MDD groups since the sample size was large enough for 10 demographic and clinical variables in the SEM. Considering the correlations between demographic variables, symptom severity, and medication dose, we set a base model in the schizophrenia, BPD, and MDD groups (Fig. 1e), then explored the relationship with fNIRS signals (Fig. 1f). The base model included the relationship of demographic variables (sex, age, and IQ) with symptom severity, medication dose (chlorpromazine, biperiden, diazepam, and imipramine equivalent doses), and task performance. Since we were unable to see any causality between symptom severity and medication dose in the dataset, we set correlations between them. Also, since the base model did not include any paths from/to fNIRS signals, we did not exclude participants who had 27 or more channels of artifact-rejected fNIRS signals (see fNIRS signal treatment section). Thus, the analyzed sample size was 204 patients with schizophrenia, 191 with BPD, and 397 with MDD. We optimized the base model to exclude non-significant paths from a saturated model for each group.

Next, we added paths between these variables and fNIRS signals to the optimized model and analyzed each group in each brain region (Fig. 1f). We set paths from the medication dose to fNIRS variables and task performance. As causality could be estimated for the relationships between fNIRS signals and symptom severity, and between fNIRS signals and task performance, we performed a model comparison in an SEM model to determine the relationship statistically (pink lines in Fig. 1f). One relationship had 4 possible paths: (1) no relationship, (2) path from task or symptom assessment to brain activity, (3) path from brain activity to the assessment, and (4) a correlation between the brain activity and the assessment (Fig. 1g). Therefore, we compared 16 models (4 × 4 models) for each fNIRS variable in each group. We applied the smallest AIC model to the best fit. SEM analyses were conducted using a “lavaan” package within R software (Rosseel, 2012). The estimation of the model was conducted using a robust maximum likelihood estimation, and missing values were handled using a full information maximum likelihood method. Indices of a good-fit model were *p* values of .05 or greater from a chi-square test, a CFI value of 0.90 or greater, or an RMSEA value of 0.10 or smaller.

# **Difference** **in demographic, clinical, and fNIRS variables between sub-groups**

Patients with first-episode psychosis were younger (*p* < .001) and had fewer imipramine equivalent doses (*p* = .002; Supplementary Table S11) compared to patients with chronic schizophrenia; however, no significant differences were found in fNIRS variables (*p* >.05, Supplementary Table S12). Patients with type II BPD exhibited greater task performance compared to those with type I BPD (*p* = .040; Supplementary Table S13) but no significant differences were found in other demographic and fNIRS variables (*p* >.05, Supplementary Table S14).

# **Association between other clinical variables and fNIRS signals**

In the UHR and schizophrenia groups, there was no main effect of symptom score, but there was the interaction by group for the PANSS positive and general psychopathology subscales in the 11 and 9 brain regions, respectively (*FDR-corrected p* < .05). In the UHR group, the PANSS positive score was positively associated with brain activity in the right STG (*B* = 12.8, *SE* = 3.97, *FDR-corrected p* = .0016) and was positively associated with the general psychopathology score in 14 regions (Supplementary Figure S4a and Table S20). For the depressive and manic symptom scales, there was no significant association (Supplementary Table S18).

# **Association between medication and fNIRS signals**

The model comparison analysis showed that the chlorpromazine equivalent dose was negatively associated with brain activity in the right STG and left IFGTr (*FDR-corrected p* = .0028 and .0024, respectively; Supplementary Figure S5a). The biperiden equivalent dose was negatively associated with brain activity in 6 regions and activity timing in the right MTG (Supplementary Table S21). Interactions among the UHR and schizophrenia groups were also significant for brain activity in 7 and 6 regions of the bilateral prefrontal cortex, respectively. Interaction with schizophrenia for activity timing in the right MTG was also significant. Post-hoc analysis showed that only the schizophrenia group had a negative association between biperiden dose and brain activity in all regions (Supplementary Figure S4b and Table S22). For activity timing, only the MDD group had a negative association in the right MTG (*B* = -3.32, *FDR-corrected p* = .0016). The diazepam equivalent dose was negatively associated with brain activity in 14 regions of the measurement area and there was no interaction by group (Supplementary Figure S5b and Table S23). There was no main effect of imipramine equivalent dose on the fNIRS variables; however, a schizophrenia group interaction was found for brain activity in all regions except for the right MTG (Supplementary Table S24). Post-hoc analysis showed the MDD group had a negative association of brain activity in 13 regions (Supplementary Figure S4c and Table S25).

# **Structural equation model for symptom severity, medication, and brain activity including the other severity scales**

Base models including the PANSS, YMRS, and HAM-D scales for the corresponding patient groups are shown in Supplementary Figures S9-S14. Based on these models, the 16 possible models including brain activity in each region for each group were compared (Supplementary Tables S29-S34). In the schizophrenia group, relationships from brain activity to the PANSS positive score were seen in the left MTG (*β* = -0.137, *SE* = 0.062, *z* = -2.22, *p* = 0.026) and to the PANSS negative score in the right MTG (*β* = -0.122, *SE* = 0.061, *z* = -2.00, *p* = 0.046). In the BPD group, relationships from brain activity in the right IFGOr to the HAM-D score were seen (*β* = -0.005, *SE* = 0.003, *z* = -1.99, *p* = 0.046). There was no relationship between brain activity in any region and the HAM-D score in the MDD group (p > 0.05).

# **References for supplementary materials**

Bates, D., Machler, M., Bolker, B. M., & Walker, S. C. (2015). Fitting linear mixed-effects models using lme4. *Journal of Statistical Software, 67*(1), 1-48. doi:10.18637/jss.v067.i01

Burnham, K. P., & Anderson, D. R. (2002). *Model Selection and Multimodel Inference*. NY, USA: Springer-Verlag New York.

Hoddes, E., Zarcone, V., Smythe, H., Phillips, R., & Dement, W. C. (1973). Quantification of sleepiness: a new approach. *Psychophysiology, 10*(4), 431-436. doi:10.1111/j.1469-8986.1973.tb00801.x

Kawakami, N., Takeshima, T., Ono, Y., Uda, H., Hata, Y., Nakane, Y., . . . Kikkawa, T. (2005). Twelve-month prevalence, severity, and treatment of common mental disorders in communities in Japan: preliminary finding from the World Mental Health Japan Survey 2002-2003. *Psychiatry and Clinical Neurosciences, 59*(4), 441-452. doi:10.1111/j.1440-1819.2005.01397.x

Kessler, R. C., & Ustun, T. B. (2004). The World Mental Health (WMH) survey initiative version of the World Health Organization (WHO) Composite International Diagnostic Interview (CIDI). *International Journal of Methods in Psychiatric Research, 13*(2), 93-121. Retrieved from https://www.ncbi.nlm.nih.gov/pubmed/15297906

Kobayashi, H., Nozaki, S., & Mizuno, M. (2007). Reliability of the structured interview for prodromal syndromes Japanese version (SIPS-J). *Japanese Bulletin of Social Psychiatry, 15*(2), 168-174.

Koike, S., Nishimura, Y., Takizawa, R., Yahata, N., & Kasai, K. (2013). Near-infrared spectroscopy in schizophrenia: a possible biomarker for predicting clinical outcome and treatment response. *Frontiers in Psychiatry, 4*, 145. doi:10.3389/fpsyt.2013.00145

Koike, S., Satomura, Y., Kawasaki, S., Nishimura, Y., Kinoshita, A., Sakurada, H., . . . Kasai, K. (2017). Application of functional near infrared spectroscopy as supplementary examination for diagnosis of clinical stages of psychosis spectrum. *Psychiatry and Clinical Neurosciences, 71*(12), 794-806. doi:10.1111/pcn.12551

Koike, S., Satomura, Y., Kawasaki, S., Nishimura, Y., Takano, Y., Iwashiro, N., . . . Kasai, K. (2016). Association between rostral prefrontal cortical activity and functional outcome in first-episode psychosis: a longitudinal functional near-infrared spectroscopy study. *Schizophrenia Research, 170*(2-3), 304-310. doi:10.1016/j.schres.2016.01.003

Koike, S., Takizawa, R., Nishimura, Y., Takano, Y., Takayanagi, Y., Kinou, M., . . . Kasai, K. (2011). Different hemodynamic response patterns in the prefrontal cortical sub-regions according to the clinical stages of psychosis. *Schizophrenia Research, 132*(1), 54-61. doi:10.1016/j.schres.2011.07.014

Miller, T. J., McGlashan, T. H., Woods, S. W., Stein, K., Driesen, N., Corcoran, C. M., . . . Davidson, L. (1999). Symptom assessment in schizophrenic prodromal states. *Psychiatric Quarterly, 70*(4), 273-287. doi:10.1023/a:1022034115078

Otsubo, T., Tanaka, K., Koda, R., Shinoda, J., Sano, N., Tanaka, S., . . . Kamijima, K. (2005). Reliability and validity of Japanese version of the Mini-International Neuropsychiatric Interview. *Psychiatry and Clinical Neurosciences, 59*(5), 517-526. doi:10.1111/j.1440-1819.2005.01408.x

R Core Team. (2018). R: A language and environment for statistical computing. R Foundation for Statistical Computing. Retrieved from http://www.R-project.org/

Rosseel, Y. (2012). lavaan: An R package for structural equation modeling. *Journal of Statistical Software, 48*(2), 1-36. doi:10.18637/jss.v048.i02

Sakakibara, E., Homae, F., Kawasaki, S., Nishimura, Y., Takizawa, R., Koike, S., . . . Kasai, K. (2016). Detection of resting state functional connectivity using partial correlation analysis: A study using multi-distance and whole-head probe near-infrared spectroscopy. *Neuroimage, 142*, 590-601. doi:10.1016/j.neuroimage.2016.08.011

Satomura, Y., Sakakibara, E., Takizawa, R., Koike, S., Nishimura, Y., Sakurada, H., . . . Kasai, K. (2019). Severity-dependent and -independent brain regions of major depressive disorder: A long-term longitudinal near-infrared spectroscopy study. *Journal of Affective Disorders, 243*, 249-254. doi:10.1016/j.jad.2018.09.029

Sheehan, D. V., Lecrubier, Y., Sheehan, K. H., Amorim, P., Janavs, J., Weiller, E., . . . Dunbar, G. C. (1998). The Mini-International Neuropsychiatric Interview (M.I.N.I.): the development and validation of a structured diagnostic psychiatric interview for DSM-IV and ICD-10. *Journal of Clinical Psychiatry, 59 Suppl 20*, 22-33;quiz 34-57. Retrieved from https://www.ncbi.nlm.nih.gov/pubmed/9881538

Singh, A. K., & Dan, I. (2006). Exploring the false discovery rate in multichannel NIRS. *Neuroimage, 33*(2), 542-549. doi:10.1016/j.neuroimage.2006.06.047

Takizawa, R., Fukuda, M., Kawasaki, S., Kasai, K., Mimura, M., Pu, S., . . . Joint Project for Psychiatric Application of Near-Infrared Spectroscopy, G. (2014). Neuroimaging-aided differential diagnosis of the depressive state. *Neuroimage, 85 Pt 1*, 498-507. doi:10.1016/j.neuroimage.2013.05.126

Takizawa, R., Kasai, K., Kawakubo, Y., Marumo, K., Kawasaki, S., Yamasue, H., & Fukuda, M. (2008). Reduced frontopolar activation during verbal fluency task in schizophrenia: a multi-channel near-infrared spectroscopy study. *Schizophrenia Research, 99*(1-3), 250-262. doi:10.1016/j.schres.2007.10.025

Tsuzuki, D., & Dan, I. (2014). Spatial registration for functional near-infrared spectroscopy: From channel position on the scalp to cortical location in individual and group analyses. *Neuroimage*. doi:10.1016/j.neuroimage.2013.07.025

Tsuzuki, D., Jurcak, V., Singh, A. K., Okamoto, M., Watanabe, E., & Dan, I. (2007). Virtual spatial registration of stand-alone fNIRS data to MNI space. *Neuroimage, 34*(4), 1506-1518. doi:10.1016/j.neuroimage.2006.10.043

Tzourio-Mazoyer, N., Landeau, B., Papathanassiou, D., Crivello, F., Etard, O., Delcroix, N., . . . Joliot, M. (2002). Automated anatomical labeling of activations in SPM using a macroscopic anatomical parcellation of the MNI MRI single-subject brain. *Neuroimage, 15*(1), 273-289. doi:10.1006/nimg.2001.0978

# **Supplementary Figures**


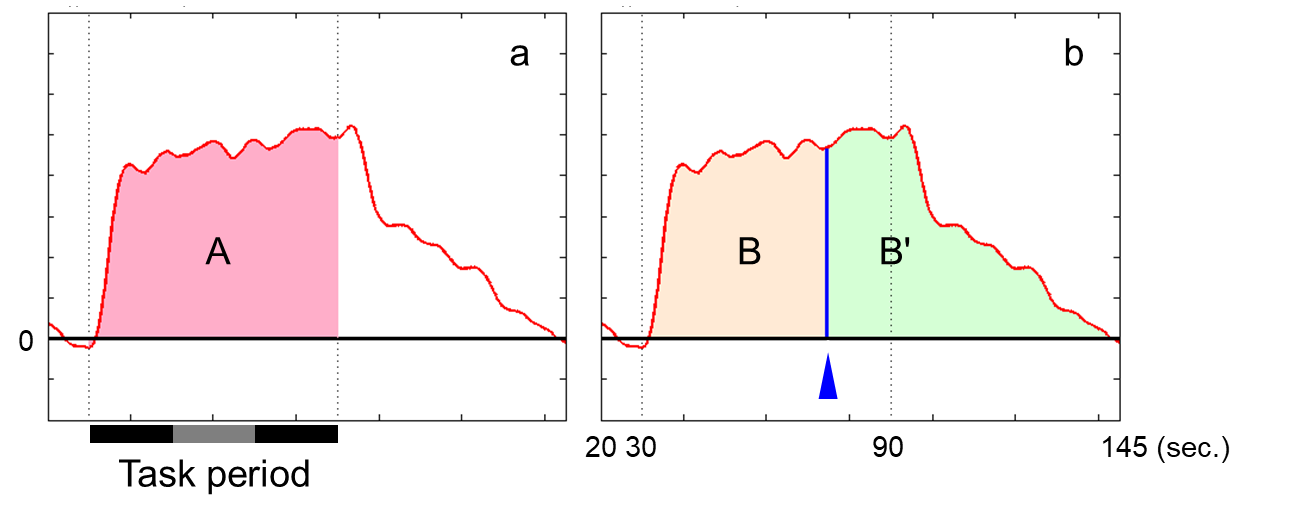


## **Supplementary Figure S1.** **Functional near-infrared spectroscopy (fNIRS) variables in this study.**

The Y-axis shows brain activity (nmol･mm). Previous studies have established 2 fNIRS variables in this study setting: brain activity and activity timing, called Integral and Centroid values, respectively. (a) Brain activity represents the intensity of the hemodynamic response during the task period (area A). (b) Activity timing is defined as the "mean" value of the frequency distribution graph between 10 s before starting the task period and 55 s of the post-task period when we see the positive values of fNIRS signal as a frequency distribution graph (i.e., 78 sec).


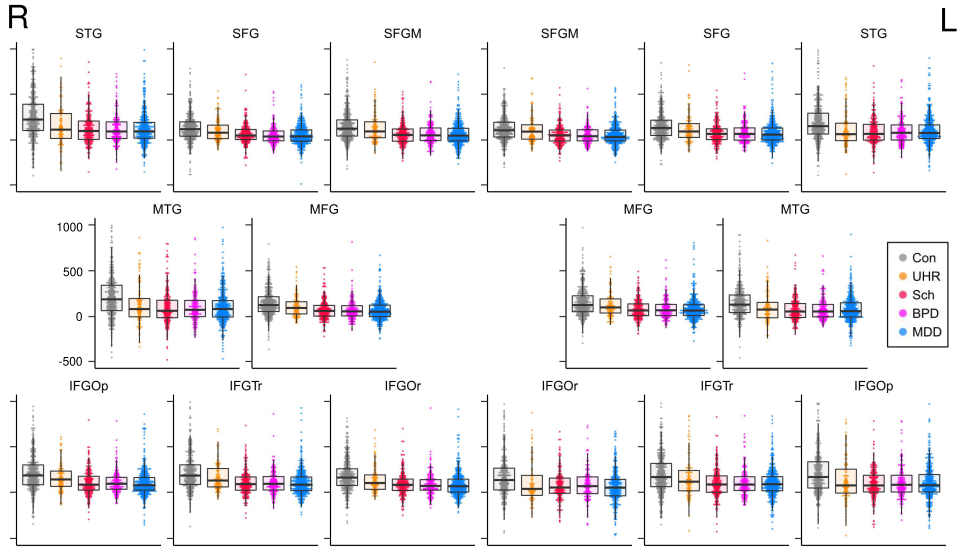


## **Supplementary Figure S2. The difference in brain activity over the fNIRS brain regions.**

High resolution PDF file is also provided. Dot and box plots are illustrated for the brain activity over the prefrontal-temporal cortical area. Y-axes show brain activity (nmol･mm).

Abbreviations: STG, superior temporal gyrus; SFG, superior frontal gyrus; SFGM, superior frontal medial cortex; MTG, and middle temporal gyrus; MFG, middle frontal gyrus; IFGOp, inferior frontal gyrus opercularis; IFGTr, inferior frontal gyrus triangularis; IFGOr, inferior frontal gyrus orbital; Con, healthy controls; UHR, ultra-high risk; Sch, schizophrenia; BPD, bipolar disorder; MDD, major depressive disorder; fNIRS, functional near-infrared spectroscopy


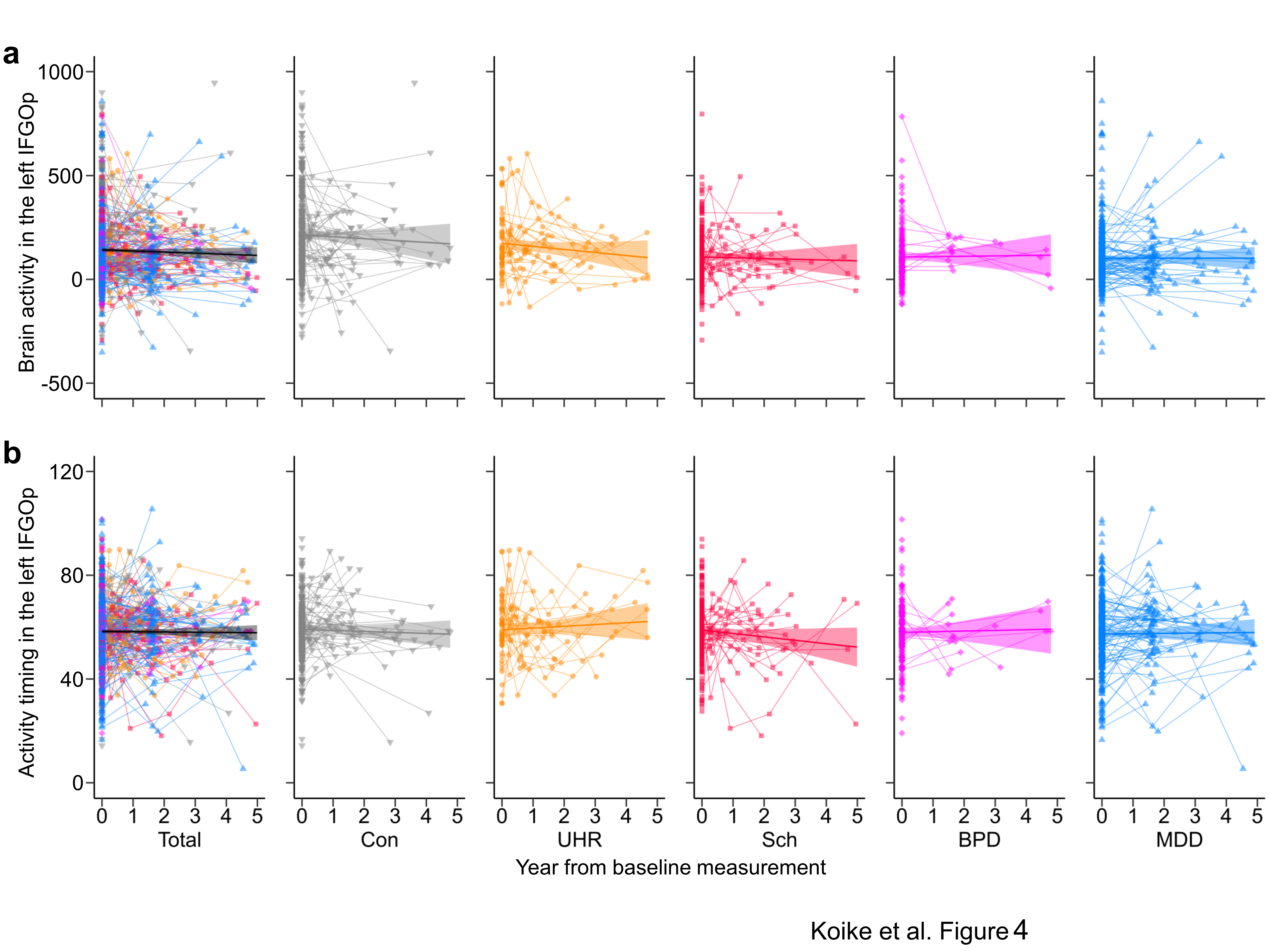


## **Supplementary Figure S3. The effect of repeated measurements on functional near-infrared spectroscopy brain signals.**

The trajectories of (a) brain activity and (b) activity timing in the left inferior frontal gyrus opercularis (IFGOp) for repeated functional near-infrared spectroscopy (fNIRS) measurements are plotted with intervals from the baseline measurement in the total, control (Con), ultra-high risk (UHR), schizophrenia (Sch), bipolar disorder (BPD), and major depressive disorder (MDD) groups. Thin lines show trajectories of repeated measurements for each participant.





## **Supplementary Figure S4. The association between clinical variables and brain activity.**

(a) The relationships between the positive and negative symptom scale (PANSS) general psychopathology scale score and brain activity in the right inferior frontal gyrus orbitalis (IFGOr) in the UHR group, (b) biperiden equivalent dose (mg) and brain activity in the left superior temporal gyrus (STG) in the schizophrenia group, and (c) imipramine equivalent dose (mg) and brain activity in the right inferior frontal gyrus opercularis (IFGOp) in the bipolar disorder (BPD, purple) and major depressive disorder (MDD, blue) groups (Supplementary Tables S27 and S28). Thin lines show trajectories of repeated measurements for each participant.


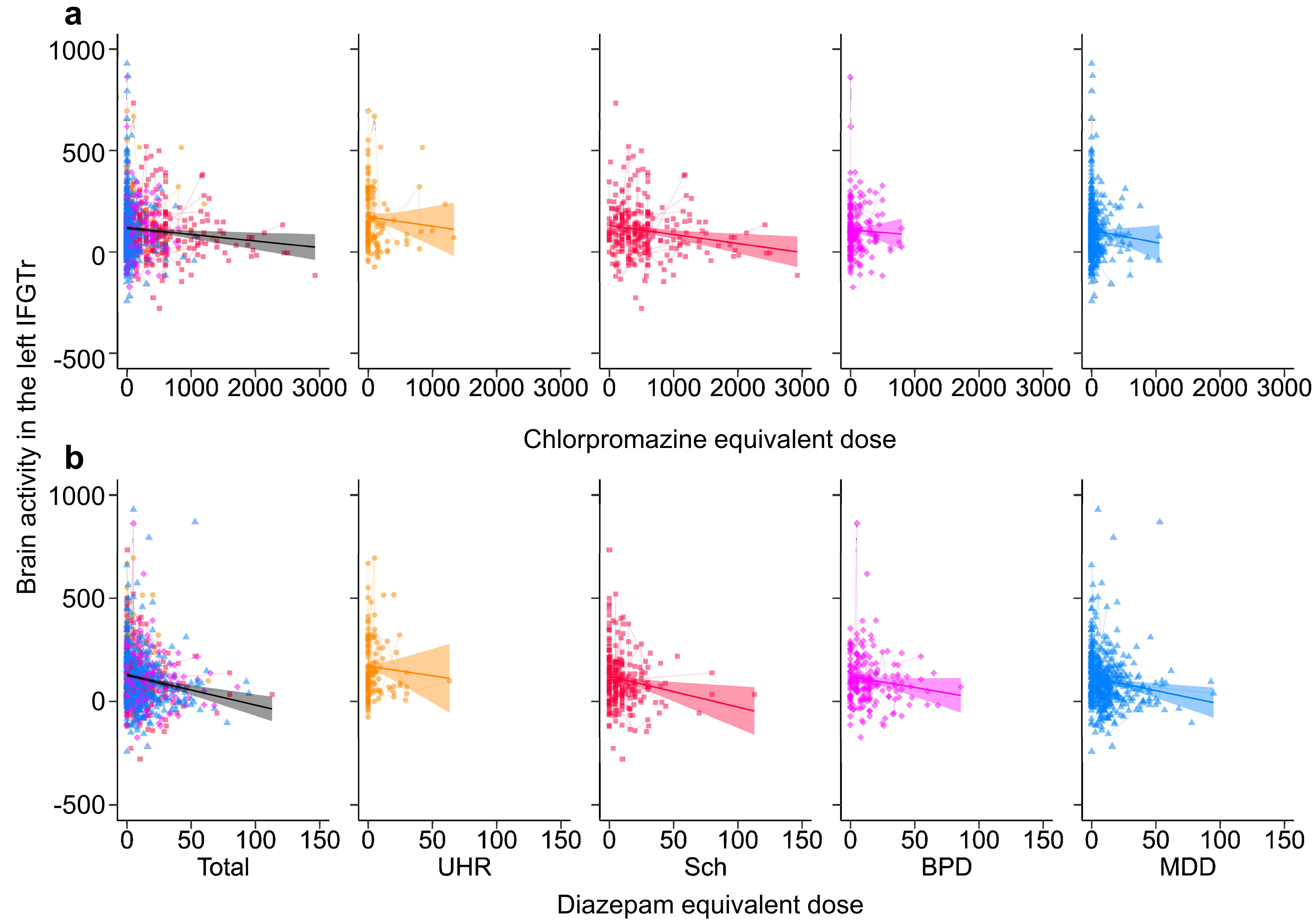


## **Supplementary Figure S5. The association between brain activity in the left inferior frontal gyrus triangularis and medication equivalent dose in the patient groups.**

The relationships between brain activity in the left inferior frontal gyrus triangularis (IFGTr) and (a) chlorpromazine and (b) diazepam equivalent doses (mg) are plotted in the total, ultra-high risk (UHR), schizophrenia (Sch), bipolar disorder (BPD), and major depressive disorder (MDD) groups (Supplementary Table S26). Thin lines show trajectories of repeated measurements for each participant.


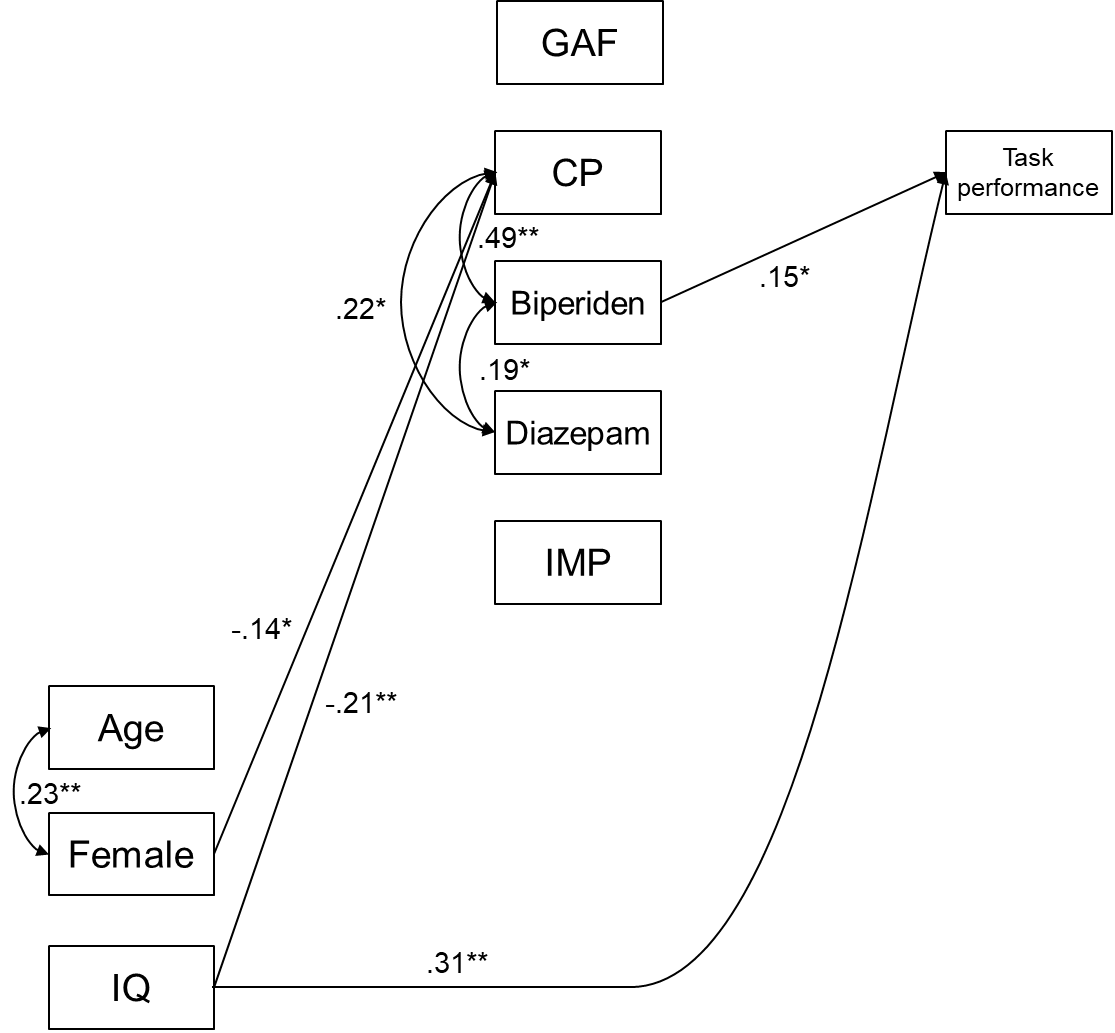


## **Supplementary Figure S6. The base structural equation model including the GAF scale in the schizophrenia group.**

The optimal structural equation model in the schizophrenia group is shown with standard coefficients. We first tested a saturated model including demographic variables, symptom severity, medication dose, and task performance as a base model for each group. After optimizing this base structural equation model (*n* = 204, *χ^2^* = 27.7, *df* = 25, *p* = .32, *confirmatory fit index [CFI]* = 0.98, *root mean square error of approximation [RMSEA]* = 0.023), we compared 16 models of brain activity in each region (Supplementary Table S26).

Abbreviations: IQ, intelligent quotient; GAF, global assessment of functioning; CP, chlorpromazine; IMP, imipramine. (**p* < .05, ***p* < .01)


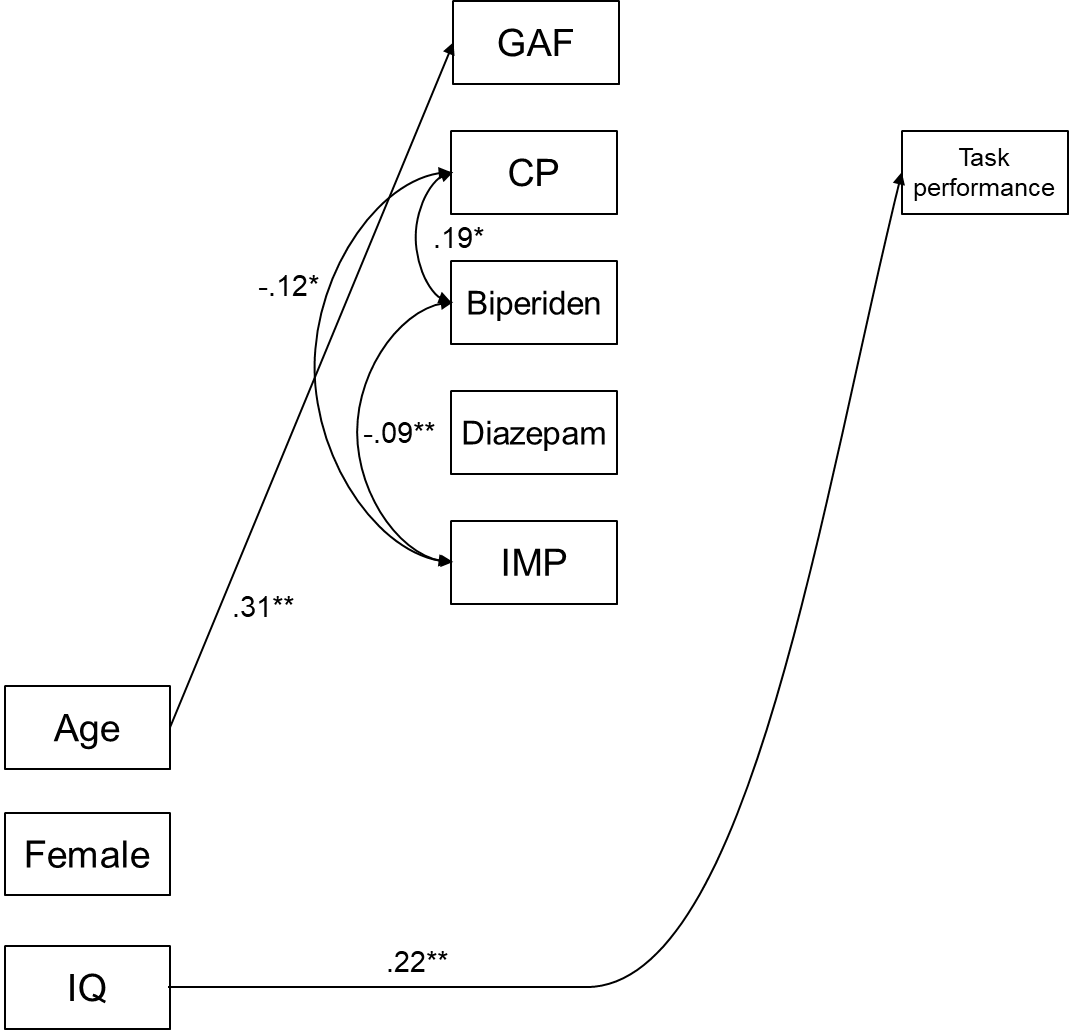


## **Supplementary Figure S7. The base structural equation model including the GAF scale in the bipolar disorder group.**

The optimal structural equation model in the bipolar disorder group is shown with standard coefficients. We first tested a saturated model including demographic variables, symptom severity, medication dose, and task performance as a base model for each group. After optimizing this base structural equation model (*n* = 191, *χ^2^* = 14.4, *df* = 15, *p* = .50, *confirmatory fit index [CFI]* = 1.00, *root mean square error of approximation [RMSEA]* = 0.00), we compared 16 models including brain activity in each region (Supplementary Table S27).

Abbreviations: IQ, intelligent quotient; GAF, global assessment of functioning; CP, chlorpromazine; IMP, imipramine. (**p* < .05, ***p* < .01)


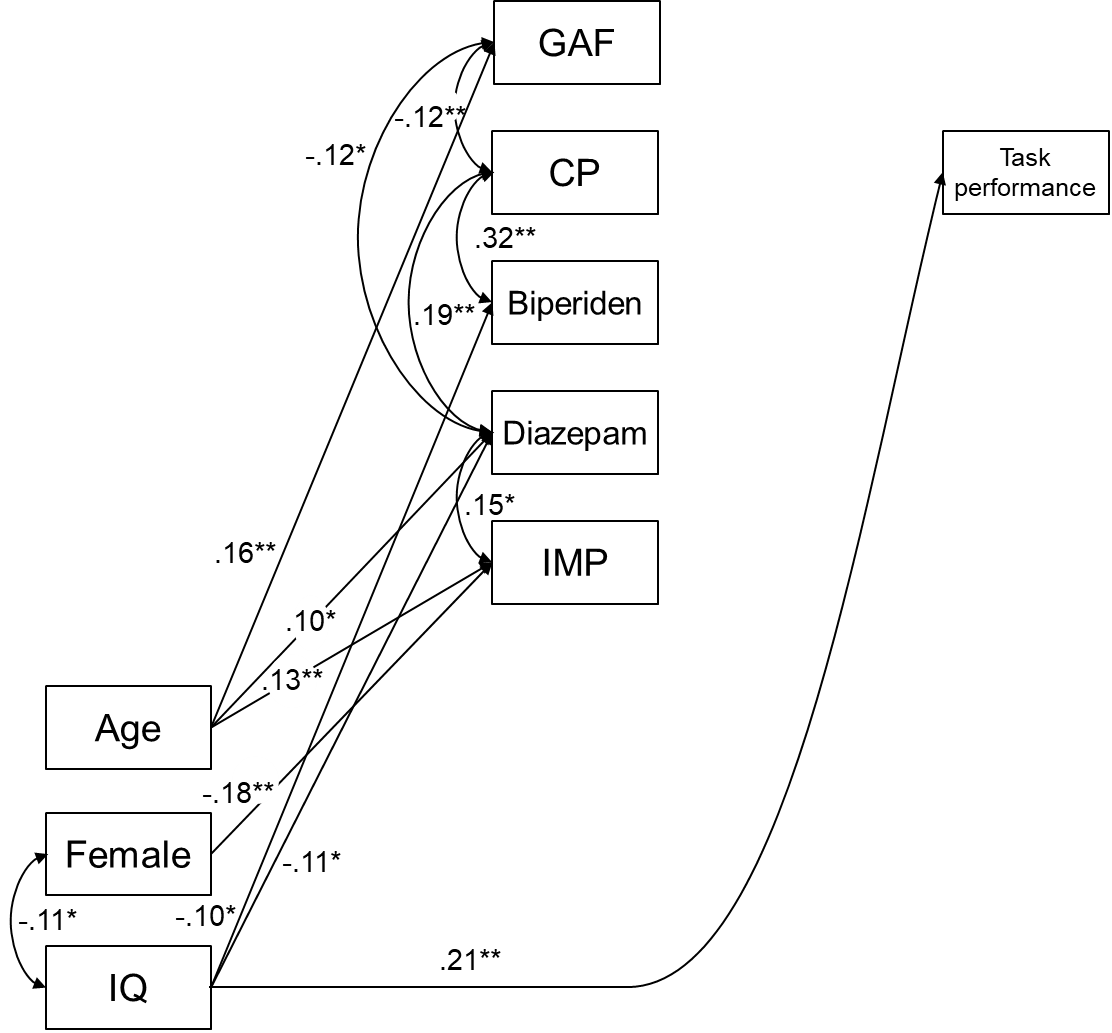


## **Supplementary Figure S8. Base structural equation model including the GAF scale in the major depressive disorder group.**

The optimal structural equation model in the major depressive disorder group was shown with standard coefficients. We first tested a saturated model including demographic variables, symptom severity, medication dose, and task performance as a base model for each group. After optimizing this base structural equation model (*n* = 397, *χ^2^* = 22.0, *df* = 22, *p* = .46, *confirmatory fit index [CFI]* = 1.00, *root mean square error of approximation [RMSEA]* = 0.002), we compared 16 models including brain activity in each region (Supplementary Table S28).

Abbreviations: IQ, intelligent quotient; GAF, global assessment of functioning; CP, chlorpromazine; IMP, imipramine. (**p* < .05, ***p* < .01)

**
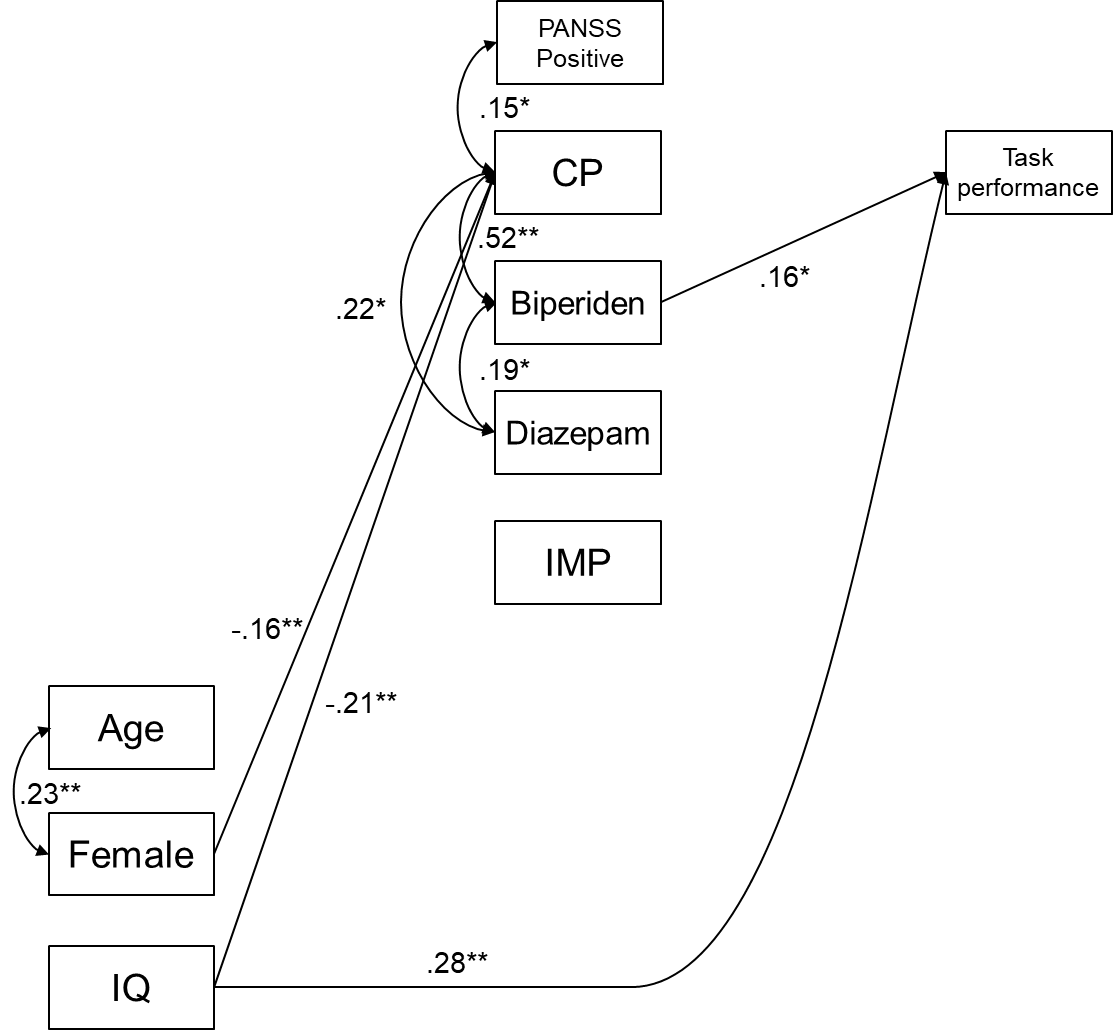
**

## **Supplementary Figure S9. The base structural equation model including the PANSS positive subscale in the schizophrenia group.**

The optimal structural equation model in the schizophrenia group is shown with standard coefficients. We first tested a saturated model including demographic variables, symptom severity, medication dose, and task performance as a base model for each group. After optimizing this base structural equation model (*n* = 204, *χ^2^* = 28.7, *df* = 27, *p* = .38, *confirmatory fit index [CFI]* = 0.99, *root mean square error of approximation [RMSEA]* = 0.017), we compared 16 models of brain activity in each region (Supplementary Table S29).

Abbreviations: IQ, intelligent quotient; GAF, global assessment of functioning; CP, chlorpromazine; IMP, imipramine. (**p* < .05, ***p* < .01)

**
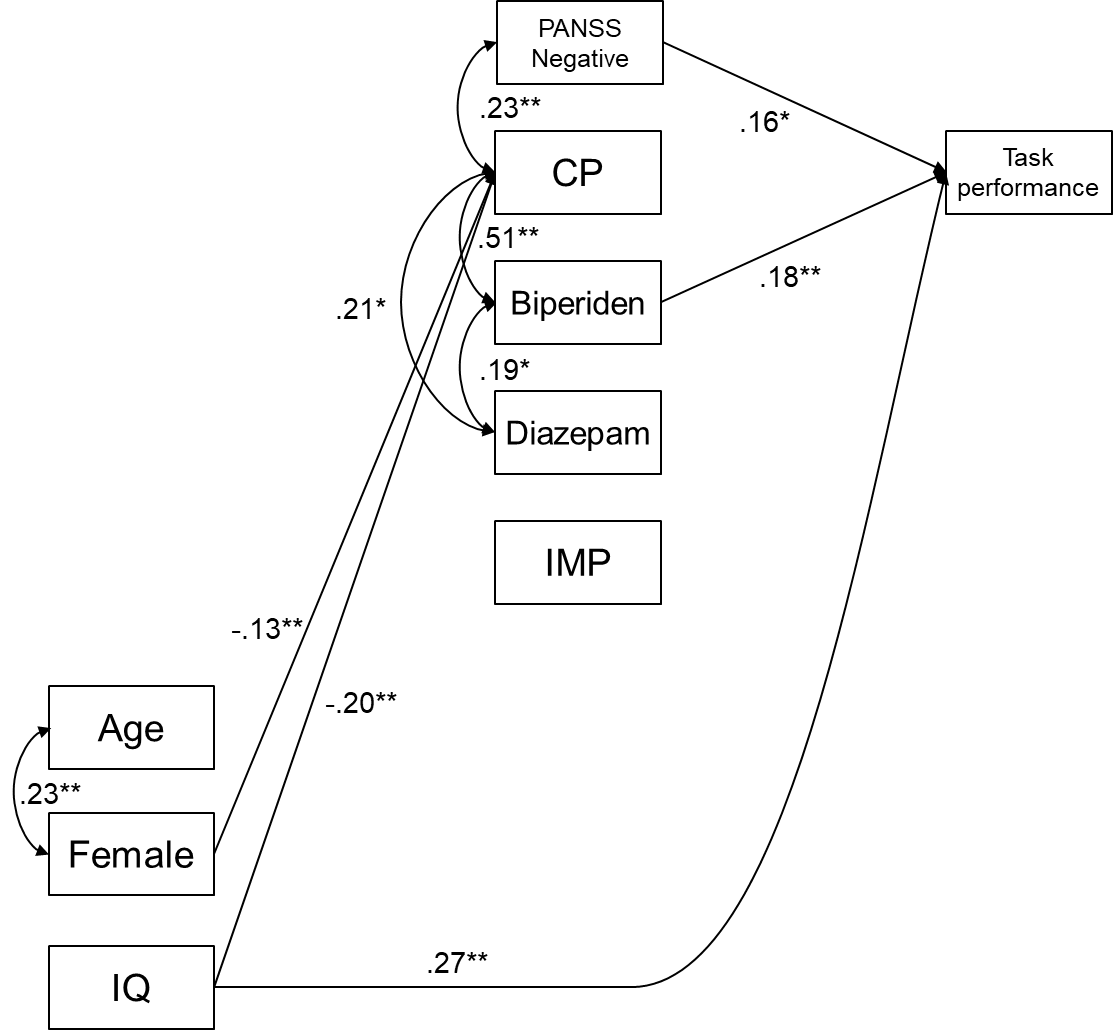
**

## **Supplementary Figure S10. The base structural equation model including the PANSS negative subscale in the schizophrenia group.**

The optimal structural equation model in the schizophrenia group is shown with standard coefficients. We first tested a saturated model including demographic variables, symptom severity, medication dose, and task performance as a base model for each group. After optimizing this base structural equation model (*n* = 204, *χ^2^* = 29.0, *df* = 26, *p* = .31, *confirmatory fit index [CFI]* = 0.98, *root mean square error of approximation [RMSEA]* = 0.024), we compared 16 models of brain activity in each region (Supplementary Table S30).

Abbreviations: IQ, intelligent quotient; GAF, global assessment of functioning; CP, chlorpromazine; IMP, imipramine. (**p* < .05, ***p* < .01)

**
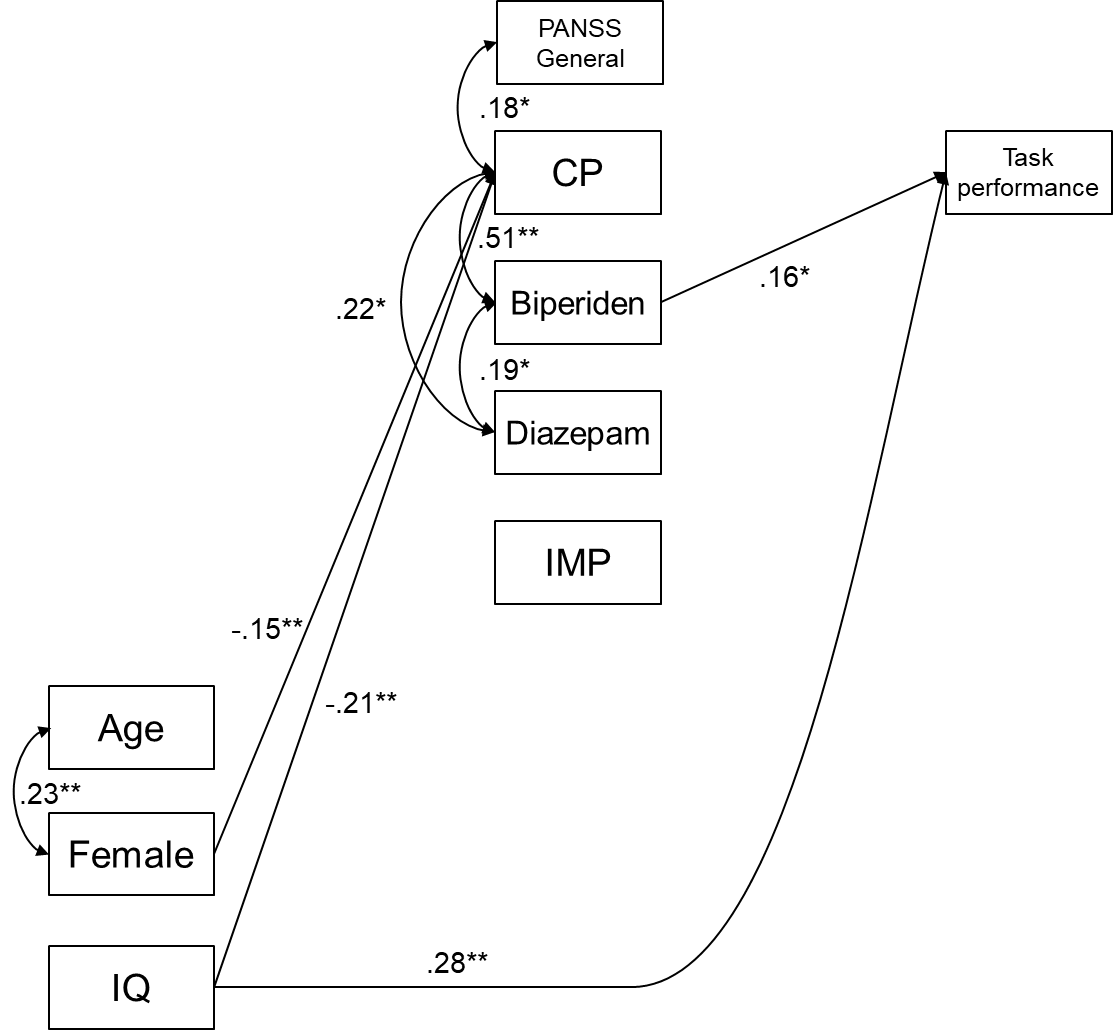
**

## **Supplementary Figure S11. The base structural equation model including the PANSS general psychopathology subscale in the schizophrenia group.**

The optimal structural equation model in the schizophrenia group is shown with standard coefficients. We first tested a saturated model including demographic variables, symptom severity, medication dose, and task performance as a base model for each group. After optimizing this base structural equation model (*n* = 204, *χ^2^* = 24.8, *df* = 27, *p* = .59, *confirmatory fit index [CFI]* = 1.00, *root mean square error of approximation [RMSEA]* = 0.00), we compared 16 models of brain activity in each region (Supplementary Table S31).

Abbreviations: IQ, intelligent quotient; GAF, global assessment of functioning; CP, chlorpromazine; IMP, imipramine. (**p* < .05, ***p* < .01)


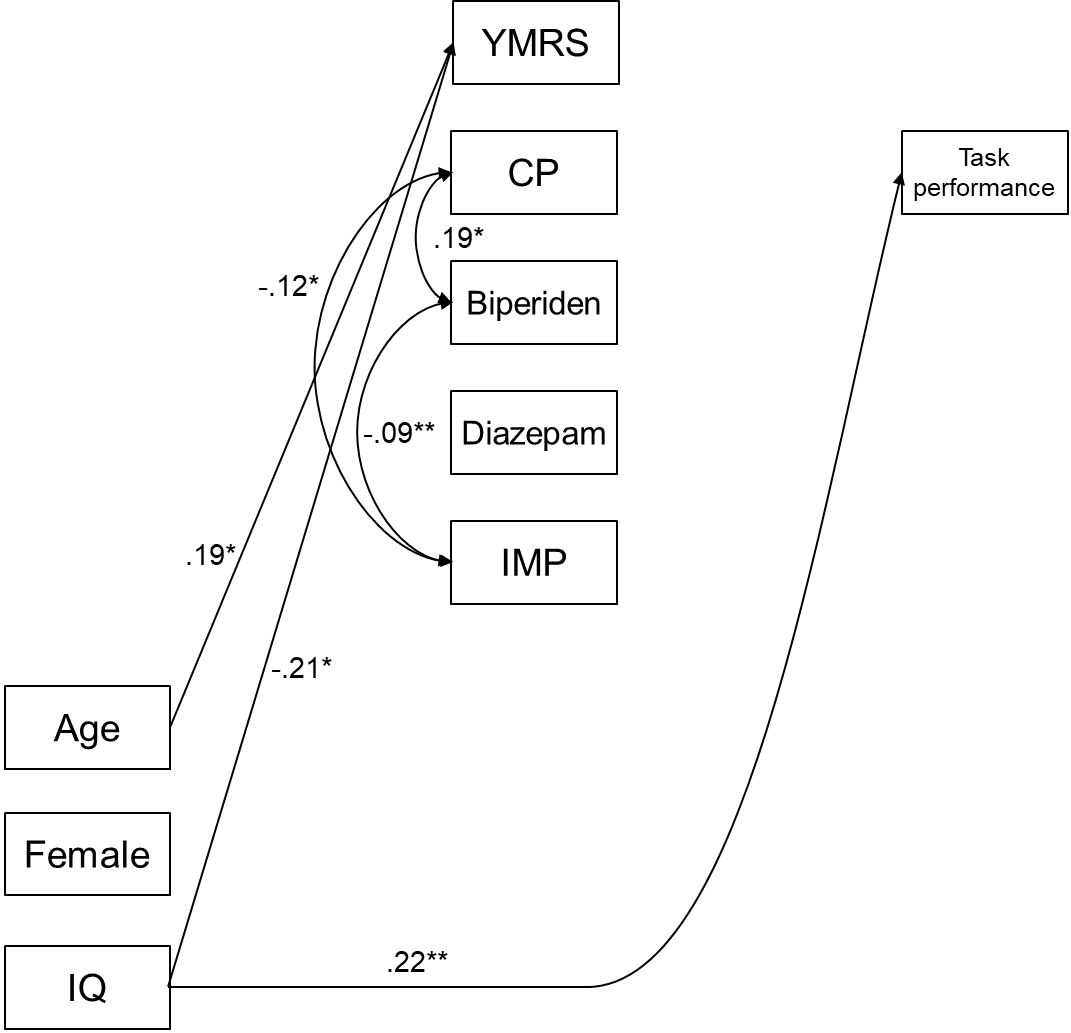


## **Supplementary Figure S12. The base structural equation model including the YMRS scale in the bipolar disorder group.**

The optimal structural equation model in the bipolar disorder group is shown with standard coefficients. We first tested a saturated model including demographic variables, symptom severity, medication dose, and task performance as a base model for each group. After optimizing this base structural equation model (*n* = 191, *χ^2^* = 10.0, *df* = 14, *p* = 0.76, *confirmatory fit index [CFI]* = 1.00, *root mean square error of approximation [RMSEA]* = 0.00), we compared 16 models including brain activity in each region (Supplementary Table S32).

Abbreviations: IQ, intelligent quotient; GAF, global assessment of functioning; CP, chlorpromazine; IMP, imipramine. (**p* < .05, ***p* < .01)


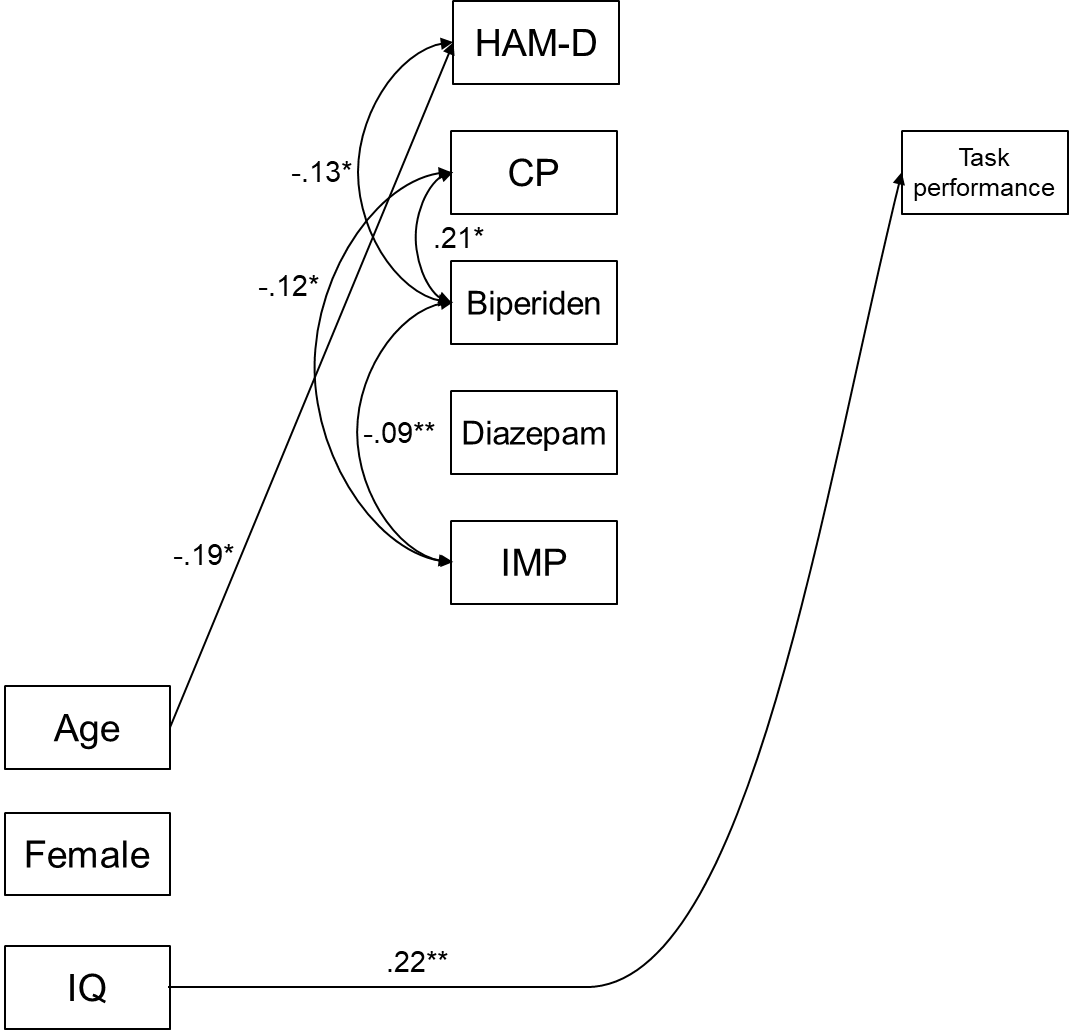


## **Supplementary Figure S13. The base structural equation model including the HAM-D scale in the bipolar disorder group.**

The optimal structural equation model in the bipolar disorder group is shown with standard coefficients. We first tested a saturated model including demographic variables, symptom severity, medication dose, and task performance as a base model for each group. After optimizing this base structural equation model (*n* = 191, *χ^2^* = 15.1, *df* = 17, *p* = 0.59, *confirmatory fit index [CFI]* = 1.00, *root mean square error of approximation [RMSEA]* = 0.00), we compared 16 models including brain activity in each region (Supplementary Table S33).

Abbreviations: IQ, intelligent quotient; GAF, global assessment of functioning; CP, chlorpromazine; IMP, imipramine. (**p* < .05, ***p* < .01)


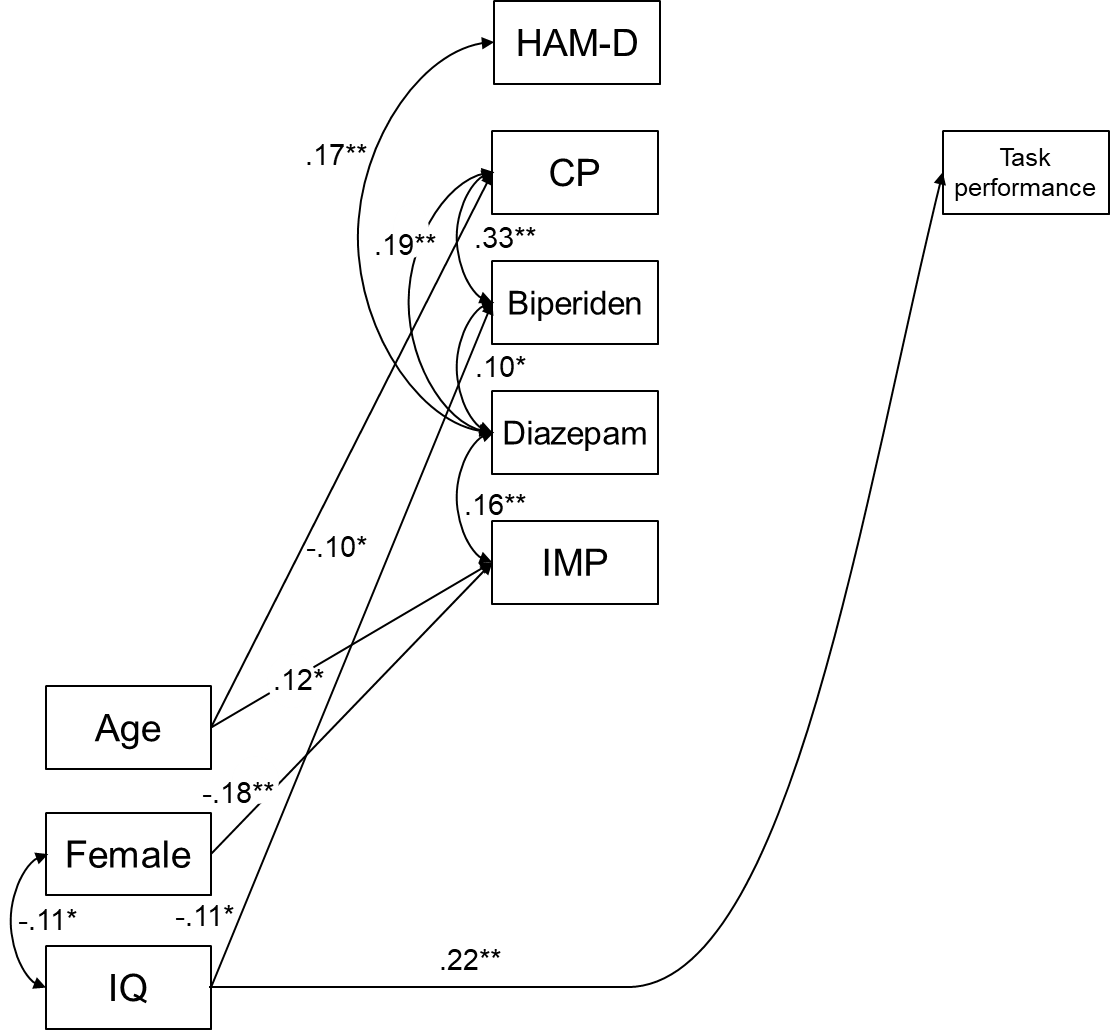


## **Supplementary Figure S14. Base structural equation model including the HAM-D scale in the major depressive disorder group.**

The optimal structural equation model in the major depressive disorder group was shown with standard coefficients. We first tested a saturated model including demographic variables, symptom severity, medication dose, and task performance as a base model for each group. After optimizing this base structural equation model (*n* = 398, *χ^2^* = 23.8, *df* = 25, *p* = 0.53, *confirmatory fit index [CFI]* = 1.00, *root mean square error of approximation [RMSEA]* = 0.00), we compared 16 models including brain activity in each region (Supplementary Table S34).

Abbreviations: IQ, intelligent quotient; GAF, global assessment of functioning; CP, chlorpromazine; IMP, imipramine. (**p* < .05, ***p* < .01)

# **Supplementary Tables**

## **Supplementary Table S1. Demographic characteristics in this study.**

|  |  | Controls | UHR | Schizophrenia | BPD | MDD | p values | Post-hoc test |
| --- | --- | --- | --- | --- | --- | --- | --- | --- |
|  | n | 369 | 52 | 196 | 189 | 394 |  |  |
|  | Female (%) | 185 (50.1) | 22 (42.3) | 99 (50.5) | 82 (43.4) | 196 (49.7) | 0.445 |  |
|  | Age (y, mean (SD)) | 36.79 (13.44) | 23.37 (13.17) | 34.40 (11.49) | 38.39 (10.24) | 41.05 (12.90) | <0.001 | U < CSBM, C < M, S < BM |
|  | T3-FPz-T4 (cm, mean (SD)) | 29.75 (1.71) | 29.69 (1.62) | 29.95 (1.78) | 30.22 (1.65) | 29.95 (1.61) | 0.029 | C < B |
|  | Handedness (mean (SD)) | 90.01 (27.72) | 83.60 (30.25) | 90.17 (21.75) | 82.86 (30.16) | 88.32 (22.88) | 0.019 | B < C |
|  | Estimated IQ (mean (SD)) | 108.31 (9.17) | 106.11 (9.17) | 103.38 (10.87) | 107.43 (9.07) | 106.72 (9.43) | <0.001 | S < CBM |
|  | Task performance (mean (SD)) | 15.74 (4.48) | 13.77 (4.92) | 13.22 (4.73) | 14.53 (4.60) | 14.20 (4.28) | <0.001 | SM < C, S < B |
|  | Sleepiness (mean (SD)) | 2.28 (0.93) | 3.33 (1.00) | 2.98 (1.07) | 3.26 (1.06) | 3.27 (1.03) | <0.001 | C < USBM |
| Symptome severity | |  |  |  |  |  |  |  |
|  | PANSS |  |  |  |  |  |  |  |
|  | Positive symptom (mean (SD)) |  | 14.43 (3.80) | 15.74 (5.26) |  |  | 0.096 |  |
|  | Negative symptom (mean (SD)) |  | 18.27 (5.81) | 19.74 (6.91) |  |  | 0.166 |  |
|  | General psychopathology (mean (SD)) | | 36.29 (8.53) | 36.30 (9.46) |  |  | 0.995 |  |
|  | Total (mean (SD)) |  | 69.00 (16.26) | 71.79 (19.01) |  |  | 0.339 |  |
|  | GAF (mean (SD)) |  | 46.23 (11.31) | 43.26 (12.90) | 44.37 (12.59) | 45.81 (12.74) | 0.105 |  |
|  | HAM-D (mean (SD)) |  |  |  | 11.39 (7.22) | 11.68 (6.80) | 0.642 |  |
|  | YMRS (mean (SD)) |  |  |  | 3.03 (5.05) |  |  |  |
| Medication equivalent dose | |  |  |  |  |  |  |  |
|  | Chlorpromazine (mg, mean (SD)) |  | 131.31 (281.01) | 555.82 (542.25) | 107.02 (165.40) | 53.49 (123.53) | <0.001 | UBM < S |
|  | Biperiden (mg, mean (SD)) |  | 0.04 (0.28) | 1.95 (2.34) | 0.25 (1.00) | 0.18 (0.69) | <0.001 | UBM < S |
|  | Diazepam (mg, mean (SD)) |  | 4.80 (7.52) | 10.97 (15.51) | 13.99 (15.59) | 11.67 (13.64) | 0.001 | U < SBM |
|  | Imipramine (mg, mean (SD)) |  | 14.90 (38.19) | 15.63 (41.92) | 93.43 (125.59) | 125.90 (126.07) | <0.001 | US < B < M |
| Number of measurements (n) | | 457 | 131 | 270 | 207 | 493 |  |  |
| Number of repeated measurements (n, %) | |  |  |  |  |  |  |  |
|  | 1 | 369 (80.7) | 52 (39.7) | 196 (72.6) | 189 (91.3) | 394 (79.9) |  |  |
|  | 2 | 60 (13.1) | 30 (22.9) | 33 (12.2) | 13 (6.3) | 81 (16.4) |  |  |
|  | 3 | 18 (3.9) | 23 (17.6) | 18 (6.7) | 5 (2.4) | 18 (3.7) |  |  |
|  | 4 | 6 (1.3) | 13 (9.9) | 12 (4.4) | 0 (0.0) | 0 (0.0) |  |  |
|  | 5 | 4 (0.9) | 10 (7.6) | 8 (3.0) | 0 (0.0) | 0 (0.0) |  |  |
|  | 6 | 0 (0.0) | 3 (2.3) | 3 (1.1) | 0 (0.0) | 0 (0.0) |  |  |

Abbreviations: SD, standard deviation; IQ, intelligent quotient; PANSS, the positive and negative symptom scale; GAF, the global assessment of functioning; HAM-D, the Hamilton Depression Rating Scale; YMRS, the Young mania rating scale; UHR, ultra-high risk; BPD, bipolar disorder; MDD, major depressive disorder.

## **Supplementary Table S2. The number of missing values**

|  | |  | Controls | UHR | Schizophrenia | BPD | MDD |
| --- | --- | --- | --- | --- | --- | --- | --- |
| n | |  | **369** | **52** | **196** | **189** | **394** |
| Age | |  | 0 | 0 | 0 | 0 | 0 |
| T3-FPz-T4 | |  | 29 | 0 | 42 | 6 | 10 |
| Handedness | |  | 4 | 2 | 20 | 15 | 13 |
| Estimated IQ | |  | 14 | 0 | 6 | 8 | 6 |
| Task performance | |  | 1 | 0 | 1 | 0 | 1 |
| Sleepiness | |  | 4 | 0 | 6 | 4 | 6 |
| Symptom severity | | |  |  |  |  |  |
|  | PANSS | |  |  |  |  |  |
|  | Positive symptom | | NA | 1 | 8 | NA | NA |
|  | Negative symptom | | NA | 1 | 8 | NA | NA |
|  | General psychopathology | | NA | 1 | 8 | NA | NA |
|  | Total | | NA | 1 | 8 | NA | NA |
|  | GAF | | NA | 0 | 5 | 1 | 1 |
|  | HAM-D | | NA | NA | NA | 6 | 6 |
|  | YMRS | | NA | NA | NA | 17 | NA |
| Medication equivalent dose | | |  |  |  |  |  |
|  | Chlorpromazine | | NA | 0 | 6 | 3 | 7 |
|  | Biperiden | | NA | 0 | 6 | 3 | 7 |
|  | Diazepam | | NA | 0 | 6 | 4 | 7 |
|  | Imipramine | | NA | 0 | 86 | 3 | 7 |

Abbreviations: IQ, intelligent quotient; PANSS, positive and negative symptom scale; GAF, global assessment of functioning; HAM-D, Hamilton Depression Rating Scale; YMRS, Young Mania Rating Scale; UHR, ultra-high risk; BPD, bipolar disorder; MDD, major depressive disorder; NA, not applicable.

## **Supplementary Table S3. The correlation matrix for the combined patient groups**

|  | Age | IQ | TP | GAF | CP | BIP | DZP |
| --- | --- | --- | --- | --- | --- | --- | --- |
| IQ | **.10*** |  |  |  |  |  |  |
| TP | .04 | **.24**** |  |  |  |  |  |
| GAF | **.18**** | .07 | .00 |  |  |  |  |
| CP | **-.15**** | **-.22**** | -.06 | **-.12**** |  |  |  |
| BIP | **-.07*** | **-.16**** | .00 | .01 | **.61**** |  |  |
| DZP | .05 | -.03 | .05 | -.07 | **.09*** | **.09*** |  |
| IMP | **.18**** | .07 | .06 | .02 | **-.22**** | **-.14**** | **.12**** |

**p* < .05, ***p* < .01. Abbreviations: IQ, intelligent quotient; TP, task performance; GAF, global assessment of functioning; CP, chlorpromazine; BIP, biperiden; DZP, diazepam; IMP, imipramine.

## **Supplementary Table S4. The correlation matrix for the schizophrenia group**

|  | Age | IQ | TP | positive | negative | general | GAF | CP | BIP | DZP |
| --- | --- | --- | --- | --- | --- | --- | --- | --- | --- | --- |
| IQ | .07 |  |  |  |  |  |  |  |  |  |
| TP | .07 | **.26**** |  |  |  |  |  |  |  |  |
| PANSS |  |  |  |  |  |  |  |  |  |  |
| positive | .09 | -.03 | .11 |  |  |  |  |  |  |  |
| negative | .10 | -.07 | **-.17*** | **.48**** |  |  |  |  |  |  |
| general psycho-pathology | .09 | -.04 | -.06 | **.63**** | **.76**** |  |  |  |  |  |
| GAF | .07 | .05 | -.04 | **-.25**** | **-.37**** | **-.32**** |  |  |  |  |
| CP | -.01 | **-.24**** | .01 | .14 | **.28**** | **.21**** | -.11 |  |  |  |
| BIP | .10 | -.08 | .14 | .01 | .08 | .07 | .14 | **.51**** |  |  |
| DZP | .02 | -.01 | .12 | -.10 | -.02 | -.05 | .03 | **.21**** | **.19*** |  |
| IMP | **.24*** | -.05 | -.01 | -.14 | -.02 | -.09 | -.05 | -.07 | -.01 | .11 |

**p* < .05, ***p* < .01. Abbreviations: IQ, intelligent quotient; TP, task performance; PANSS, positive and negative symptom scale; GAF, global assessment of functioning; CP, chlorpromazine; BIP, biperiden; DZP, diazepam; IMP, imipramine.

## **Supplementary Table S5. The correlation matrix for the bipolar disorder group**

|  | Age | IQ | TP | GAF | HAM-D | YMRS | CP | BIP | DZP |
| --- | --- | --- | --- | --- | --- | --- | --- | --- | --- |
| IQ | .09 |  |  |  |  |  |  |  |  |
| TP | .00 | **.22**** |  |  |  |  |  |  |  |
| GAF | **.31**** | .08 | -.02 |  |  |  |  |  |  |
| HAMD | **-.20*** | -.08 | -.03 | **-.26**** |  |  |  |  |  |
| YMRS | **.17*** | **-.19*** | -.08 | .03 | -.06 |  |  |  |  |
| CP | -.05 | -.08 | .05 | -.12 | .14 | -.01 |  |  |  |
| BIP | -.05 | -.05 | .06 | .06 | -.09 | -.04 | **.19*** |  |  |
| DZP | -.03 | -.02 | .06 | -.07 | .11 | .03 | .02 | .09 |  |
| IMP | -.03 | -.08 | .07 | -.02 | -.04 | -.02 | -.12 | -.09 | .07 |

**p* < .05, ***p* < .01. Abbreviations: IQ, intelligent quotient; TP, task performance; GAF, global assessment of functioning; HAM-D, Hamilton Depression Rating Scale; YMRS, Young Mania Rating Scale; CP, chlorpromazine; BIP, biperiden; DZP, diazepam; IMP, imipramine.

## **Supplementary Table S6. The correlation matrix for the major depressive disorder group**

|  | Age | IQ | TP | GAF | HAM-D | CP | BIP | DZP |
| --- | --- | --- | --- | --- | --- | --- | --- | --- |
| IQ | .06 |  |  |  |  |  |  |  |
| TP | .01 | **.21**** |  |  |  |  |  |  |
| GAF | **.15**** | .05 | .03 |  |  |  |  |  |
| HAMD | .09 | -.05 | -.08 | **-.32**** |  |  |  |  |
| CP | -.08 | -.04 | -.09 | **-.15**** | .05 |  |  |  |
| BIP | .00 | **-.13*** | -.06 | -.08 | .04 | **.33**** |  |  |
| DZP | .10 | -.06 | -.01 | **-.12*** | **.18**** | **.18**** | **.10*** |  |
| IMP | **.13*** | .07 | .02 | -.03 | .01 | -.06 | -.05 | **.15**** |

**p* < .05, ***p* < .01. Abbreviations: IQ, intelligent quotient; TP, task performance; GAF, global assessment of functioning; HAM-D, Hamilton Depression Rating Scale; CP, chlorpromazine; BIP, biperiden; DZP, diazepam; IMP, imipramine.

## **Supplementary Table S7. Summary of the effect of demographics on fNIRS signals in the control group**

|  | Brain Activity | Activity Timing |
| --- | --- | --- |
| Sex^1^ | R-SFG, SFGM, MFG, IFGTr, STG, MTG; L-SFG, SFGM, MFG, IFGTr, IFGOp, IFGOr | None |
| Age^1^ | R-IFGTr^4^, STG^4^; L-STG^4^, MTG^4^ | L-STG |
| Handedness | None | None |
| Estimated IQ^2^ | R-MFG, IFGOr | None |
| Task performance^3^ | None | R-SFG, SFGM, MFG L-SFG, SFGM, MFG, IFGTr, IFGOp, IFGOr |
| Sleepiness | None | None |

1-3, Detailed results are shown in Supplementary Tables S8-10, respectively; 4, the main effect of age^2^ was also significant.

Abbreviations: IQ, intelligent quotient; R, right; L, left; SFG, superior frontal gyrus; SFGM, superior frontal medial cortex; MFG, middle frontal gyrus; IFGTr, inferior frontal gyrus triangularis; IFGOp, inferior frontal gyrus opercularis; IFGOr, inferior frontal gyrus orbital; STG, superior temporal gyrus; MTG, middle temporal gyrus; fNIRS, functional near-infrared spectroscopy.

## **Supplementary Table S8. The effect of age and sex on fNIRS signals in the control group**

|  |  |  | Main effect |  |  |  |  |  | Interaction |
| --- | --- | --- | --- | --- | --- | --- | --- | --- | --- |
|  |  |  | (Intercept) | Age | Age^2^ | Age^3^ | Sex |  | Age x Sex |
| Brain activity | |  |  |  |  |  |  |  |  |
|  | Right | SFG | **253.8** |  |  |  | **-66.4** |  |  |
|  |  | SFGM | **237.4** | -1.1 |  |  | **-47.3** |  |  |
|  |  | MFG | **238.1** |  |  |  | **-54.1** |  | -2.4 |
|  |  | IFGTr | 121.2 | 10.3 | **-0.1** |  | **-50.7** |  |  |
|  |  | IFGOp | **212.8** |  |  |  |  |  | -4.1 |
|  |  | IFGOr | **166.6** |  |  |  |  |  |  |
|  |  | STG | 65.8 | **13.6** | **-0.2** |  | **-79.1** |  |  |
|  |  | MTG | **228.3** |  |  |  | **-45.4** |  |  |
|  | Left | SFG | **191.6** |  |  |  | **-39.6** |  |  |
|  |  | SFGM | **280.3** | -1.4 |  |  | **-52.5** |  |  |
|  |  | MFG | **233.5** |  |  |  | **-57.9** |  |  |
|  |  | IFGTr | **338.3** | -1.7 |  |  | **-42.9** |  |  |
|  |  | IFGOp | **288.9** |  |  |  | **-51.0** |  | -3.2 |
|  |  | IFGOr | **316.9** | -1.3 |  |  | **-62.1** |  |  |
|  |  | STG | 36.2 | **14.8** | **-0.2** |  |  |  |  |
|  |  | MTG | -94.8 | **17.7** | **-0.2** |  |  |  |  |
| Activation timing | | |  |  |  |  |  |  |  |
|  | Right | SFG | **56.56** |  |  |  |  |  |  |
|  |  | SFGM | **55.70** |  |  |  |  |  |  |
|  |  | MFG | **58.07** |  |  |  |  |  |  |
|  |  | IFGTr | **56.36** |  |  |  | 2.43 |  |  |
|  |  | IFGOp | **55.26** |  |  |  | 2.91 |  |  |
|  |  | IFGOr | **60.87** |  |  |  |  |  | -0.27 |
|  |  | STG | **57.15** |  |  |  | 2.00 |  | -0.22 |
|  |  | MTG | **55.74** |  |  |  | 2.54 |  |  |
|  | Left | SFG | **52.54** |  |  |  | 2.40 |  |  |
|  |  | SFGM | **56.04** |  |  |  |  |  |  |
|  |  | MFG | **56.73** |  |  |  |  |  |  |
|  |  | IFGTr | **58.66** |  |  |  |  |  |  |
|  |  | IFGOp | **55.64** |  |  |  | 2.30 |  |  |
|  |  | IFGOr | **55.84** |  |  |  |  |  |  |
|  |  | STG | **55.08** | **0.12** |  |  |  |  |  |
|  |  | MTG | **56.30** |  |  |  | 3.11 |  | -0.21 |

Coefficients of the significant independent variables for each dependent variable (fNIRS signal [nM･mm] were listed. Bold shows *FDR-corrected p* < .05.

Abbreviation: SFG, superior frontal gyrus; SFGM, superior frontal medial cortex; MFG, middle frontal gyrus; IFGTr, inferior frontal gyrus triangularis; IFGOp, inferior frontal gyrus opercularis; IFGOr, inferior frontal gyrus orbital; STG, superior temporal gyrus; MTG, middle temporal gyrus.

## **Supplementary Table S9. The effect of estimated IQ on fNIRS signals in the control group**

|  |  |  | Main effect |  | Confounders | |  |  |
| --- | --- | --- | --- | --- | --- | --- | --- | --- |
|  |  |  | IQ |  | (Intercept) | Age | Age^2^ | Sex |
| Brain activity | |  |  |  |  |  |  |  |
|  | Right | SFG |  |  | **252.4** |  |  | **-65.1** |
|  |  | SFGM |  |  | **200.0** |  |  | **-47.3** |
|  |  | MFG | **2.6** |  | -64.9 |  |  | **-38.3** |
|  |  | IFGTr |  |  | 119.7 | 10.4 | **-0.1** | **-51.2** |
|  |  | IFGOp |  |  | -11.8 | 13.1 | **-0.2** |  |
|  |  | IFGOr | **4.4** |  | -309.4 |  |  |  |
|  |  | STG |  |  | 65.5 | **13.4** | **-0.2** | **-78.0** |
|  |  | MTG |  |  | **225.4** |  |  | **-42.5** |
|  | Left | SFG |  |  | **187.2** |  |  | **-35.3** |
|  |  | SFGM |  |  | **234.2** |  |  | **-52.8** |
|  |  | MFG |  |  | **229.4** |  |  | **-53.9** |
|  |  | IFGTr |  |  | **332.1** | -1.5 |  | **-41.5** |
|  |  | IFGOp |  |  | **273.3** |  |  | -40.5 |
|  |  | IFGOr |  |  | **273.8** |  |  | **-63.2** |
|  |  | STG |  |  | 20.6 | **15.5** | **-0.2** |  |
|  |  | MTG |  |  | -108.3 | **18.3** | **-0.2** |  |
| Activation timing | |  |  |  |  |  |  |  |
|  | Right | SFG |  |  | **60.48** | -0.11 |  |  |
|  |  | SFGM |  |  | **55.60** |  |  |  |
|  |  | MFG |  |  | **58.17** |  |  |  |
|  |  | IFGTr |  |  | **56.27** |  |  | 2.50 |
|  |  | IFGOp |  |  | **50.79** | 0.13 |  | 2.85 |
|  |  | IFGOr |  |  | **60.69** |  |  |  |
|  |  | STG |  |  | **56.50** |  |  | 2.29 |
|  |  | MTG |  |  | **55.95** |  |  | 2.33 |
|  | Left | SFG |  |  | **56.07** |  |  |  |
|  |  | SFGM |  |  | **55.97** |  |  |  |
|  |  | MFG |  |  | **56.75** |  |  |  |
|  |  | IFGTr |  |  | **58.64** |  |  |  |
|  |  | IFGOp |  |  | **55.90** |  |  | 2.04 |
|  |  | IFGOr |  |  | **55.80** |  |  |  |
|  |  | STG |  |  | **55.18** | 0.12 |  |  |
|  |  | MTG |  |  | **55.60** |  |  | **3.48** |

Coefficients of the significant independent variables for each dependent variable (fNIRS signal [nM･mm] were listed. Bold shows *FDR-corrected p* < .05.

Abbreviation: SFG, superior frontal gyrus; SFGM, superior frontal medial cortex; MFG, middle frontal gyrus; IFGTr, inferior frontal gyrus triangularis; IFGOp, inferior frontal gyrus opercularis; IFGOr, inferior frontal gyrus orbital; STG, superior temporal gyrus; MTG, middle temporal gyrus.

## **Supplementary Table S10. The effect of task performance on fNIRS signals in the control group**

|  |  |  | Main effect |  | Confounders | |  |  |
| --- | --- | --- | --- | --- | --- | --- | --- | --- |
|  |  |  | Task performance |  | (Intercept) | Age | Age^2^ | sex |
| Brain activity | |  |  |  |  |  |  |  |
|  | Right | SFG |  |  | **255.2** |  |  | **-67.1** |
|  |  | SFGM |  |  | **240.0** | -1.1 |  | **-48.1** |
|  |  | MFG |  |  | **231.4** |  |  | **-51.2** |
|  |  | IFGTr |  |  | 117.0 | 10.4 | **-0.1** | **-49.9** |
|  |  | IFGOp |  |  | -17.3 | **13.3** | **-0.2** |  |
|  |  | IFGOr | 6.7 |  | 60.5 |  |  |  |
|  |  | STG |  |  | 68.3 | **13.5** | **-0.2** | **-79.6** |
|  |  | MTG |  |  | **229.7** |  |  | **-46.1** |
|  | Left | SFG |  |  | **225.5** | -1.1 |  | **-36.3** |
|  |  | SFGM |  |  | **283.8** | -1.4 |  | **-53.6** |
|  |  | MFG |  |  | **234.7** |  |  | **-58.6** |
|  |  | IFGTr |  |  | **338.7** | -1.7 |  | **-43.0** |
|  |  | IFGOp |  |  | 121.9 | 9.4 | **-0.1** | -40.2 |
|  |  | IFGOr |  |  | **318.7** | -1.3 |  | **-62.7** |
|  |  | STG |  |  | 31.3 | **15.0** | **-0.2** |  |
|  |  | MTG |  |  | -97.6 | **17.8** | **-0.2** |  |
| Activation timing | |  |  |  |  |  |  |  |
|  | Right | SFG | **-0.46** |  | **67.60** | -0.11 |  |  |
|  |  | SFGM | **-0.44** |  | **62.61** |  |  |  |
|  |  | MFG | **-0.36** |  | **63.75** |  |  |  |
|  |  | IFGTr |  |  | **56.35** |  |  | 2.43 |
|  |  | IFGOp |  |  | **55.21** |  |  | 2.94 |
|  |  | IFGOr |  |  | **60.66** |  |  |  |
|  |  | STG |  |  | **56.52** |  |  | 2.28 |
|  |  | MTG |  |  | **55.84** |  |  | 2.49 |
|  | Left | SFG | **-0.56** |  | **65.05** |  |  |  |
|  |  | SFGM | **-0.39** |  | **62.24** |  |  |  |
|  |  | MFG | **-0.49** |  | **64.55** |  |  |  |
|  |  | IFGTr | **-0.31** |  | **63.51** |  |  |  |
|  |  | IFGOp | **-0.42** |  | **62.78** |  |  | 1.97 |
|  |  | IFGOr | **-0.49** |  | **63.53** |  |  |  |
|  |  | STG |  |  | **55.06** | **0.12** |  |  |
|  |  | MTG |  |  | **55.67** |  |  | **3.39** |

Coefficients of the significant independent variables for each dependent variable (fNIRS signal [nM･mm] were listed. Bold shows *FDR-corrected p* < .05.

Abbreviation: SFG, superior frontal gyrus; SFGM, superior frontal medial cortex; MFG, middle frontal gyrus; IFGTr, inferior frontal gyrus triangularis; IFGOp, inferior frontal gyrus opercularis; IFGOr, inferior frontal gyrus orbital; STG, superior temporal gyrus; MTG, middle temporal gyrus.

## **Supplementary Table S11. Differences in demographics between patients with first-episode psychosis and chronic schizophrenia**

|  |  | First-episode psychosis  Mean (SD) | Chronic schizophrenia  Mean (SD) | *p* value |
| --- | --- | --- | --- | --- |
|  | n | 51 | 145 |  |
|  | Female (%) | 30 (58.8) | 69 (47.6) | .223 |
|  | Age (y) | 26.69 (9.33) | 37.11 (10.96) | < .001 |
|  | T3–T4 (cm) | 29.73 (1.96) | 30.07 (1.68) | .263 |
|  | Handedness | 90.69 (19.76) | 90.17 (19.24) | .999 |
|  | Premorbid IQ | 104.64 (10.00) | 102.93 (11.16) | .340 |
|  | Task performance | 12.33 (4.26) | 13.53 (4.86) | .119 |
|  | Sleepiness | 2.98 (1.07) | 2.99 (1.08) | .972 |
| Symptom severity | |  |  |  |
|  | PANSS |  |  |  |
|  | Positive symptom | 16.33 (5.17) | 15.53 (5.29) | .350 |
|  | Negative symptom | 19.65 (7.31) | 19.78 (6.78) | .906 |
|  | General psychopathology | 36.43 (9.27) | 36.26 (9.57) | .910 |
|  | Total | 72.41 (18.94) | 71.56 (19.10) | .786 |
|  | GAF | 40.49 (12.99) | 44.26 (12.76) | .073 |
| Medication equivalent dose | |  |  |  |
|  | Chlorpromazine (mg) | 494.49 (430.95) | 578.32 (577.50) | .346 |
|  | Biperiden (mg) | 1.61 (2.31) | 2.08 (2.34) | .224 |
|  | Diazepam (mg) | 8.82 (9.64) | 11.75 (17.14) | .250 |
|  | Imipramine (mg) | 0.57 (3.77) | 25.67 (51.78) | .002 |

Abbreviations: SD, standard deviation; IQ, intelligent quotient; PANSS, positive and negative symptom scale; GAF, global assessment of functioning.

## **Supplementary Table S12. Differences in fNIRS variables between patients with first-episode psychosis and chronic schizophrenia**

|  |  |  | First-episode psychosis | Chronic schizophrenia | p values |
| --- | --- | --- | --- | --- | --- |
|  |  | n | 51 | 145 |  |
| Brain activity | |  |  |  |  |
|  | Right | SFG | 81.84 (124.20) | 73.06 (105.71) | 0.627 |
|  |  | SFGM | 69.94 (109.80) | 48.01 (98.73) | 0.187 |
|  |  | MFG | 96.19 (108.80) | 80.06 (112.59) | 0.376 |
|  |  | IFGTr | 123.29 (154.96) | 120.69 (216.30) | 0.937 |
|  |  | IFGOp | 124.97 (128.58) | 119.15 (218.30) | 0.859 |
|  |  | IFGOr | 66.72 (126.75) | 100.69 (191.27) | 0.282 |
|  |  | STG | 107.14 (123.61) | 98.67 (188.34) | 0.765 |
|  |  | MTG | 82.01 (127.46) | 82.42 (143.28) | 0.986 |
|  | Left | SFG | 86.63 (119.49) | 50.26 (125.11) | 0.072 |
|  |  | SFGM | 74.62 (119.73) | 55.86 (101.41) | 0.280 |
|  |  | MFG | 86.82 (120.19) | 67.82 (110.65) | 0.304 |
|  |  | IFGTr | 111.52 (136.56) | 105.62 (139.72) | 0.794 |
|  |  | IFGOp | 116.56 (119.47) | 109.32 (146.15) | 0.753 |
|  |  | IFGOr | 94.99 (130.76) | 87.52 (129.39) | 0.731 |
|  |  | STG | 124.51 (144.15) | 134.10 (208.74) | 0.763 |
|  |  | MTG | 103.28 (167.73) | 100.56 (227.96) | 0.938 |
| Activation timing | | |  |  |  |
|  | Right | SFG | 58.74 (12.70) | 58.81 (13.49) | 0.649 |
|  |  | SFGM | 59.00 (14.14) | 59.18 (15.02) | 0.537 |
|  |  | MFG | 59.56 (11.06) | 59.80 (11.91) | 0.810 |
|  |  | IFGTr | 59.87 (12.87) | 60.61 (13.13) | 0.343 |
|  |  | IFGOp | 60.33 (13.69) | 59.97 (13.11) | 0.552 |
|  |  | IFGOr | 60.61 (15.43) | 60.61 (16.42) | 0.523 |
|  |  | STG | 60.63 (11.93) | 60.92 (11.28) | 0.982 |
|  |  | MTG | 60.49 (14.77) | 60.50 (14.89) | 0.221 |
|  | Left | SFG | 59.47 (14.31) | 60.13 (15.22) | 0.949 |
|  |  | SFGM | 60.48 (14.18) | 60.92 (15.05) | 0.727 |
|  |  | MFG | 59.57 (13.80) | 60.33 (14.11) | 0.722 |
|  |  | IFGTr | 60.28 (11.67) | 60.91 (11.81) | 0.738 |
|  |  | IFGOp | 58.73 (12.26) | 58.91 (12.66) | 0.632 |
|  |  | IFGOr | 58.96 (15.00) | 58.69 (15.77) | 0.736 |
|  |  | STG | 60.04 (12.98) | 60.63 (13.26) | 0.431 |
|  |  | MTG | 60.67 (14.16) | 61.15 (14.30) | 0.932 |

Bold shows *FDR-corrected p* < .05.

Abbreviation: SFG, superior frontal gyrus; SFGM, superior frontal medial cortex; MFG, middle frontal gyrus; IFGTr, inferior frontal gyrus triangularis; IFGOp, inferior frontal gyrus opercularis; IFGOr, inferior frontal gyrus orbital; STG, superior temporal gyrus; MTG, middle temporal gyrus.

## **Supplementary Table S13. Differences in demographics between patients with type I and II bipolar disorders**

|  |  | BPD I  Mean (SD) | BPD II  Mean (SD) | *p* value |
| --- | --- | --- | --- | --- |
|  | n | 86 | 103 |  |
|  | Female (%) | 34 (39.5) | 48 (46.6) | .407 |
|  | Age (y, mean (SD)) | 38.81 (11.28) | 38.04 (9.32) | .611 |
|  | T3–T4 (cm, mean (SD)) | 30.25 (1.64) | 30.20 (1.66) | .852 |
|  | Handedness | 78.61 (38.57) | 86.48 (19.99) | .086 |
|  | Premorbid IQ | 107.08 (8.38) | 107.71 (9.64) | .642 |
|  | Task performance | 13.78 (4.42) | 15.16 (4.67) | .040 |
|  | Sleepiness | 3.20 (1.02) | 3.32 (1.10) | .446 |
| Symptom severity | |  |  |  |
|  | GAF | 45.67 (13.14) | 43.30 (12.09) | .200 |
|  | HAM-D | 10.82 (7.23) | 11.89 (7.21) | .321 |
|  | YMRS | 3.46 (6.06) | 2.66 (3.93) | .303 |
| Medication equivalent dose | |  |  |  |
|  | Chlorpromazine (mg) | 119.76 (171.43) | 96.52 (160.36) | .342 |
|  | Biperiden (mg) | 0.29 (1.13) | 0.22 (0.90) | .626 |
|  | Diazepam (mg) | 15.87 (17.29) | 12.46 (13.96) | .139 |
|  | Imipramine (mg) | 80.25 (100.73) | 104.28 (142.44) | .195 |

Abbreviations: BPD, bipolar disorder; SD, standard deviation; IQ, intelligent quotient; GAF, global assessment of functioning; HAM-D, Hamilton Depression Rating Scale; YMRS, Young Mania Rating Scale.

## **Supplementary Table S14. Differences in fNIRS variables between patients with type I and II bipolar disorder**

|  |  |  | BPD I | BPD II | p values |
| --- | --- | --- | --- | --- | --- |
|  |  | n | 86 | 103 |  |
| Brain activity | |  |  |  |  |
|  | Right | SFG | 66.23 (101.32) | 99.26 (129.77) | 0.056 |
|  |  | SFGM | 42.51 (85.77) | 69.31 (109.67) | 0.068 |
|  |  | MFG | 73.19 (90.66) | 92.37 (123.26) | 0.232 |
|  |  | IFGTr | 97.26 (140.84) | 116.76 (163.05) | 0.388 |
|  |  | IFGOp | 87.03 (175.30) | 120.50 (165.77) | 0.189 |
|  |  | IFGOr | 79.82 (191.37) | 90.95 (195.38) | 0.726 |
|  |  | STG | 83.41 (130.59) | 108.88 (159.31) | 0.237 |
|  |  | MTG | 63.35 (111.73) | 94.23 (134.75) | 0.097 |
|  | Left | SFG | 44.81 (89.29) | 60.21 (108.98) | 0.296 |
|  |  | SFGM | 53.48 (99.23) | 79.76 (133.35) | 0.132 |
|  |  | MFG | 57.99 (92.56) | 85.81 (125.78) | 0.090 |
|  |  | IFGTr | 87.31 (108.39) | 123.93 (140.98) | 0.050 |
|  |  | IFGOp | 91.62 (112.55) | 117.07 (134.79) | 0.166 |
|  |  | IFGOr | 67.15 (97.45) | 99.61 (142.09) | 0.077 |
|  |  | STG | 120.01 (168.94) | 125.58 (171.65) | 0.824 |
|  |  | MTG | 85.66 (164.28) | 108.77 (174.89) | 0.354 |
| Activation timing | | |  |  |  |
|  | Right | SFG | 54.79 (13.74) | 57.49 (13.21) | 0.171 |
|  |  | SFGM | 55.70 (14.36) | 58.55 (14.43) | 0.180 |
|  |  | MFG | 57.62 (11.58) | 60.07 (9.78) | 0.116 |
|  |  | IFGTr | 60.49 (13.54) | 60.09 (12.21) | 0.833 |
|  |  | IFGOp | 61.33 (15.23) | 62.48 (13.69) | 0.593 |
|  |  | IFGOr | 61.07 (14.54) | 63.91 (16.32) | 0.265 |
|  |  | STG | 61.00 (11.84) | 61.37 (12.54) | 0.837 |
|  |  | MTG | 60.60 (14.40) | 58.53 (13.38) | 0.316 |
|  | Left | SFG | 56.50 (14.76) | 57.04 (14.88) | 0.804 |
|  |  | SFGM | 55.23 (14.12) | 57.47 (14.34) | 0.283 |
|  |  | MFG | 55.93 (14.21) | 57.80 (12.06) | 0.331 |
|  |  | IFGTr | 58.26 (13.16) | 56.77 (11.61) | 0.410 |
|  |  | IFGOp | 59.75 (11.90) | 56.51 (11.60) | 0.060 |
|  |  | IFGOr | 56.54 (14.63) | 56.25 (14.21) | 0.891 |
|  |  | STG | 60.68 (13.76) | 58.80 (13.79) | 0.351 |
|  |  | MTG | 62.64 (13.12) | 61.15 (15.16) | 0.473 |

Bold shows *FDR-corrected p* < .05.

Abbreviation: SFG, superior frontal gyrus; SFGM, superior frontal medial cortex; MFG, middle frontal gyrus; IFGTr, inferior frontal gyrus triangularis; IFGOp, inferior frontal gyrus opercularis; IFGOr, inferior frontal gyrus orbital; STG, superior temporal gyrus; MTG, middle temporal gyrus.

## **Supplementary Table S15. The effect of diagnosis and age interaction on fNIRS signals**

|  |  |  | Main effect | |  |  |  | Age interaction | |  |  |  | Confounders | |  |  |  |  |
| --- | --- | --- | --- | --- | --- | --- | --- | --- | --- | --- | --- | --- | --- | --- | --- | --- | --- | --- |
|  |  |  | UHR | Sch | BPD | MDD |  | UHR | Sch | BPD | MDD |  | (Intercept) | Age | Age^2^ | IQ | TP | Sex |
| Brain activity | | | |  |  |  |  |  |  |  |  |  |  |  |  |  |  |  |
|  | Right | SFG | **-39.6** | **-84.2** | **-73.6** | **-73.4** |  |  |  |  |  |  | **152.1** | **-0.8** |  | **1.3** | **-2.7** | **-42.5** |
|  |  | SFGM | **-30.3** | **-76.5** | **-75.9** | **-70.8** |  |  |  |  |  |  | **110.3** | **-0.8** |  | **1.2** | **-2.3** | **-32.9** |
|  |  | MFG | **-34.1** | **-74.6** | **-70.3** | **-66.1** |  |  |  |  |  |  | 63.8 | **-0.8** |  | 1.8 | -1.5 | **-31.2** |
|  |  | IFGTr | **-61.7** | **-94.6** | **-100.1** | **-93.4** |  |  |  |  |  |  | 7.5 | -0.9 |  | 2.6 |  | **-31.9** |
|  |  | IFGOp | **-70.0** | **-91.2** | **-101.1** | **-100.6** |  |  |  |  |  |  | -81.5 |  |  | 2.7 |  |  |
|  |  | IFGOr | **-53.5** | **-72.0** | **-74.2** | **-87.7** |  |  |  |  |  |  | -39.7 |  |  | **1.9** | **3.4** | **-41.3** |
|  |  | STG | **-80.6** | **-97.7** | **-99.5** | **-87.0** |  |  |  |  |  |  | 92.8 |  |  | 1.6 |  | **-44.9** |
|  |  | MTG | **-62.9** | **-86.5** | **-82.8** | **-78.6** |  |  |  |  |  |  | 95.1 |  |  | 1.1 |  | **-32.2** |
|  | Left | SFG | **-40.4** | **-80.2** | **-82.9** | **-75.8** |  |  |  |  |  |  | **126.1** | **-0.8** |  | **1.0** | **-2.4** | **-24.9** |
|  |  | SFGM | **-29.2** | **-91.7** | **-92.6** | **-89.4** |  | -50.8 |  |  |  |  | **119.4** |  |  | **1.4** | **-3.1** | **-44.0** |
|  |  | MFG | **-38.9** | **-80.0** | **-81.5** | **-82.0** |  |  |  |  | 26.8 |  | **105.9** |  |  | **1.4** | **-2.8** | **-40.2** |
|  |  | IFGTr | **-60.8** | **-107.2** | **-108.6** | **-104.9** |  |  |  |  |  |  | **136.7** | **-0.9** |  | 1.6 |  | **-36.9** |
|  |  | IFGOp | **-57.6** | **-102.7** | **-104.0** | **-103.2** |  |  |  |  |  |  | 116.0 | -0.7 |  | 1.5 |  | **-29.2** |
|  |  | IFGOr | **-56.8** | **-94.7** | **-95.7** | **-90.8** |  |  |  |  |  |  | **172.9** | **-0.9** |  | **1.6** | **-3.2** | **-52.5** |
|  |  | STG | **-119.5** | **-144.4** | **-153.4** | **-147.9** |  |  |  |  |  |  | 114.8 |  |  | 2.0 |  | **-35.4** |
|  |  | MTG | **-85.7** | **-109.3** | **-116.5** | **-111.1** |  |  |  |  |  |  | 55.0 |  |  | 1.6 | 2.7 | **-33.2** |
| Activation timing | | | |  |  |  |  |  |  |  |  |  |  |  |  |  |  |  |
|  | Right | SFG | 2.76 | 1.95 |  |  |  |  |  |  |  |  | **63.08** | **-0.08** |  |  | **-0.20** |  |
|  |  | SFGM | **4.20** | **3.01** |  | 1.96 |  |  |  |  |  |  | **63.03** | -0.07 |  |  | **-0.27** |  |
|  |  | MFG | **2.47** |  |  |  |  |  |  |  |  |  | **58.65** |  |  |  |  |  |
|  |  | IFGTr |  |  |  |  |  |  |  |  |  |  | **57.41** |  |  |  |  | 1.32 |
|  |  | IFGOp |  |  |  |  |  |  |  |  |  |  | **53.11** | **0.09** |  |  |  | **2.36** |
|  |  | IFGOr |  |  |  |  |  |  |  |  |  |  | **54.69** | **0.10** |  |  |  | **2.23** |
|  |  | STG |  |  |  |  |  |  |  |  |  |  | **56.84** |  |  |  |  | **2.27** |
|  |  | MTG |  | 2.08 |  |  |  |  |  |  |  |  | **55.44** |  |  |  |  | **2.70** |
|  | Left | SFG | **4.26** | 2.30 |  |  |  |  |  |  |  |  | **61.37** |  |  |  | **-0.28** |  |
|  |  | SFGM | **4.02** | **4.81** |  | 1.92 |  |  |  |  |  |  | **56.24** |  |  |  |  |  |
|  |  | MFG | **3.26** | **2.84** |  |  |  |  |  |  |  |  | **57.60** |  |  |  | **-0.20** | **1.69** |
|  |  | IFGTr |  | 1.68 |  |  |  |  |  |  |  |  | **55.88** |  |  |  |  | **1.60** |
|  |  | IFGOp |  |  |  | -1.37 |  |  |  |  |  |  | **60.88** |  |  |  | -0.15 |  |
|  |  | IFGOr |  | 2.30 |  |  |  |  |  |  |  |  | **61.24** | **-0.10** |  |  | **-0.23** | 1.53 |
|  |  | STG |  |  |  |  |  |  |  |  |  |  | **59.81** |  |  |  |  |  |
|  |  | MTG |  |  |  |  |  |  |  | -5.29 |  |  | **57.59** |  |  |  |  | **2.19** |

Coefficients of the significant independent variables for each dependent variable (fNIRS signal [nM･mm] were listed. Bold shows *FDR-corrected p* < .05. Confounders in the model comparisons were main effect of age, age2, sex, IQ, and task performance as the results of model comparison in the control group. For a calculation time, we first set age interactions as confounders in this table and then sex interactions (Supplementary Table S16).

Abbreviation: SFG, superior frontal gyrus; SFGM, superior frontal medial cortex; MFG, middle frontal gyrus; IFGTr, inferior frontal gyrus triangularis; IFGOp, inferior frontal gyrus opercularis; IFGOr, inferior frontal gyrus orbital; STG, superior temporal gyrus; MTG, middle temporal gyrus; UHR, ultra-high risk; Sch, schizophrenia; BPD, bipolar disorder; MDD, major depressive disorder; IQ, (estimated or premorbid) intelligent quotient; TP, task performance.

## **Supplementary Table S16. The effect of diagnosis on fNIRS signals**

|  |  |  | Main effect | |  |  |  | Sex interaction | |  |  |  | Confounders | |  |  |  |
| --- | --- | --- | --- | --- | --- | --- | --- | --- | --- | --- | --- | --- | --- | --- | --- | --- | --- |
|  |  |  | UHR | Sch | BPD | MDD |  | UHR | Sch | BPD | MDD |  | (Intercept) | Age | IQ | TP | Sex |
| Brain activity | | | |  |  |  |  |  |  |  |  |  |  |  |  |  |  |
|  | Right | SFG | **-39.6** | **-84.2** | **-73.6** | **-73.4** |  |  |  |  |  |  | **152.1** | **-0.8** | **1.3** | **-2.7** | **-42.5** |
|  |  | SFGM | **-30.3** | **-76.5** | **-75.9** | **-70.8** |  |  |  |  |  |  | **110.3** | **-0.8** | **1.2** | **-2.3** | **-32.9** |
|  |  | MFG | **-34.1** | **-74.6** | **-70.3** | **-66.1** |  |  |  |  |  |  | 63.8 | **-0.8** | **1.8** | -1.5 | **-31.2** |
|  |  | IFGTr | **-61.7** | **-94.6** | **-100.1** | **-93.4** |  |  |  |  |  |  | 7.5 | -0.9 | **2.6** |  | **-31.9** |
|  |  | IFGOp | **-70.0** | **-91.2** | **-101.1** | **-100.6** |  |  |  |  |  |  | -81.5 |  | **2.7** |  |  |
|  |  | IFGOr | **-53.5** | **-72.0** | **-74.2** | **-87.7** |  |  |  |  |  |  | -39.7 |  | **1.9** | **3.4** | **-41.3** |
|  |  | STG | **-80.6** | **-97.7** | **-99.5** | **-87.0** |  |  |  |  |  |  | 92.8 |  | **1.6** |  | **-44.9** |
|  |  | MTG | **-62.9** | **-86.5** | **-82.8** | **-78.6** |  |  |  |  |  |  | 95.1 |  | **1.1** |  | **-32.2** |
|  | Left | SFG | **-40.4** | **-80.2** | **-82.9** | **-75.8** |  |  |  |  |  |  | **126.1** | **-0.8** | **1.0** | **-2.4** | **-24.9** |
|  |  | SFGM | **-39.0** | **-95.2** | **-89.7** | **-83.6** |  |  |  |  |  |  | **164.6** | **-1.0** | **1.3** | **-3.3** | **-42.9** |
|  |  | MFG | **-46.5** | **-81.7** | **-78.4** | **-77.2** |  |  |  |  |  |  | **125.4** | **-0.7** | **1.4** | **-2.9** | **-37.3** |
|  |  | IFGTr | **-63.2** | **-107.7** | **-111.2** | **-103.6** |  |  |  | 2.2 |  |  | **134.5** | **-0.9** | **1.5** |  | **-36.1** |
|  |  | IFGOp | **-52.4** | **-101.8** | **-108.9** | **-106.0** |  |  |  | 2.4 |  |  | 93.0 |  | **1.5** |  | **-29.6** |
|  |  | IFGOr | **-59.5** | **-95.5** | **-98.6** | **-89.5** |  |  |  | 2.3 |  |  | **170.9** | **-0.9** | **1.6** | **-3.3** | **-51.7** |
|  |  | STG | **-119.5** | **-144.4** | **-153.4** | **-147.9** |  |  |  |  |  |  | 114.8 |  | **2.0** |  | **-35.4** |
|  |  | MTG | **-85.7** | **-109.3** | **-116.5** | **-111.1** |  |  |  |  |  |  | 55.0 |  | **1.6** | 2.7 | **-33.2** |
| Activation timing | | | |  |  |  |  |  |  |  |  |  |  |  |  |  |  |
|  | Right | SFG | 2.76 | 1.95 |  |  |  |  |  |  |  |  | **63.08** | **-0.08** |  | **-0.20** |  |
|  |  | SFGM | **4.20** | **3.01** |  | 1.96 |  |  |  |  |  |  | **63.03** | -0.07 |  | **-0.27** |  |
|  |  | MFG | **2.47** |  |  |  |  |  |  |  |  |  | **58.65** |  |  |  |  |
|  |  | IFGTr |  |  |  |  |  |  |  |  |  |  | **57.41** |  |  |  | 1.32 |
|  |  | IFGOp | -3.60 |  |  |  |  | -0.22 |  |  |  |  | **52.92** | **0.10** |  |  | **2.43** |
|  |  | IFGOr |  |  |  |  |  |  | -0.22 |  |  |  | **54.41** | **0.10** |  |  | **2.37** |
|  |  | STG |  |  |  |  |  |  |  |  |  |  | **56.84** |  |  |  | **2.27** |
|  |  | MTG |  | 2.08 |  |  |  |  |  |  |  |  | **55.44** |  |  |  | **2.70** |
|  | Left | SFG | **4.26** | 2.30 |  |  |  |  |  |  |  |  | **61.37** |  |  | **-0.28** |  |
|  |  | SFGM | **4.02** | **4.81** |  | 1.92 |  |  |  |  |  |  | **56.24** |  |  |  |  |
|  |  | MFG | **3.26** | **2.84** |  |  |  |  |  |  |  |  | **57.60** |  |  | **-0.20** | **1.69** |
|  |  | IFGTr |  | 1.68 |  |  |  |  |  |  |  |  | **55.88** |  |  |  | **1.60** |
|  |  | IFGOp |  |  |  | -1.37 |  |  |  |  |  |  | **60.88** |  |  | -0.15 |  |
|  |  | IFGOr |  | 2.30 |  |  |  |  |  |  |  |  | **61.24** | **-0.10** |  | **-0.23** | 1.53 |
|  |  | STG |  |  |  |  |  |  |  |  |  |  | **59.81** |  |  |  |  |
|  |  | MTG |  |  |  |  |  |  |  |  |  |  | **57.78** |  |  |  | **2.09** |

Coefficients of the significant independent variables for each dependent variable (fNIRS signal [nM･mm] were listed. Bold shows *FDR-corrected p* < .05. Confounders in the model comparisons were main effect of age, sex, IQ, and task performance as the results of the first model comparison (Supplementary Table S15) as well as sex interaction for the second analysis.

Abbreviation: SFG, superior frontal gyrus; SFGM, superior frontal medial cortex; MFG, middle frontal gyrus; IFGTr, inferior frontal gyrus triangularis; IFGOp, inferior frontal gyrus opercularis; IFGOr, inferior frontal gyrus orbital; STG, superior temporal gyrus; MTG, middle temporal gyrus; UHR, ultra-high risk; Sch, schizophrenia; BPD, bipolar disorder; MDD, major depressive disorder; IQ, (estimated or premorbid) intelligent quotient; TP, task performance.

## **Supplementary Table S17. The effect of diagnosis on fNIRS signals in the patient group**

|  |  |  | Main effect | |  |  | Confounders | |  |  |  |
| --- | --- | --- | --- | --- | --- | --- | --- | --- | --- | --- | --- |
|  |  |  | UHR | Sch | BPD |  | (Intercept) | Age | IQ | TP | Sex |
| Brain activity | |  |  |  |  |  |  |  |  |  |  |
|  | Right | SFG | **37.3** |  |  |  | 49.2 | -0.8 | **1.4** | **-2.7** | **-34.8** |
|  |  | SFGM | **44.9** |  |  |  | 6.6 | -0.7 | **1.5** | **-2.7** | **-28.1** |
|  |  | MFG | **34.1** |  |  |  | 12.5 | -0.8 | **1.3** |  | **-27.9** |
|  |  | IFGTr | **47.3** |  |  |  | -86.9 |  | **2.2** |  | **-30.1** |
|  |  | IFGOp |  |  |  |  | -178.3 |  | **2.7** |  |  |
|  |  | IFGOr |  |  |  |  | -49.9 |  | **1.4** | 2.9 | **-45.8** |
|  |  | STG |  |  |  |  | -32.5 |  | **1.7** |  | **-32.3** |
|  |  | MTG |  |  |  |  | -10.0 |  | **1.3** |  | **-28.6** |
|  | Left | SFG | **38.8** |  |  |  | 46.5 | -0.7 | **1.0** | **-2.7** | **-21.5** |
|  |  | SFGM | **51.5** |  |  |  | 53.5 | -0.9 | **1.5** | **-3.7** | **-39.9** |
|  |  | MFG | **33.1** |  |  |  | 41.4 | -0.6 | **1.3** | **-2.9** | **-33.1** |
|  |  | IFGTr | **49.0** |  |  |  | 23.4 | -0.7 | **1.5** |  | **-37.5** |
|  |  | IFGOp | **56.8** |  |  |  | -14.6 |  | **1.5** |  | **-28.4** |
|  |  | IFGOr | **37.5** |  |  |  | 77.3 | -0.8 | **1.5** | **-3.2** | **-51.3** |
|  |  | STG |  |  |  |  | -14.6 |  | **1.8** |  | **-33.5** |
|  |  | MTG |  |  |  |  | -64.3 |  | **2.0** |  | **-32.3** |
| Activation timing | |  |  |  |  |  |  |  |  |  |  |
|  | Right | SFG | **3.80** | 2.51 |  |  | **57.21** |  |  |  |  |
|  |  | SFGM | 3.65 | 2.16 |  |  | **57.97** |  |  |  |  |
|  |  | MFG | 2.26 |  |  |  | **58.86** |  |  |  |  |
|  |  | IFGTr |  |  |  |  | **59.16** |  |  |  |  |
|  |  | IFGOp |  |  |  |  | **56.75** |  |  |  | 2.31 |
|  |  | IFGOr |  |  |  |  | **58.64** |  |  |  | 2.26 |
|  |  | STG |  |  |  |  | **56.98** |  |  |  | 2.27 |
|  |  | MTG |  |  |  |  | **55.78** |  |  |  | 2.81 |
|  | Left | SFG | 3.76 | 2.19 |  |  | **57.82** |  |  |  |  |
|  |  | SFGM |  | 2.88 |  |  | **58.15** |  |  |  |  |
|  |  | MFG | 3.02 | 2.86 |  |  | **57.45** |  |  |  |  |
|  |  | IFGTr |  | 2.04 |  |  | **55.78** |  |  |  | 1.51 |
|  |  | IFGOp |  |  |  |  | **58.05** |  |  |  |  |
|  |  | IFGOr |  | 2.19 |  |  | **58.05** | **-0.11** |  |  | 2.17 |
|  |  | STG |  |  |  |  | **60.06** |  |  |  |  |
|  |  | MTG |  |  |  |  | **60.96** |  |  |  |  |

Coefficients of the significant independent variables for each dependent variable (fNIRS signal [nM･mm] were listed. Bold shows *FDR-corrected p* < .05. As the results of the second model comparison (Supplementary Table S16), we set age, sex, IQ, and task performance as covariates and the MDD group as the reference for the main effect of diagnosis.

Abbreviation: SFG, superior frontal gyrus; SFGM, superior frontal medial cortex; MFG, middle frontal gyrus; IFGTr, inferior frontal gyrus triangularis; IFGOp, inferior frontal gyrus opercularis; IFGOr, inferior frontal gyrus orbital; STG, superior temporal gyrus; MTG, middle temporal gyrus; UHR, ultra-high risk; Sch, schizophrenia; BPD, bipolar disorder; IQ, (estimated or premorbid) intelligent quotient; TP, task performance.

## **Supplementary Table S18.** **Summary of the effect of symptom severity and medication doses on fNIRS signals in the patient group.**

| Clinical variables | Groups tested | Brain Activity | Activity Timing |
| --- | --- | --- | --- |
| GAF | All patients | R-IFGOp; L- MTG | R-MTG |
| PANSS positive | UHR, schizophrenia | None | None |
| PANSS negative | UHR, schizophrenia | None | None |
| PANSS general psychopathology | UHR, schizophrenia | None | None |
| HAM-D | MDD, BP | None | None |
| YMRS | BP | None | None |
| CP | All patients | R-STG, L-IFGTr | None |
| BPD | All patients | R-SFG, SFGM, MTG L-SFGM, IFGTr, STG | R-MTG |
| DZP | All patients | R-SFG, SFGM, MFG, IFGTr, IFGOp, STG, MTG; L-SFG, SFGM, MFG, IFGTr, IFGOp, IFGOr, MTG | None |
| IMP | All patients | None | None |

Only main effect for the groups listed were shown. Interaction by each group and within-group analysis were also shown in Supplementary Tables S15-17 and S19-25.

Abbreviation: PANSS, the positive and negative symptom scale; GAF, the global assessment of functioning; HAM-D, the Hamilton Depression Rating Scale; YMRS, the Young mania rating scale; UHR, ultra-high risk; Sch, schizophrenia; BPD, bipolar disorder; MDD, major depressive disorder; R, right; L, left; SFG, superior frontal gyrus; SFGM, superior frontal medial cortex; MFG, middle frontal gyrus; IFGTr, inferior frontal gyrus triangularis; IFGOp, inferior frontal gyrus opercularis; IFGOr, inferior frontal gyrus orbital; STG, superior temporal gyrus; MTG, middle temporal gyrus.

## **Supplementary Table S19. The effect of the GAF score on fNIRS signals in the patient group**

|  |  |  | Main effect |  | GAF interaction | | |  | Confounders | |  |  |  |  |  |  |
| --- | --- | --- | --- | --- | --- | --- | --- | --- | --- | --- | --- | --- | --- | --- | --- | --- |
|  |  |  | GAF |  | UHR | Sch | BPD |  | (Intercept) | age | IQ | TP | Sex | UHR | Sch | BPD |
| Brain activity | |  |  |  |  |  |  |  |  |  |  |  |  |  |  |  |
|  | Right | SFG |  |  | -2.27 |  |  |  | 39.10 | -0.78 | **1.44** | **-2.44** | **-33.24** | **46.06** |  |  |
|  |  | SFGM |  |  |  |  |  |  | 1.07 | -0.64 | **1.48** | **-2.66** | **-27.80** | **43.32** |  |  |
|  |  | MFG |  |  |  |  |  |  | 7.44 | -0.78 | **1.37** |  | **-27.44** | **33.29** |  |  |
|  |  | IFGTr | 0.85 |  |  |  |  |  | -128.63 |  | **2.23** |  | **-28.46** | **43.04** |  |  |
|  |  | IFGOp | **1.42** |  |  |  |  |  | **-245.64** |  | **2.68** |  |  |  |  |  |
|  |  | IFGOr |  |  |  |  |  |  | -61.01 |  | **1.48** | 3.02 | **-44.02** |  |  |  |
|  |  | STG | 0.69 |  |  |  |  |  | -72.91 |  | **1.75** |  | **-31.08** |  |  |  |
|  |  | MTG | 0.67 |  |  |  |  |  | -46.61 |  | **1.29** |  | **-28.11** |  |  |  |
|  | Left | SFG |  |  | -1.62 |  |  |  | 35.74 | -0.76 | **1.08** | **-2.50** | **-20.26** | **45.37** |  |  |
|  |  | SFGM |  |  |  |  |  |  | 47.12 | -0.83 | **1.51** | **-3.62** | **-39.41** | **49.44** |  |  |
|  |  | MFG | 0.53 |  | -2.24 | -1.42 |  |  | 7.70 | -0.74 | **1.43** | **-2.76** | **-32.37** | **38.98** |  |  |
|  |  | IFGTr | 0.77 |  | -1.96 |  |  |  | -16.25 | -0.81 | **1.58** |  | **-35.49** | **52.06** |  |  |
|  |  | IFGOp | 0.68 |  | -1.92 |  |  |  | -49.71 |  | **1.52** |  | **-26.73** | **61.71** |  |  |
|  |  | IFGOr |  |  |  |  |  |  | 69.50 | -0.77 | **1.56** | **-3.16** | **-50.55** | **37.18** |  |  |
|  |  | STG | 1.03 |  |  |  |  |  | -63.30 |  | **1.76** |  | **-32.11** |  |  |  |
|  |  | MTG | **1.23** |  |  |  |  |  | -124.45 |  | **2.02** |  | **-30.90** |  |  |  |
| Activation timing | |  |  |  |  |  |  |  |  |  |  |  |  |  |  |  |
|  | Right | SFG |  |  |  |  |  |  | **57.20** |  |  |  |  | **3.92** | 2.48 |  |
|  |  | SFGM |  |  |  |  |  |  | **57.96** |  |  |  |  | 3.71 | 2.19 |  |
|  |  | MFG |  |  |  |  |  |  | **58.84** |  |  |  |  | 2.36 |  |  |
|  |  | IFGTr |  |  |  |  |  |  | **59.15** |  |  |  |  |  |  |  |
|  |  | IFGOp |  |  |  |  |  |  | **56.74** |  |  |  | 2.30 |  |  |  |
|  |  | IFGOr |  |  |  |  |  |  | **58.60** |  |  |  | 2.27 |  |  |  |
|  |  | STG |  |  |  |  |  |  | **56.96** |  |  |  | 2.27 |  |  |  |
|  |  | MTG | **0.12** |  |  |  |  |  | **49.50** |  |  |  | 2.82 |  | 2.51 |  |
|  | Left | SFG |  |  |  |  |  |  | **58.37** |  |  |  |  | 3.28 |  |  |
|  |  | SFGM |  |  |  |  |  |  | **58.13** |  |  |  |  |  | 2.85 |  |
|  |  | MFG |  |  |  |  |  |  | **57.45** |  |  |  |  | 3.01 | 2.81 |  |
|  |  | IFGTr |  |  |  |  |  |  | **55.77** |  |  |  | 1.52 |  | 1.99 |  |
|  |  | IFGOp |  |  |  |  |  |  | **58.03** |  |  |  |  |  |  |  |
|  |  | IFGOr |  |  |  |  |  |  | **59.23** | **-0.13** |  |  | 2.13 |  |  |  |
|  |  | STG |  |  |  |  |  |  | **60.09** |  |  |  |  |  |  |  |
|  |  | MTG |  |  |  |  |  |  | **60.97** |  |  |  |  |  |  |  |

Coefficients of the significant independent variables for each dependent variable (fNIRS signal [nM･mm] were listed. Bold shows *FDR-corrected p* < .05. The MDD group was set as references of the interaction by group and the main effect (confounder) of diagnosis.

Abbreviation: GAF, the global assessment of functioning; SFG, superior frontal gyrus; SFGM, superior frontal medial cortex; MFG, middle frontal gyrus; IFGTr, inferior frontal gyrus triangularis; IFGOp, inferior frontal gyrus opercularis; IFGOr, inferior frontal gyrus orbital; STG, superior temporal gyrus; MTG, middle temporal gyrus; UHR, ultra-high risk; Sch, schizophrenia; BPD, bipolar disorder; IQ, intelligent quotient; TP, task performance.

## **Supplementary Table S20. The effect of the PANSS general psychopathology on fNIRS signals in the UHR group**

|  |  |  | Main effect |  | Confounders | |  |  |  |
| --- | --- | --- | --- | --- | --- | --- | --- | --- | --- |
|  |  |  | PANSS General  Psychopathology |  | (Intercept) | Age | IQ | TP | Sex |
| Brain activity | |  |  |  |  |  |  |  |  |
|  | Right | SFG | **4.8** |  | **-502.3** |  | **5.4** |  | **-69.2** |
|  |  | SFGM | **3.9** |  | **-430.8** |  | **5.0** |  | **-77.6** |
|  |  | MFG | **4.6** |  | **-410.3** |  | **3.6** | **8.7** | **-79.3** |
|  |  | IFGTr |  |  | 74.3 |  |  | **14.3** | -82.1 |
|  |  | IFGOp | **6.3** |  | -240.6 |  |  | **11.7** |  |
|  |  | IFGOr | **7.3** |  | -285.8 |  |  | **21.0** | **-102.2** |
|  |  | STG | **6.1** |  | -136.5 |  |  | **11.5** | -76.7 |
|  |  | MTG | **4.7** |  | -149.9 |  |  | **6.4** |  |
|  | Left | SFG | **2.6** |  | -214.2 |  | **3.0** |  | **-60.3** |
|  |  | SFGM | **3.6** |  | -381.1 |  | **5.1** |  | **-100.0** |
|  |  | MFG | **2.4** |  | -172.2 |  | 2.7 |  | **-56.7** |
|  |  | IFGTr | **3.4** |  | -247.5 |  | 3.1 | **7.2** | **-88.6** |
|  |  | IFGOp | **3.1** |  | -327.3 |  | **3.8** | **6.3** | -69.0 |
|  |  | IFGOr |  |  | **201.8** |  |  | **6.0** | **-105.7** |
|  |  | STG | **5.8** |  | -234.2 |  |  | **13.9** |  |
|  |  | MTG | **6.5** |  | **-278.3** |  |  | **13.4** |  |
| Activation timing | |  |  |  |  |  |  |  |  |
|  | Right | SFG | -0.27 |  | **77.93** |  |  | -0.54 |  |
|  |  | SFGM | -0.33 |  | **82.95** |  |  | **-0.71** |  |
|  |  | MFG |  |  | **61.22** |  |  |  |  |
|  |  | IFGTr |  |  | **59.11** |  |  |  |  |
|  |  | IFGOp |  |  | **57.59** |  |  |  |  |
|  |  | IFGOr |  |  | **60.22** |  |  |  |  |
|  |  | STG |  |  | **60.63** |  |  |  |  |
|  |  | MTG | -0.37 |  | **74.07** |  |  |  |  |
|  | Left | SFG | -0.33 |  | **80.02** |  |  | -0.52 |  |
|  |  | SFGM | -0.30 |  | **77.59** |  |  | -0.52 |  |
|  |  | MFG |  |  | **60.62** |  |  |  |  |
|  |  | IFGTr |  |  | **59.01** |  |  |  |  |
|  |  | IFGOp | -0.29 |  | **69.62** |  |  |  |  |
|  |  | IFGOr |  |  | **58.82** |  |  |  |  |
|  |  | STG |  |  | **59.96** |  |  |  |  |
|  |  | MTG |  |  | **61.54** |  |  |  |  |

Coefficients of the significant independent variables for each dependent variable (fNIRS signal [nM･mm] were listed. Bold shows *FDR-corrected p* < .05.

Abbreviation: SFG, superior frontal gyrus; SFGM, superior frontal medial cortex; MFG, middle frontal gyrus; IFGTr, inferior frontal gyrus triangularis; IFGOp, inferior frontal gyrus opercularis; IFGOr, inferior frontal gyrus orbital; STG, superior temporal gyrus; MTG, middle temporal gyrus; PANSS, the positive and negative symptom scale; IQ, intelligent quotient; TP, task performance.

## **Supplementary Table S21. The effect of biperiden equivalent dose on fNIRS signals in the patient group**

|  |  |  | Main effect |  | Biperiden interaction | | |  | Confounders | |  |  |  |  |  |  |
| --- | --- | --- | --- | --- | --- | --- | --- | --- | --- | --- | --- | --- | --- | --- | --- | --- |
|  |  |  | Biperiden |  | UHR | Sch | BPD |  | (Intercept) | age | IQ | TP | Sex | UHR | Sch | BPD |
| Brain activity | |  |  |  |  |  |  |  |  |  |  |  |  |  |  |  |
|  | Right | SFG | **-10.2** |  | **-54.4** |  |  |  | 79.2 | **-0.9** | **1.2** | **-2.5** | **-35.4** |  |  |  |
|  |  | SFGM | **-8.8** |  | **-72.8** |  |  |  | 27.0 | **-0.7** | **1.3** | **-2.6** | **-28.2** |  |  |  |
|  |  | MFG |  |  |  | **-9.1** |  |  | 26.3 | **-0.8** | **1.3** |  | **-28.1** | **31.8** |  |  |
|  |  | IFGTr |  |  |  | **-12.0** |  |  | -80.7 |  | **2.2** |  | **-31.3** | **46.3** |  |  |
|  |  | IFGOp |  |  | **94.8** |  |  |  | **58.0** |  |  | **3.3** |  | **73.9** |  |  |
|  |  | IFGOr | -8.8 |  |  |  |  |  | **90.3** |  |  | **4.4** | **-47.8** |  |  |  |
|  |  | STG |  |  | 74.9 |  |  |  | -35.0 |  | **1.7** |  | **-32.6** | 48.2 |  |  |
|  |  | MTG | **-7.4** |  |  |  |  |  | 16.8 |  | **1.1** |  | **-30.3** |  |  |  |
|  | Left | SFG |  |  | **-64.7** | **-13.2** |  |  | 65.1 | **-0.8** | **0.9** | **-2.6** | **-21.3** |  |  |  |
|  |  | SFGM | **-10.9** |  | **-80.4** |  |  |  | 81.8 | **-1.0** | **1.3** | **-3.5** | **-40.0** |  |  |  |
|  |  | MFG |  |  | **-52.4** | **-12.5** |  |  | 62.5 | **-0.7** | **1.2** | **-2.7** | **-33.3** |  |  |  |
|  |  | IFGTr | **-10.4** |  |  |  |  |  | 11.2 |  | **1.4** |  | **-39.2** | **60.6** | 23.6 |  |
|  |  | IFGOp |  |  |  | **-9.8** |  |  | -5.1 |  | **1.5** |  | **-28.5** | **50.9** |  |  |
|  |  | IFGOr |  |  | **-56.9** | **-13.9** |  |  | 99.7 | **-0.9** | **1.4** | **-3.0** | **-51.2** |  |  |  |
|  |  | STG | **-10.8** |  |  |  |  |  | -0.9 |  | **1.8** |  | **-35.6** |  |  |  |
|  |  | MTG |  |  |  | -11.8 |  |  | -59.8 |  | **1.7** | 2.6 | **-35.2** |  |  |  |
| Activation timing | |  |  |  |  |  |  |  |  |  |  |  |  |  |  |  |
|  | Right | SFG |  |  |  |  |  |  | **57.10** |  |  |  |  | 3.91 | 2.51 |  |
|  |  | SFGM |  |  |  |  |  |  | **58.35** |  |  |  |  | 3.38 |  |  |
|  |  | MFG |  |  | -4.11 |  |  |  | **58.84** |  |  |  |  |  |  |  |
|  |  | IFGTr |  |  |  |  |  |  | **59.20** |  |  |  |  |  |  |  |
|  |  | IFGOp |  |  |  |  |  |  | **56.97** |  |  |  | 2.26 |  |  |  |
|  |  | IFGOr |  |  |  |  |  |  | **61.93** |  |  |  |  |  |  |  |
|  |  | STG |  |  |  |  |  |  | **57.11** |  |  |  | 2.21 |  |  |  |
|  |  | MTG | **-2.22** |  |  | **3.45** | 3.72 |  | **55.74** |  |  |  | 2.65 |  | 2.95 |  |
|  | Left | SFG |  |  |  |  |  |  | **58.30** |  |  |  |  | 3.50 |  |  |
|  |  | SFGM |  |  |  |  |  |  | **57.59** |  |  |  |  | 2.92 | 3.22 |  |
|  |  | MFG |  |  |  |  |  |  | **57.37** |  |  |  |  | 3.18 | 2.76 |  |
|  |  | IFGTr |  |  |  |  |  |  | **55.74** |  |  |  | 1.48 |  | 1.93 |  |
|  |  | IFGOp |  |  |  |  |  |  | **57.98** |  |  |  |  |  |  |  |
|  |  | IFGOr |  |  |  |  |  |  | **59.41** | **-0.13** |  |  | 2.00 |  |  |  |
|  |  | STG |  |  |  |  |  |  | **60.07** |  |  |  |  |  |  |  |
|  |  | MTG |  |  |  |  |  |  | **61.00** |  |  |  |  |  |  |  |

Coefficients of the significant independent variables for each dependent variable (fNIRS signal [nM･mm] were listed. Bold shows *FDR-corrected p* < .05. The MDD group was set as references of the interaction by group and the main effect (confounder) of diagnosis.

Abbreviation: SFG, superior frontal gyrus; SFGM, superior frontal medial cortex; MFG, middle frontal gyrus; IFGTr, inferior frontal gyrus triangularis; IFGOp, inferior frontal gyrus opercularis; IFGOr, inferior frontal gyrus orbital; STG, superior temporal gyrus; MTG, middle temporal gyrus; UHR, ultra-high risk; Sch, schizophrenia; BPD, bipolar disorder; IQ, intelligent quotient; TP, task performance.

## **Supplementary Table S22. The effect of biperiden equivalent dose on fNIRS signals in the schizophrenia group**

|  |  |  | Main effect |  | Confounders | |  |  |  |
| --- | --- | --- | --- | --- | --- | --- | --- | --- | --- |
|  |  |  | Biperiden |  | (Intercept) | age | IQ | TP | Sex |
| Brain activity | |  |  |  |  |  |  |  |  |
|  | Right | SFG | **-8.7** |  | **189.3** | -1.4 |  |  | -34.8 |
|  |  | SFGM | **-5.5** |  | **162.1** |  |  | -3.3 | **-35.7** |
|  |  | MFG | **-9.0** |  | **190.7** | -1.5 |  |  | -28.8 |
|  |  | IFGTr | **-14.1** |  | **203.0** |  |  |  | -48.1 |
|  |  | IFGOp | **-13.4** |  | **126.8** |  |  |  |  |
|  |  | IFGOr | **-11.5** |  | **173.6** |  |  |  | -52.7 |
|  |  | STG | **-13.4** |  | **111.5** |  |  |  |  |
|  |  | MTG | **-9.2** |  | **87.7** |  |  |  |  |
|  | Left | SFG | **-8.6** |  | **131.0** | -1.7 |  |  |  |
|  |  | SFGM | **-6.8** |  | **195.1** |  |  | -3.8 | -45.1 |
|  |  | MFG | **-9.1** |  | **185.1** | -1.4 |  |  | -33.4 |
|  |  | IFGTr | **-12.8** |  | **202.6** |  |  |  | -48.8 |
|  |  | IFGOp | **-11.6** |  | **188.0** |  |  |  | -40.8 |
|  |  | IFGOr | **-9.6** |  | **264.5** |  |  | -4.5 | -65.8 |
|  |  | STG | **-14.9** |  | **149.3** |  |  |  |  |
|  |  | MTG | **-11.3** |  | **110.9** |  |  |  |  |
| Activation timing | |  |  |  |  |  |  |  |  |
|  | Right | SFG |  |  | **79.92** |  | **-0.20** |  |  |
|  |  | SFGM |  |  | **67.00** | -0.22 |  |  |  |
|  |  | MFG |  |  | **83.62** |  | **-0.28** | 0.37 |  |
|  |  | IFGTr |  |  | **86.12** |  | **-0.31** | 0.39 |  |
|  |  | IFGOp |  |  | **86.20** |  | **-0.25** |  |  |
|  |  | IFGOr |  |  | **91.80** |  | **-0.30** |  |  |
|  |  | STG |  |  | **55.39** |  |  |  | 3.55 |
|  |  | MTG |  |  | **77.57** |  | **-0.21** |  | 3.86 |
|  | Left | SFG |  |  | **80.35** |  | **-0.20** |  |  |
|  |  | SFGM |  |  | **60.81** |  |  |  |  |
|  |  | MFG |  |  | **82.78** |  | **-0.22** |  |  |
|  |  | IFGTr |  |  | **80.47** |  | **-0.25** | 0.41 |  |
|  |  | IFGOp |  |  | **83.80** |  | **-0.25** |  |  |
|  |  | IFGOr |  |  | **81.31** |  | **-0.21** |  |  |
|  |  | STG |  |  | **80.09** |  | **-0.24** |  | 3.42 |
|  |  | MTG |  |  | **81.60** |  | **-0.21** |  |  |

Coefficients of the significant independent variables for each dependent variable (fNIRS signal [nM･mm] were listed. Bold shows *FDR-corrected p* < .05.

Abbreviation: SFG, superior frontal gyrus; SFGM, superior frontal medial cortex; MFG, middle frontal gyrus; IFGTr, inferior frontal gyrus triangularis; IFGOp, inferior frontal gyrus opercularis; IFGOr, inferior frontal gyrus orbital; STG, superior temporal gyrus; MTG, middle temporal gyrus; IQ, intelligent quotient; TP, task performance.

## **Supplementary Table S23. The effect of diazepam equivalent dose on fNIRS signals in the patient group**

|  |  |  | Main effect |  | Diazepam interaction | | |  | Confounders | |  |  |  |  |  |  |
| --- | --- | --- | --- | --- | --- | --- | --- | --- | --- | --- | --- | --- | --- | --- | --- | --- |
|  |  |  | Diazepam |  | UHR | Sch | BPD |  | (Intercept) | age | IQ | TP | Sex | UHR | Sch | BPD |
| Brain activity | |  |  |  |  |  |  |  |  |  |  |  |  |  |  |  |
|  | Right | SFG | **-1.00** |  |  |  |  |  | 66.59 | -0.71 | **1.30** | **-2.39** | **-35.18** | **32.79** |  |  |
|  |  | SFGM | **-0.76** |  |  |  |  |  | 18.31 | -0.59 | **1.37** | **-2.45** | **-28.16** | **42.18** |  |  |
|  |  | MFG | **-0.78** |  |  |  |  |  | 21.71 | -0.74 | **1.33** |  | **-28.08** | **30.51** |  |  |
|  |  | IFGTr | **-1.05** |  |  |  |  |  | -82.86 |  | **2.34** |  | **-31.06** | **42.50** |  |  |
|  |  | IFGOp | **-1.35** |  |  |  |  |  | -164.90 |  | **2.71** |  |  |  |  |  |
|  |  | IFGOr |  |  |  |  |  |  | -60.82 |  | **1.45** | **3.61** | **-45.36** |  |  |  |
|  |  | STG | **-1.12** |  |  |  |  |  | -28.35 |  | **1.79** |  | **-32.06** |  |  |  |
|  |  | MTG | **-1.05** |  |  |  |  |  | 7.77 |  | **1.20** |  | **-28.65** |  |  |  |
|  | Left | SFG | **-0.79** |  |  |  |  |  | 61.86 | -0.66 | **0.91** | **-2.50** | **-21.79** | **35.76** |  |  |
|  |  | SFGM | **-0.82** |  |  |  |  |  | 67.76 | -0.78 | **1.38** | **-3.41** | **-39.99** | **48.22** |  |  |
|  |  | MFG | **-0.96** |  |  |  |  |  | 60.26 | -0.56 | **1.23** | **-2.61** | **-33.69** | **29.79** |  |  |
|  |  | IFGTr | **-1.23** |  |  |  |  |  | 22.52 |  | **1.46** |  | **-38.98** | **52.64** |  |  |
|  |  | IFGOp | **-1.07** |  |  |  |  |  | -3.02 |  | **1.54** |  | **-28.01** | **46.45** |  |  |
|  |  | IFGOr | **-1.06** |  |  |  |  |  | 95.38 | -0.73 | **1.40** | **-2.90** | **-51.50** | **33.29** |  |  |
|  |  | STG |  |  |  |  |  |  | -30.62 |  | **1.99** |  | **-35.56** |  |  |  |
|  |  | MTG | **-0.94** |  |  |  |  |  | -63.93 |  | **1.83** | 2.64 | **-35.17** |  |  |  |
| Activation timing | |  |  |  |  |  |  |  |  |  |  |  |  |  |  |  |
|  | Right | SFG |  |  |  |  |  |  | **57.09** |  |  |  |  | 3.91 | 2.51 |  |
|  |  | SFGM |  |  |  |  |  |  | **58.34** |  |  |  |  | 3.39 |  |  |
|  |  | MFG |  |  |  |  |  |  | **58.83** |  |  |  |  | 2.24 |  |  |
|  |  | IFGTr |  |  |  |  |  |  | **59.19** |  |  |  |  |  |  |  |
|  |  | IFGOp |  |  |  |  |  |  | **57.03** |  |  |  | 2.20 |  |  |  |
|  |  | IFGOr |  |  |  |  |  |  | **61.92** |  |  |  |  |  |  |  |
|  |  | STG |  |  |  |  |  |  | **57.11** |  |  |  | 2.21 |  |  |  |
|  |  | MTG |  |  |  |  |  |  | **55.29** |  |  |  | 2.72 |  | 2.42 |  |
|  | Left | SFG |  |  |  |  |  |  | **58.29** |  |  |  |  | 3.50 |  |  |
|  |  | SFGM |  |  |  |  |  |  | **57.57** |  |  |  |  | 2.93 | 3.23 |  |
|  |  | MFG |  |  |  |  |  |  | **57.36** |  |  |  |  | 3.18 | 2.77 |  |
|  |  | IFGTr |  |  |  |  |  |  | **55.75** |  |  |  | 1.48 |  | 1.93 |  |
|  |  | IFGOp |  |  |  |  |  |  | **57.99** |  |  |  |  |  |  |  |
|  |  | IFGOr |  |  |  |  |  |  | **59.42** | **-0.13** |  |  | 2.00 |  |  |  |
|  |  | STG | -0.08 |  |  |  |  |  | **60.95** |  |  |  |  |  |  |  |
|  |  | MTG |  |  |  |  |  |  | **61.00** |  |  |  |  |  |  |  |

Coefficients of the significant independent variables for each dependent variable (fNIRS signal [nM･mm] were listed. Bold shows *FDR-corrected p* < .05. The MDD group was set as references of the interaction by group and the main effect (confounder) of diagnosis.

Abbreviation: SFG, superior frontal gyrus; SFGM, superior frontal medial cortex; MFG, middle frontal gyrus; IFGTr, inferior frontal gyrus triangularis; IFGOp, inferior frontal gyrus opercularis; IFGOr, inferior frontal gyrus orbital; STG, superior temporal gyrus; MTG, middle temporal gyrus; UHR, ultra-high risk; Sch, schizophrenia; BPD, bipolar disorder; IQ, intelligent quotient; TP, task performance.

## **Supplementary Table S24. The effect of imipramine equivalent dose on fNIRS signals in the patient group**

|  |  |  | Main effect |  | Imipramine interaction | | |  | Confounders | |  |  |  |  |  |  |
| --- | --- | --- | --- | --- | --- | --- | --- | --- | --- | --- | --- | --- | --- | --- | --- | --- |
|  |  |  | Imipramine |  | UHR | Sch | BPD |  | (Intercept) | age | IQ | TP | Sex | UHR | Sch | BPD |
| Brain activity | |  |  |  |  |  |  |  |  |  |  |  |  |  |  |  |
|  | Right | SFG |  |  |  | **0.73** |  |  | 68.72 | -0.70 |  | **1.32** | **-2.49** | **44.18** | **-41.43** | **26.98** |
|  |  | SFGM |  |  |  | **0.47** |  |  | 3.86 |  |  | **1.28** | **-2.34** | **44.32** | **-32.86** | **49.57** |
|  |  | MFG |  |  |  | **0.77** |  |  | -12.00 |  |  | **1.40** |  | **60.75** | **-32.79** | **35.76** |
|  |  | IFGTr |  |  |  | **1.01** |  |  | -111.85 |  |  | **2.56** |  | **79.94** | **-32.64** | **36.83** |
|  |  | IFGOp |  |  | -0.53 | **0.95** |  |  | **-229.05** |  |  | **3.15** |  | **74.93** |  |  |
|  |  | IFGOr |  |  |  | **1.18** |  |  | 77.20 |  |  |  | **5.17** | **82.20** | **-47.79** |  |
|  |  | STG |  |  |  | **0.83** |  |  | -63.54 |  |  | **2.09** |  | **58.57** | **-35.18** |  |
|  |  | MTG |  |  |  |  |  |  | 13.72 |  |  | **1.08** |  |  | **-29.28** |  |
|  | Left | SFG |  |  |  | **0.57** |  |  | 64.84 | -0.61 |  | **0.91** | **-2.68** | **36.83** | **-27.42** | **33.50** |
|  |  | SFGM |  |  |  | **0.65** |  |  | 86.54 | -0.82 |  | **1.26** | **-3.45** | **38.90** | **-45.68** | **41.42** |
|  |  | MFG |  |  |  | **0.67** |  |  | 42.32 |  |  | **1.21** | **-2.77** | **54.54** | **-39.21** | **35.47** |
|  |  | IFGTr |  |  |  | **0.74** |  |  | -2.31 |  |  | **1.60** |  | **68.44** | **-40.78** | **52.09** |
|  |  | IFGOp |  |  |  | **0.85** |  |  | -35.34 |  |  | **1.77** |  | **70.92** | **-29.66** | **43.26** |
|  |  | IFGOr |  |  |  | **0.68** |  |  | 61.90 |  |  | **1.41** | **-2.70** | **64.45** | **-57.84** | **42.99** |
|  |  | STG |  |  |  | **1.01** |  |  | -59.28 |  |  | **2.22** |  | **76.53** | **-30.65** |  |
|  |  | MTG |  |  | -0.47 | **0.83** |  |  | -161.19 |  |  | **2.44** | 3.07 | **79.30** | **-25.88** |  |
| Activation timing | |  |  |  |  |  |  |  |  |  |  |  |  |  |  |  |
|  | Right | SFG |  |  |  |  |  |  | **57.10** |  |  |  |  | 2.84 |  | **3.91** |
|  |  | SFGM |  |  |  |  |  |  | **61.53** |  |  |  | -0.24 |  |  | 3.59 |
|  |  | MFG |  |  |  |  |  |  | **58.69** |  |  |  |  |  |  | 2.37 |
|  |  | IFGTr |  |  |  |  |  |  | **59.06** |  |  |  |  |  |  |  |
|  |  | IFGOp |  |  |  |  |  |  | **56.71** |  |  |  |  |  | 2.42 |  |
|  |  | IFGOr |  |  |  |  |  |  | **57.39** | **0.12** |  |  |  |  |  |  |
|  |  | STG |  |  |  |  |  |  | **57.53** |  |  |  |  |  | 1.83 |  |
|  |  | MTG |  |  |  |  |  |  | **56.55** |  |  |  |  |  | 2.14 |  |
|  | Left | SFG |  |  |  |  |  |  | **58.14** |  |  |  |  |  |  | 3.65 |
|  |  | SFGM |  |  |  |  |  |  | **57.59** |  |  |  |  | 3.12 |  | 2.92 |
|  |  | MFG |  |  |  |  |  |  | **57.37** |  |  |  |  | 2.34 |  | 3.18 |
|  |  | IFGTr |  |  |  |  |  |  | **58.18** |  |  |  |  |  |  |  |
|  |  | IFGOp |  |  |  |  |  |  | **57.91** |  |  |  |  |  |  |  |
|  |  | IFGOr |  |  |  |  |  |  | **62.16** | **-0.13** |  |  |  |  |  |  |
|  |  | STG |  |  |  |  |  |  | **60.03** |  |  |  |  |  |  |  |
|  |  | MTG |  |  |  |  |  |  | **60.91** |  |  |  |  |  |  |  |

Coefficients of the significant independent variables for each dependent variable (fNIRS signal [nM･mm] were listed. Bold shows *FDR-corrected p* < .05. The MDD group was set as references of the interaction by group and the main effect (confounder) of diagnosis.

Abbreviation: SFG, superior frontal gyrus; SFGM, superior frontal medial cortex; MFG, middle frontal gyrus; IFGTr, inferior frontal gyrus triangularis; IFGOp, inferior frontal gyrus opercularis; IFGOr, inferior frontal gyrus orbital; STG, superior temporal gyrus; MTG, middle temporal gyrus; UHR, ultra-high risk; Sch, schizophrenia; BPD, bipolar disorder; IQ, intelligent quotient; TP, task performance.

## **Supplementary Table S25. The effect of imipramine equivalent dose on fNIRS signals in the MDD group**

|  |  |  | Main effect |  | Confounders | |  |  |  |
| --- | --- | --- | --- | --- | --- | --- | --- | --- | --- |
|  |  |  | Imipramine |  | (Intercept) | age | IQ | TP | Sex |
| Brain activity | |  |  |  |  |  |  |  |  |
|  | Right | SFG | **-0.14** |  | 102.64 | -0.90 | 1.22 | -3.43 | **-33.76** |
|  |  | SFGM | **-0.11** |  | -10.34 |  | 1.07 |  | -23.50 |
|  |  | MFG | **-0.15** |  | **145.32** |  |  |  | -28.39 |
|  |  | IFGTr | **-0.21** |  | -73.46 |  | **2.42** |  | -33.88 |
|  |  | IFGOp | **-0.22** |  | **-287.01** |  | **3.91** |  |  |
|  |  | IFGOr | -0.15 |  | 80.60 |  |  | 4.87 | -42.13 |
|  |  | STG | **-0.17** |  | -82.86 |  | **2.46** |  | -34.91 |
|  |  | MTG |  |  | -66.35 |  | 1.39 |  |  |
|  | Left | SFG | **-0.12** |  | **70.86** |  |  |  |  |
|  |  | SFGM |  |  | **152.17** | -0.98 |  |  | -28.64 |
|  |  | MFG | **-0.14** |  | 42.45 |  | 1.21 | -2.74 | **-30.33** |
|  |  | IFGTr | **-0.15** |  | 0.05 |  | **1.59** |  | -30.98 |
|  |  | IFGOp | **-0.16** |  | **167.63** |  |  |  | -28.68 |
|  |  | IFGOr | **-0.13** |  | 87.28 | -1.20 | **1.67** | -3.69 | **-41.51** |
|  |  | STG |  |  | **183.26** |  |  |  | -37.99 |
|  |  | MTG |  |  | -117.55 |  | **2.54** |  | -32.45 |
| Activation timing | |  |  |  |  |  |  |  |  |
|  | Right | SFG |  |  | **57.37** |  |  |  |  |
|  |  | SFGM |  |  | **57.91** |  |  |  |  |
|  |  | MFG |  |  | **58.35** |  |  |  |  |
|  |  | IFGTr |  |  | **62.97** |  |  | -0.29 |  |
|  |  | IFGOp |  |  | **56.16** | 0.15 |  | -0.45 | 2.94 |
|  |  | IFGOr |  |  | **69.48** |  |  | -0.47 |  |
|  |  | STG |  |  | **64.12** |  |  | -0.29 |  |
|  |  | MTG |  |  | **59.29** |  |  |  |  |
|  | Left | SFG |  |  | **58.05** |  |  |  |  |
|  |  | SFGM |  |  | **57.99** |  |  |  |  |
|  |  | MFG |  |  | **57.50** |  |  |  |  |
|  |  | IFGTr |  |  | **57.86** |  |  |  |  |
|  |  | IFGOp |  |  | **57.42** |  |  |  |  |
|  |  | IFGOr |  |  | **56.69** |  |  |  |  |
|  |  | STG |  |  | **60.39** |  |  |  |  |
|  |  | MTG |  |  | **57.08** |  |  |  | 2.62 |

Coefficients of the significant independent variables for each dependent variable (fNIRS signal [nM･mm] were listed. Bold shows *FDR-corrected p* < .05.

Abbreviation: SFG, superior frontal gyrus; SFGM, superior frontal medial cortex; MFG, middle frontal gyrus; IFGTr, inferior frontal gyrus triangularis; IFGOp, inferior frontal gyrus opercularis; IFGOr, inferior frontal gyrus orbital; STG, superior temporal gyrus; MTG, middle temporal gyrus; IQ, intelligent quotient; TP, task performance.

## **Supplementary Table S26. A list of the best fit models including the GAF scale in the SEM model comparison of the schizophrenia group**

| Region | | Brain activityｰGAF relationship | Brain activityｰTask performance relationship | Chi square | df | p value | CFI | RMSEA | AIC | BIC |
| --- | --- | --- | --- | --- | --- | --- | --- | --- | --- | --- |
| Right | SFG | No relationship | Brain activity ￫ Task performance | 25.7 | 27 | 0.54 | 1.00 | 0.000 | 14744.3 | 14868.8 |
|  | SFGM | Brain activity ￫ GAF | **Brain activity ￫ Task performance** | 25.2 | 26 | 0.51 | 1.00 | 0.000 | 14711.5 | 14839.3 |
|  | MFG | No relationship | Brain activity ￫ Task performance | 26.6 | 27 | 0.49 | 1.00 | 0.000 | 14743.5 | 14868.0 |
|  | IFGTr | No relationship | No relationship | 28.6 | 28 | 0.43 | 1.00 | 0.010 | 14981.1 | 15102.4 |
|  | IFGOp | No relationship | Brain activity ￫ Task performance | 29.5 | 28 | 0.39 | 0.99 | 0.016 | 14913.1 | 15034.4 |
|  | IFGOr | No relationship | No relationship | 27.4 | 28 | 0.50 | 1.00 | 0.000 | 14455.0 | 14576.3 |
|  | STG | No relationship | No relationship | 27.8 | 28 | 0.47 | 1.00 | 0.000 | 14936.2 | 15057.5 |
|  | MTG | No relationship | No relationship | 27.3 | 28 | 0.50 | 1.00 | 0.000 | 14707.4 | 14828.7 |
| Left | SFG | No relationship | **Brain activity ￫ Task performance** | 26.5 | 27 | 0.49 | 1.00 | 0.000 | 14806.4 | 14930.9 |
|  | SFGM | No relationship | **Brain activity ￫ Task performance** | 25.8 | 27 | 0.53 | 1.00 | 0.000 | 14727.2 | 14851.8 |
|  | MFG | No relationship | Brain activity ￫ Task performance | 26.5 | 27 | 0.49 | 1.00 | 0.000 | 14754.0 | 14878.5 |
|  | IFGTr | No relationship | Brain activity ￫ Task performance | 26.2 | 27 | 0.51 | 1.00 | 0.000 | 14822.1 | 14946.7 |
|  | IFGOp | No relationship | Brain activity ￫ Task performance | 26.4 | 27 | 0.50 | 1.00 | 0.000 | 14800.2 | 14924.8 |
|  | IFGOr | No relationship | **Brain activity ￫ Task performance** | 26.5 | 27 | 0.49 | 1.00 | 0.000 | 14696.0 | 14820.5 |
|  | STG | No relationship | No relationship | 27.6 | 28 | 0.48 | 1.00 | 0.000 | 14896.8 | 15018.1 |
|  | MTG | No relationship | No relationship | 28.0 | 28 | 0.47 | 1.00 | 0.000 | 14994.8 | 15116.1 |

The best fit model using the AIC from 16 models was listed for each brain region. Bold shows a relationship with .05 or smaller of p value. Abbreviation: SFG, superior frontal gyrus; SFGM, superior frontal medial cortex; MFG, middle frontal gyrus; IFGTr, inferior frontal gyrus triangularis; IFGOp, inferior frontal gyrus opercularis; IFGOr, inferior frontal gyrus orbital; STG, superior temporal gyrus; MTG, middle temporal gyrus.

## **Supplementary Table S27. A list of the best fit models including the GAF scale in the SEM model comparison of the BPD group**

| Region | | Brain activityｰGAF relationship | Brain activityｰTask performance relationship | Chi square | df | p value | CFI | RMSEA | AIC | BIC |
| --- | --- | --- | --- | --- | --- | --- | --- | --- | --- | --- |
| Right | SFG | Brain activity ￫ GAF | No relationship | 20.1 | 26 | 0.79 | 1.00 | 0.00 | 14772.6 | 14899.0 |
|  | SFGM | No relationship | Brain activity ￫ Task performance | 19.0 | 26 | 0.84 | 1.00 | 0.00 | 14675.0 | 14801.4 |
|  | MFG | Brain activity ￫ GAF | **Brain activity ￫ Task performance** | 18.2 | 25 | 0.83 | 1.00 | 0.00 | 14738.6 | 14868.3 |
|  | IFGTr | Brain activity ￫ GAF | Task performance ￫ Brain activity | 18.1 | 25 | 0.84 | 1.00 | 0.00 | 14837.9 | 14967.6 |
|  | IFGOp | Brain activity ￫ GAF | No relationship | 18.0 | 26 | 0.88 | 1.00 | 0.00 | 14800.7 | 14927.1 |
|  | IFGOr | Brain activity ￫ GAF | No relationship | 18.1 | 26 | 0.87 | 1.00 | 0.00 | 14420.6 | 14547.0 |
|  | STG | No relationship | No relationship | 20.4 | 27 | 0.82 | 1.00 | 0.00 | 14847.6 | 14970.8 |
|  | MTG | Brain activity ￫ GAF | No relationship | 18.5 | 26 | 0.86 | 1.00 | 0.00 | 14711.5 | 14837.9 |
| Left | SFG | No relationship | **Task performance ￫ Brain activity** | 19.7 | 26 | 0.80 | 1.00 | 0.00 | 14703.7 | 14830.2 |
|  | SFGM | No relationship | **Brain activity ￫ Task performance** | 19.0 | 26 | 0.84 | 1.00 | 0.00 | 14769.8 | 14896.2 |
|  | MFG | No relationship | **Task performance ￫ Brain activity** | 20.8 | 26 | 0.75 | 1.00 | 0.00 | 14738.8 | 14865.2 |
|  | IFGTr | No relationship | No relationship | 21.6 | 27 | 0.76 | 1.00 | 0.00 | 14793.9 | 14917.1 |
|  | IFGOp | **Brain activity ￫ GAF** | No relationship | 19.8 | 26 | 0.80 | 1.00 | 0.00 | 14784.1 | 14910.6 |
|  | IFGOr | Brain activity ￫ GAF | No relationship | 20.5 | 26 | 0.77 | 1.00 | 0.00 | 14742.2 | 14868.6 |
|  | STG | No relationship | No relationship | 19.1 | 27 | 0.86 | 1.00 | 0.00 | 14891.3 | 15014.5 |
|  | MTG | No relationship | No relationship | 20.6 | 27 | 0.80 | 1.00 | 0.00 | 14905.5 | 15028.7 |

The best fit model using the AIC from 16 models was listed for each brain region. Bold shows a relationship with .05 or smaller of p value. Abbreviation: SFG, superior frontal gyrus; SFGM, superior frontal medial cortex; MFG, middle frontal gyrus; IFGTr, inferior frontal gyrus triangularis; IFGOp, inferior frontal gyrus opercularis; IFGOr, inferior frontal gyrus orbital; STG, superior temporal gyrus; MTG, middle temporal gyrus.

## **Supplementary Table S28. A list of the best fit models including the GAF scale in the SEM model comparison of the MDD group**

| Region | | Brain activityｰGAF relationship | Brain activityｰTask performance relationship | Chi square | df | p value | CFI | RMSEA | AIC | BIC |
| --- | --- | --- | --- | --- | --- | --- | --- | --- | --- | --- |
| Right | SFG | Brain activity ￫ GAF | **Task performance ￫ Brain activity** | 19.8 | 22 | 0.60 | 1.00 | 0.00 | 30260.5 | 30431.4 |
|  | SFGM | Brain activity ￫ GAF | Task performance ￫ Brain activity | 20.0 | 22 | 0.58 | 1.00 | 0.00 | 30118.3 | 30289.2 |
|  | MFG | No relationship | Task performance ￫ Brain activity | 21.8 | 23 | 0.54 | 1.00 | 0.00 | 30268.1 | 30435.0 |
|  | IFGTr | Brain activity ￫ GAF | No relationship | 19.7 | 23 | 0.66 | 1.00 | 0.00 | 30398.7 | 30565.6 |
|  | IFGOp | Brain activity ￫ GAF | No relationship | 20.8 | 23 | 0.59 | 1.00 | 0.00 | 30286.4 | 30453.3 |
|  | IFGOr | Brain activity ￫ GAF | Brain activity ￫ Task performance | 19.6 | 22 | 0.61 | 1.00 | 0.00 | 29643.3 | 29814.1 |
|  | STG | **Brain activity ￫ GAF** | No relationship | 19.1 | 23 | 0.70 | 1.00 | 0.00 | 30422.0 | 30588.9 |
|  | MTG | **Brain activity ￫ GAF** | No relationship | 19.9 | 23 | 0.65 | 1.00 | 0.00 | 30127.1 | 30294.0 |
| Left | SFG | **Brain activity ￫ GAF** | Task performance ￫ Brain activity | 20.0 | 22 | 0.58 | 1.00 | 0.00 | 30161.0 | 30331.8 |
|  | SFGM | Brain activity ￫ GAF | Task performance ￫ Brain activity | 19.7 | 22 | 0.60 | 1.00 | 0.00 | 30234.7 | 30405.6 |
|  | MFG | Brain activity ￫ GAF | **Task performance ￫ Brain activity** | 19.5 | 22 | 0.61 | 1.00 | 0.00 | 30158.6 | 30329.4 |
|  | IFGTr | **Brain activity ￫ GAF** | No relationship | 21.0 | 23 | 0.58 | 1.00 | 0.00 | 30322.0 | 30488.9 |
|  | IFGOp | **Brain activity ￫ GAF** | No relationship | 21.1 | 23 | 0.57 | 1.00 | 0.00 | 30291.3 | 30458.2 |
|  | IFGOr | Brain activity ￫ GAF | **Task performance ￫ Brain activity** | 19.7 | 22 | 0.60 | 1.00 | 0.00 | 30077.4 | 30248.3 |
|  | STG | **Brain activity ￫ GAF** | No relationship | 18.3 | 23 | 0.74 | 1.00 | 0.00 | 30466.5 | 30633.4 |
|  | MTG | **Brain activity ￫ GAF** | No relationship | 18.9 | 23 | 0.71 | 1.00 | 0.00 | 30427.0 | 30593.9 |

The best fit model using the AIC from 16 models was listed for each brain region. Bold shows a relationship with .05 or smaller of p value. Abbreviation: SFG, superior frontal gyrus; SFGM, superior frontal medial cortex; MFG, middle frontal gyrus; IFGTr, inferior frontal gyrus triangularis; IFGOp, inferior frontal gyrus opercularis; IFGOr, inferior frontal gyrus orbital; STG, superior temporal gyrus; MTG, middle temporal gyrus.

## **Supplementary Table S29. A list of the best fit models including the PANSS positive subscale in the SEM model comparison of the schizophrenia group**

| Region | | Brain activityｰPANSS relationship | Brain activityｰTask performance relationship | Chi square | df | p value | CFI | RMSEA | AIC | BIC |
| --- | --- | --- | --- | --- | --- | --- | --- | --- | --- | --- |
| Right | SFG | No relationship | Brain activity ￫ Task performance | 25.1 | 27 | 0.57 | 1.00 | 0.000 | 14382.5 | 14507.0 |
|  | SFGM | No relationship | **Brain activity ￫ Task performance** | 24.8 | 27 | 0.58 | 1.00 | 0.000 | 14350.2 | 14474.7 |
|  | MFG | No relationship | Brain activity ￫ Task performance | 25.4 | 27 | 0.55 | 1.00 | 0.000 | 14381.7 | 14506.3 |
|  | IFGTr | No relationship | No relationship | 27.9 | 28 | 0.47 | 1.00 | 0.000 | 14619.2 | 14740.5 |
|  | IFGOp | No relationship | No relationship | 29.1 | 28 | 0.41 | 0.99 | 0.014 | 14551.1 | 14672.4 |
|  | IFGOr | No relationship | No relationship | 26.5 | 28 | 0.55 | 1.00 | 0.000 | 14092.5 | 14213.8 |
|  | STG | No relationship | No relationship | 28.3 | 28 | 0.45 | 1.00 | 0.007 | 14574.4 | 14695.6 |
|  | MTG | No relationship | No relationship | 27.7 | 28 | 0.48 | 1.00 | 0.000 | 14344.9 | 14466.2 |
| Left | SFG | No relationship | Brain activity ￫ Task performance | 26.9 | 27 | 0.47 | 1.00 | 0.000 | 14444.7 | 14569.2 |
|  | SFGM | No relationship | **Brain activity ￫ Task performance** | 25.9 | 27 | 0.52 | 1.00 | 0.000 | 14366.3 | 14490.8 |
|  | MFG | No relationship | **Brain activity ￫ Task performance** | 24.5 | 27 | 0.60 | 1.00 | 0.000 | 14392.4 | 14517.0 |
|  | IFGTr | Brain activity ￫ PANSS Positive | Brain activity ￫ Task performance | 25.0 | 26 | 0.52 | 1.00 | 0.000 | 14460.2 | 14588.0 |
|  | IFGOp | No relationship | Brain activity ￫ Task performance | 25.5 | 27 | 0.55 | 1.00 | 0.000 | 14437.9 | 14562.5 |
|  | IFGOr | No relationship | **Brain activity ￫ Task performance** | 25.4 | 27 | 0.55 | 1.00 | 0.000 | 14334.7 | 14459.3 |
|  | STG | No relationship | No relationship | 27.5 | 28 | 0.49 | 1.00 | 0.000 | 14533.9 | 14655.2 |
|  | MTG | **Brain activity ￫ PANSS Positive** | No relationship | 25.9 | 27 | 0.52 | 1.00 | 0.000 | 14630.2 | 14754.8 |

The best fit model using the AIC from 16 models was listed for each brain region. Bold shows a relationship with .05 or smaller of p value.

Abbreviation: SFG, superior frontal gyrus; SFGM, superior frontal medial cortex; MFG, middle frontal gyrus; IFGTr, inferior frontal gyrus triangularis; IFGOp, inferior frontal gyrus opercularis; IFGOr, inferior frontal gyrus orbital; STG, superior temporal gyrus; MTG, middle temporal gyrus.

## **Supplementary Table S30. A list of the best fit models including the PANSS negative subscale in the SEM model comparison of the schizophrenia group**

| Region | | Brain activityｰPANSS relationship | Brain activityｰTask performance relationship | Chi square | df | p value | CFI | RMSEA | AIC | BIC |
| --- | --- | --- | --- | --- | --- | --- | --- | --- | --- | --- |
| Right | SFG | No relationship | Brain activity ￫ Task performance | 25.8 | 27 | 0.53 | 1.00 | 0.000 | 14477.7 | 14602.2 |
|  | SFGM | No relationship | **Brain activity ￫ Task performance** | 27.0 | 27 | 0.46 | 1.00 | 0.000 | 14446.3 | 14570.8 |
|  | MFG | No relationship | Brain activity ￫ Task performance | 26.4 | 27 | 0.50 | 1.00 | 0.000 | 14476.8 | 14601.4 |
|  | IFGTr | No relationship | No relationship | 29.5 | 28 | 0.39 | 0.99 | 0.016 | 14714.8 | 14836.1 |
|  | IFGOp | No relationship | Brain activity ￫ Task performance | 29.1 | 27 | 0.36 | 0.98 | 0.019 | 14646.2 | 14770.8 |
|  | IFGOr | No relationship | No relationship | 28.8 | 28 | 0.42 | 0.99 | 0.012 | 14188.2 | 14309.5 |
|  | STG | No relationship | No relationship | 30.4 | 28 | 0.34 | 0.98 | 0.020 | 14670.0 | 14791.3 |
|  | MTG | **Brain activity ￫ PANSS negative** | No relationship | 26.6 | 27 | 0.48 | 1.00 | 0.000 | 14439.8 | 14564.4 |
| Left | SFG | No relationship | Brain activity ￫ Task performance | 27.3 | 27 | 0.45 | 1.00 | 0.007 | 14539.9 | 14664.5 |
|  | SFGM | No relationship | **Brain activity ￫ Task performance** | 25.8 | 27 | 0.53 | 1.00 | 0.000 | 14460.8 | 14585.4 |
|  | MFG | No relationship | **Brain activity ￫ Task performance** | 26.2 | 27 | 0.51 | 1.00 | 0.000 | 14488.3 | 14612.9 |
|  | IFGTr | No relationship | Brain activity ￫ Task performance | 26.2 | 27 | 0.51 | 1.00 | 0.000 | 14555.0 | 14679.5 |
|  | IFGOp | No relationship | Brain activity ￫ Task performance | 26.8 | 27 | 0.48 | 1.00 | 0.000 | 14533.2 | 14657.8 |
|  | IFGOr | No relationship | **Brain activity ￫ Task performance** | 26.0 | 27 | 0.52 | 1.00 | 0.000 | 14429.5 | 14554.1 |
|  | STG | No relationship | No relationship | 28.8 | 28 | 0.42 | 0.99 | 0.012 | 14629.6 | 14750.9 |
|  | MTG | No relationship | No relationship | 28.9 | 28 | 0.42 | 0.99 | 0.013 | 14727.6 | 14848.9 |

The best fit model using the AIC from 16 models was listed for each brain region. Bold shows a relationship with .05 or smaller of p value.

Abbreviation: SFG, superior frontal gyrus; SFGM, superior frontal medial cortex; MFG, middle frontal gyrus; IFGTr, inferior frontal gyrus triangularis; IFGOp, inferior frontal gyrus opercularis; IFGOr, inferior frontal gyrus orbital; STG, superior temporal gyrus; MTG, middle temporal gyrus.

## **Supplementary Table S31. A list of the best fit models including the PANSS general psychopathology subscale in the SEM model comparison of the schizophrenia group**

| Region | | Brain activityｰPANSS relationship | Brain activityｰTask performance relationship | Chi square | df | p value | CFI | RMSEA | AIC | BIC |
| --- | --- | --- | --- | --- | --- | --- | --- | --- | --- | --- |
| Right | SFG | No relationship | Task performance ￫ Brain activity | 26.0 | 28 | 0.58 | 1.00 | 0.00 | 14602.7 | 14724.0 |
|  | SFGM | No relationship | **Task performance ￫ Brain activity** | 25.5 | 28 | 0.60 | 1.00 | 0.00 | 14570.6 | 14691.8 |
|  | MFG | No relationship | Task performance ￫ Brain activity | 26.3 | 28 | 0.56 | 1.00 | 0.00 | 14601.8 | 14723.1 |
|  | IFGTr | No relationship | No relationship | 26.6 | 28 | 0.54 | 1.00 | 0.00 | 14840.0 | 14961.3 |
|  | IFGOp | No relationship | Task performance ￫ Brain activity | 27.8 | 28 | 0.48 | 1.00 | 0.00 | 14771.2 | 14892.5 |
|  | IFGOr | Brain activity ￫ PANSS general psychopathology | No relationship | 23.5 | 27 | 0.66 | 1.00 | 0.00 | 14312.8 | 14437.4 |
|  | STG | PANSS general psychopathology ￫ Brain activity | No relationship | 26.6 | 28 | 0.54 | 1.00 | 0.00 | 14794.9 | 14916.2 |
|  | MTG | PANSS general psychopathology ￫ Brain activity | No relationship | 25.2 | 28 | 0.62 | 1.00 | 0.00 | 14563.7 | 14685.0 |
| Left | SFG | No relationship | Task performance ￫ Brain activity | 26.9 | 28 | 0.52 | 1.00 | 0.00 | 14664.5 | 14785.8 |
|  | SFGM | No relationship | **Brain activity ￫ Task performance** | 24.6 | 27 | 0.60 | 1.00 | 0.00 | 14585.6 | 14710.2 |
|  | MFG | No relationship | Task performance ￫ Brain activity | 25.6 | 28 | 0.60 | 1.00 | 0.00 | 14613.0 | 14734.3 |
|  | IFGTr | No relationship | Task performance ￫ Brain activity | 26.2 | 28 | 0.56 | 1.00 | 0.00 | 14680.1 | 14801.4 |
|  | IFGOp | No relationship | Task performance ￫ Brain activity | 25.8 | 28 | 0.58 | 1.00 | 0.00 | 14658.3 | 14779.6 |
|  | IFGOr | No relationship | **Task performance ￫ Brain activity** | 26.1 | 28 | 0.57 | 1.00 | 0.00 | 14554.6 | 14675.9 |
|  | STG | Brain activity ↔ PANSS general psychopathology | No relationship | 25.4 | 28 | 0.61 | 1.00 | 0.00 | 14754.6 | 14875.9 |
|  | MTG | PANSS general psychopathology ￫ Brain activity | No relationship | 25.4 | 28 | 0.60 | 1.00 | 0.00 | 14852.1 | 14973.4 |

The best fit model using the AIC from 16 models was listed for each brain region. Bold shows a relationship with .05 or smaller of p value.

Abbreviation: SFG, superior frontal gyrus; SFGM, superior frontal medial cortex; MFG, middle frontal gyrus; IFGTr, inferior frontal gyrus triangularis; IFGOp, inferior frontal gyrus opercularis; IFGOr, inferior frontal gyrus orbital; STG, superior temporal gyrus; MTG, middle temporal gyrus.

## **Supplementary Table S32. A list of the best fit models including the YMRS scale in the SEM model comparison of the BPD group**

| Region | | Brain activityｰYMRS relationship | Brain activityｰTask performance relationship | Chi square | df | p value | CFI | RMSEA | AIC | BIC |
| --- | --- | --- | --- | --- | --- | --- | --- | --- | --- | --- |
| Right | SFG | No relationship | No relationship | 17.5 | 26 | 0.89 | 1.00 | 0.00 | 14339.2 | 14465.6 |
|  | SFGM | No relationship | Brain activity ￫ Task performance | 15.5 | 25 | 0.93 | 1.00 | 0.00 | 14241.6 | 14371.2 |
|  | MFG | No relationship | **Brain activity ￫ Task performance** | 15.1 | 25 | 0.94 | 1.00 | 0.00 | 14305.3 | 14435.0 |
|  | IFGTr | No relationship | Task performance ￫ Brain activity | 15.5 | 25 | 0.93 | 1.00 | 0.00 | 14405.9 | 14535.6 |
|  | IFGOp | No relationship | No relationship | 15.7 | 26 | 0.94 | 1.00 | 0.00 | 14369.4 | 14495.8 |
|  | IFGOr | YMRS ￫ Brain activity | No relationship | 14.6 | 25 | 0.95 | 1.00 | 0.00 | 13987.5 | 14117.2 |
|  | STG | No relationship | No relationship | 16.4 | 26 | 0.93 | 1.00 | 0.00 | 14414.2 | 14540.6 |
|  | MTG | No relationship | No relationship | 15.8 | 26 | 0.94 | 1.00 | 0.00 | 14279.0 | 14405.4 |
| Left | SFG | No relationship | **Task performance ￫ Brain activity** | 15.1 | 25 | 0.94 | 1.00 | 0.00 | 14270.3 | 14400.0 |
|  | SFGM | No relationship | **Brain activity ￫ Task performance** | 15.5 | 25 | 0.93 | 1.00 | 0.00 | 14336.3 | 14466.0 |
|  | MFG | No relationship | **Task performance ￫ Brain activity** | 15.7 | 25 | 0.92 | 1.00 | 0.00 | 14305.3 | 14435.0 |
|  | IFGTr | No relationship | No relationship | 18.4 | 26 | 0.86 | 1.00 | 0.00 | 14360.5 | 14486.9 |
|  | IFGOp | No relationship | No relationship | 18.4 | 26 | 0.86 | 1.00 | 0.00 | 14352.4 | 14478.8 |
|  | IFGOr | YMRS ￫ Brain activity | No relationship | 16.8 | 25 | 0.89 | 1.00 | 0.00 | 14309.0 | 14438.7 |
|  | STG | No relationship | No relationship | 14.9 | 26 | 0.96 | 1.00 | 0.00 | 14457.9 | 14584.3 |
|  | MTG | No relationship | No relationship | 15.9 | 26 | 0.94 | 1.00 | 0.00 | 14472.1 | 14598.5 |

The best fit model using the AIC from 16 models was listed for each brain region. Bold shows a relationship with .05 or smaller of p value. Abbreviation: SFG, superior frontal gyrus; SFGM, superior frontal medial cortex; MFG, middle frontal gyrus; IFGTr, inferior frontal gyrus triangularis; IFGOp, inferior frontal gyrus opercularis; IFGOr, inferior frontal gyrus orbital; STG, superior temporal gyrus; MTG, middle temporal gyrus.

## **Supplementary Table S33. A list of the best fit models including the HAM-D scale in the SEM model comparison of the BPD group**

| Region | | Brain activityｰHAMD relationship | Brain activityｰTask performance relationship | Chi square | df | p value | CFI | RMSEA | AIC | BIC |
| --- | --- | --- | --- | --- | --- | --- | --- | --- | --- | --- |
| Right | SFG | No relationship | No relationship | 22.0 | 24 | 0.58 | 1.00 | 0.00 | 14536.0 | 14668.9 |
|  | SFGM | HAMD ￫ Brain activity | Brain activity ￫ Task performance | 19.7 | 22 | 0.60 | 1.00 | 0.00 | 14438.0 | 14577.3 |
|  | MFG | No relationship | **Brain activity ￫ Task performance** | 20.3 | 23 | 0.63 | 1.00 | 0.00 | 14502.2 | 14638.3 |
|  | IFGTr | Brain activity ￫ HAMD | Task performance ￫ Brain activity | 19.7 | 22 | 0.60 | 1.00 | 0.00 | 14601.8 | 14741.2 |
|  | IFGOp | No relationship | No relationship | 20.6 | 24 | 0.66 | 1.00 | 0.00 | 14566.2 | 14699.2 |
|  | IFGOr | **Brain activity ￫ HAMD** | No relationship | 19.2 | 23 | 0.69 | 1.00 | 0.00 | 14183.3 | 14319.5 |
|  | STG | No relationship | No relationship | 20.9 | 24 | 0.65 | 1.00 | 0.00 | 14611.0 | 14743.9 |
|  | MTG | No relationship | No relationship | 20.6 | 24 | 0.66 | 1.00 | 0.00 | 14475.8 | 14608.7 |
| Left | SFG | No relationship | **Task performance ￫ Brain activity** | 22.4 | 23 | 0.50 | 1.00 | 0.00 | 14467.2 | 14603.3 |
|  | SFGM | No relationship | **Brain activity ￫ Task performance** | 21.2 | 23 | 0.57 | 1.00 | 0.00 | 14533.2 | 14669.4 |
|  | MFG | No relationship | **Task performance ￫ Brain activity** | 20.8 | 23 | 0.59 | 1.00 | 0.00 | 14502.2 | 14638.4 |
|  | IFGTr | No relationship | No relationship | 21.8 | 24 | 0.59 | 1.00 | 0.00 | 14557.4 | 14690.3 |
|  | IFGOp | No relationship | No relationship | 22.1 | 24 | 0.57 | 1.00 | 0.00 | 14549.2 | 14682.1 |
|  | IFGOr | No relationship | No relationship | 22.7 | 24 | 0.54 | 1.00 | 0.00 | 14505.9 | 14638.9 |
|  | STG | No relationship | No relationship | 20.5 | 24 | 0.67 | 1.00 | 0.00 | 14654.7 | 14787.6 |
|  | MTG | No relationship | No relationship | 21.6 | 24 | 0.60 | 1.00 | 0.00 | 14668.9 | 14801.8 |

The best fit model using the AIC from 16 models was listed for each brain region. Bold shows a relationship with .05 or smaller of p value. Abbreviation: SFG, superior frontal gyrus; SFGM, superior frontal medial cortex; MFG, middle frontal gyrus; IFGTr, inferior frontal gyrus triangularis; IFGOp, inferior frontal gyrus opercularis; IFGOr, inferior frontal gyrus orbital; STG, superior temporal gyrus; MTG, middle temporal gyrus.

## **Supplementary Table S34. A list of the best fit models including the HAM-D scale in the SEM model comparison of the MDD group**

| Region | | Brain activityｰHAMD relationship | Brain activityｰTask performance relationship | Chi square | df | p value | CFI | RMSEA | AIC | BIC |
| --- | --- | --- | --- | --- | --- | --- | --- | --- | --- | --- |
| Right | SFG | No relationship | **Task performance ↔ Brain activity** | 25.2 | 26 | 0.51 | 1.00 | 0.00 | 29814.1 | 29969.1 |
|  | SFGM | No relationship | Brain activity ￫ Task performance | 24.2 | 26 | 0.56 | 1.00 | 0.00 | 29671.0 | 29826.1 |
|  | MFG | Brain activity ↔ HAMD | Task performance ↔ Brain activity | 23.7 | 25 | 0.53 | 1.00 | 0.00 | 29819.8 | 29978.8 |
|  | IFGTr | No relationship | No relationship | 24.0 | 27 | 0.63 | 1.00 | 0.00 | 29953.5 | 30104.6 |
|  | IFGOp | No relationship | No relationship | 24.9 | 27 | 0.58 | 1.00 | 0.00 | 29842.5 | 29993.6 |
|  | IFGOr | No relationship | Brain activity ￫ Task performance | 23.5 | 26 | 0.60 | 1.00 | 0.00 | 29196.9 | 29351.9 |
|  | STG | No relationship | No relationship | 23.5 | 27 | 0.66 | 1.00 | 0.00 | 29977.6 | 30128.7 |
|  | MTG | No relationship | No relationship | 24.0 | 27 | 0.63 | 1.00 | 0.00 | 29682.1 | 29833.2 |
| Left | SFG | No relationship | Brain activity ￫ Task performance | 23.8 | 26 | 0.59 | 1.00 | 0.00 | 29715.2 | 29870.2 |
|  | SFGM | No relationship | Brain activity ￫ Task performance | 23.8 | 26 | 0.59 | 1.00 | 0.00 | 29788.8 | 29943.9 |
|  | MFG | No relationship | **Task performance ↔ Brain activity** | 24.5 | 26 | 0.55 | 1.00 | 0.00 | 29712.1 | 29867.2 |
|  | IFGTr | No relationship | No relationship | 25.6 | 27 | 0.54 | 1.00 | 0.00 | 29877.0 | 30028.1 |
|  | IFGOp | No relationship | No relationship | 26.7 | 27 | 0.48 | 1.00 | 0.00 | 29845.3 | 29996.4 |
|  | IFGOr | No relationship | **Task performance ↔ Brain activity** | 23.6 | 26 | 0.60 | 1.00 | 0.00 | 29630.4 | 29785.5 |
|  | STG | No relationship | No relationship | 23.6 | 27 | 0.65 | 1.00 | 0.00 | 30028.0 | 30179.1 |
|  | MTG | No relationship | No relationship | 23.7 | 27 | 0.65 | 1.00 | 0.00 | 29989.2 | 30140.3 |

The best fit model using the AIC from 16 models was listed for each brain region. Bold shows a relationship with .05 or smaller of p value. Abbreviation: SFG, superior frontal gyrus; SFGM, superior frontal medial cortex; MFG, middle frontal gyrus; IFGTr, inferior frontal gyrus triangularis; IFGOp, inferior frontal gyrus opercularis; IFGOr, inferior frontal gyrus orbital; STG, superior temporal gyrus; MTG, middle temporal gyrus.
