# Supplementary figures and images for: Shared functional impairment in the prefrontal cortex affects symptom severity across psychiatric disorders
Source: Psychol Med. 2020 Dec 18;52(13):2661–70. doi: 10.1017/S0033291720004742 (PMC9647535; doi:10.1017/S0033291720004742)

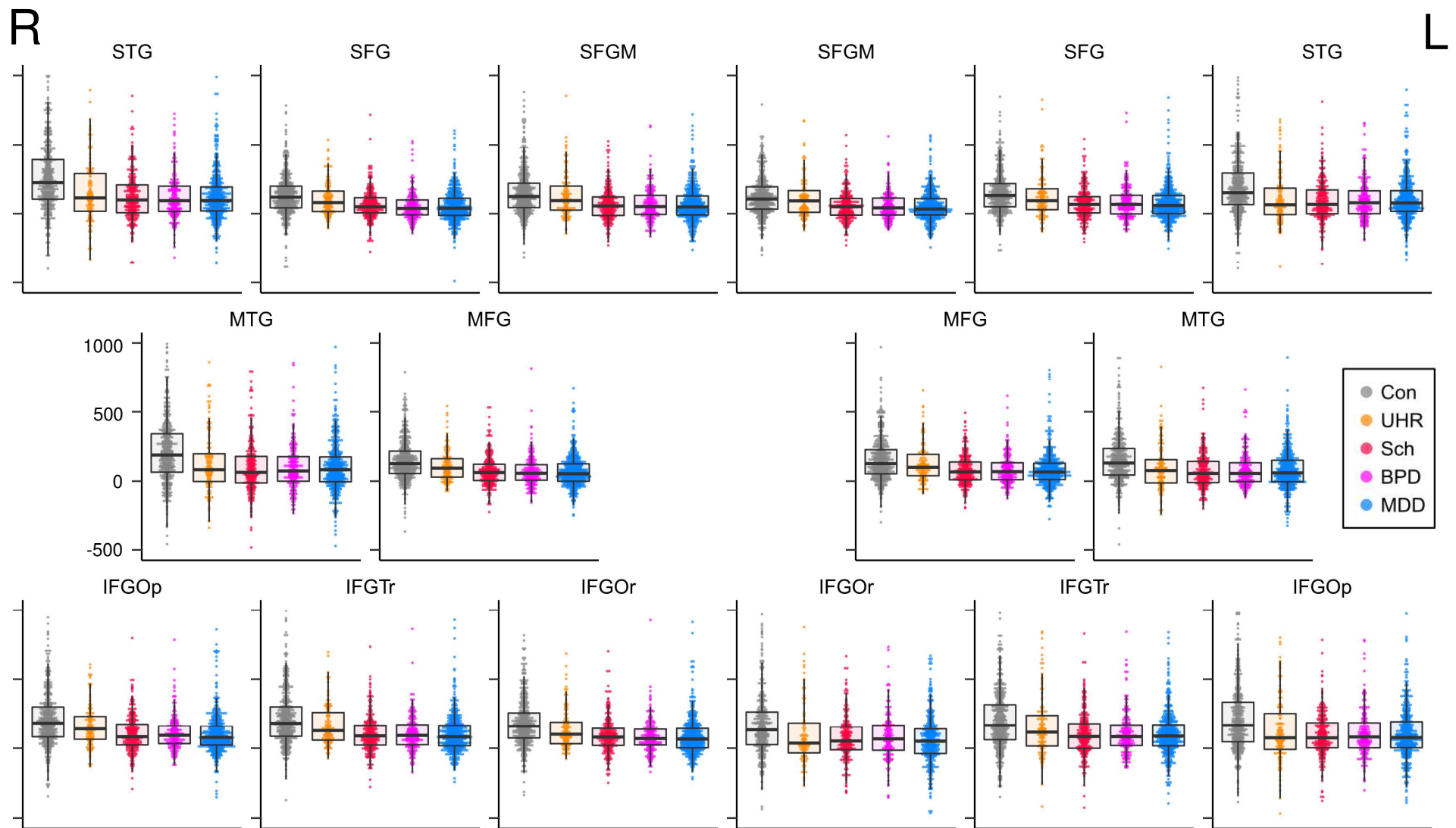

Koike et al. Supplementary Figure S2

Supplement: Supplementary file 1 [file S0033291720004742sup.zip › S0033291720004742sup001.pdf]
